# Candidate orphan genes: Reassessing uniqueness

**DOI:** 10.1371/journal.pone.0338891

**Published:** 2025-12-31

**Authors:** Sheetalpreet K. Maan, Xuan Y. Butzin, Steven X. Ge, Nicholas C. Butzin

**Affiliations:** 1 Department of Biology and Microbiology, South Dakota State University, Brookings, South Dakota, United States of America; 2 CAI-SMART Center, South Dakota State University, Brookings, South Dakota, United States of America; 3 South Dakota Mines, Rapid City, South Dakota, United States of America; 4 University of Nevada, Las Vegas, Nevada, United States of America; 5 Department of Mathematics & Statistics, South Dakota State University, Brookings, South Dakota, United States of America; 6 Department of Chemistry and Biochemistry, South Dakota State University, Brookings, South Dakota, United States of America; University of Essex, UNITED KINGDOM OF GREAT BRITAIN AND NORTHERN IRELAND

## Abstract

Orphan genes lack recognizable homologues outside a given taxonomic unit; thus, they have uncertain evolutionary origins. This presents a profound challenge to traditional models of gene evolution. Their presence has fueled ongoing debate, and they have long been implicated in driving lineage-specific traits in medicine and evolutionary biology. These genes are often linked to species-specific traits and pathogenic mechanisms, including virulence and environmental adaptation, and their study provides critical insights into the origins and evolution of novel genes. Intrigued by their enigmatic nature, we re-analyzed a comprehensive 2023 dataset of orphan genes compiled from over 80,000 bacterial species. Using homology-based analyses, we reassessed the taxonomic distribution of each gene across a broader genomic landscape. Many “orphan genes” identified in 2023 now align with homologs in other bacterial taxa (as of 2025), demonstrating that limited database sampling had previously inflated the number of genes. This reassessment revealed an approximately 81% decrease in the number of orphan genes within just two years. These results challenged the long-held view that bacterial species truly harbor large numbers of orphan genes, instead demonstrating that their prevalence has been overestimated. To better reflect these findings, we propose that orphan genes be annotated using descriptors such as ‘candidate’ or ‘putative’, which more accurately convey the provisional and potentially temporary nature of their apparent uniqueness. Although our analysis greatly reduced false-positive classifications, it cannot determine whether a given candidate truly encodes a functional gene or is an artifact of bioinformatic analysis. To prioritize the most promising targets for biochemical or genetic validation, we applied additional computational filters and identified a subset of candidates most likely to encode bona fide proteins. This study redefines current understanding of orphan gene prevalence, establishes that such genes should be annotated with descriptors such as candidate or putative, much like we label “candidate bacterial species,” and provides a refined, high-confidence dataset for future *in vitro* and *in vivo* investigations.

## Introduction

Most genes have homologs in other organisms. However, orphan genes (also called ORFans or Taxonomically Restricted Genes) have been defined as genes with no detectable homology outside a specific species or lineage [1]. These genes are often thought to influence speciation and evolutionary diversification by providing organisms with novel traits that facilitate adaptation to changing environments [2]. There are several mechanisms reported in the literature that explain the origin of such genes, including horizontal gene transfer, duplication and divergence, and *de* *novo* origination [3–5]. Interestingly, orphan genes have been identified across various life forms, such as vertebrates [6,7], insects [8], nematodes [9], yeast [10], plants [11], and bacteria.

Despite their broad distribution, most orphan genes remain functionally unannotated due to the absence of detectable evolutionary history. Consequently, many are categorized as hypothetical proteins, representing a significant portion of what is called the “dark genome.” The dark genome consists of poorly understood and uncharacterized regions of genomes lacking clear functional assignments. This includes both genes that have been computationally predicted but remain uncharacterized, and genes that likely exist but have not yet been predicted or annotated. Recent research has revitalized efforts to determine whether the hypothetical genes are functional and, if so, what traits they encode. A study showed that dark genes have high mutation rates in certain cancers and impact patient survival [12]. Their critical vulnerabilities suggest promising therapeutic targets, emphasizing the need for further research.

In this study, we investigated orphan genes across 10 different bacterial species and demonstrated that as more genomes are sequenced, previously identified orphan genes lose their unique status. This pattern indicates that orphan genes are frequently overestimated, likely due to the limited and biased sample of genomes currently available. Given the results from this work, we prefer to use descriptors such as ‘candidate’ or ‘putative’, which more accurately convey the provisional and potentially temporary nature of their apparent uniqueness. This reflects the reality that many genes previously classified as orphan genes are not truly unique to a lineage. We anticipate that with continued sequencing efforts, more genes will similarly lose their orphan status.

**The objectives of this study:**

1. To systematically re-evaluate bacterial orphan genes across multiple species using updated genomic datasets.
2. To refine orphan gene terminology in light of expanding sequence data.
3. To propose a computational framework for prioritizing candidate orphan genes for experimental validation and functional characterization.

Together, these efforts aim to clarify the evolutionary and biological significance of these enigmatic genes, thereby guiding future experimental exploration.

## Results and discussion

**Fig 1:**
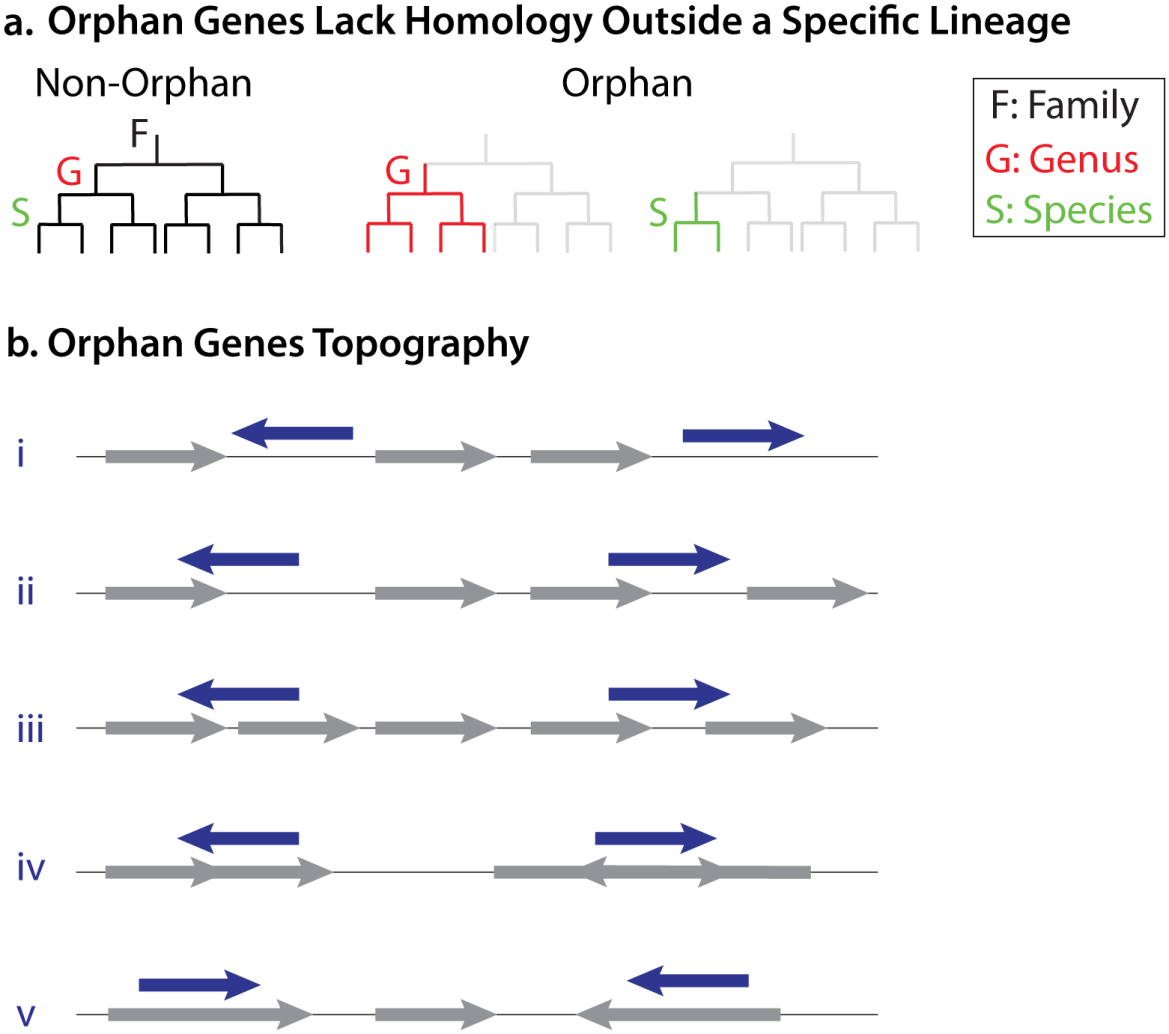
Classification and Genomic Topography of Orphan Genes. **a.** Distinguishing orphan genes from non-orphan genes. Orphan genes are characterized by their restricted taxonomic distribution. If a gene is present at the genus level but absent outside that genus, it is classified as a genus-specific orphan gene. Similarly, a species-specific orphan gene is identified only within a single species and not in other species. These genes exhibit no detectable homology outside their respective taxonomic levels. **b.** Genomic topography of orphan genes. Orphan genes can be found in various genomic locations: **i.** intergenic regions (between two genes), **ii.** overlapping a single gene, **iii.** overlapping two genes, **iv.** overlapping two overlapping genes, and **v.** located within a gene. Some of the genes were already annotated in the NCBI data, while others (the majority) were unannotated. The annotation status of each gene was determined by manually inspecting the genomes in Geneious after downloading them from NCBI.

**Advanced Screening Approach Led to a Reduction in the Number of Potential Orphan Genes.** Orphan genes differ from non-orphan genes in their taxonomic distribution. While non-orphan genes can be found across multiple species, genera, or other taxonomic units, orphan genes are typically confined to a specific taxonomic unit. If an orphan gene is restricted to a single genus, it is classified as a genus-level orphan gene, whereas if it is found only within a particular species, it is categorized as a species-level orphan gene (Fig 1a). In this study, we focused on species-level orphan genes from selected bacterial species. These genes were distributed across various genomic locations, underscoring the complexity associated with their presence and organization (Fig 1b). To distinguish genuine orphan genes from potential artifacts of bioinformatic predictions, we reassessed the orphan genes reported in TRGdb [13] (a database of bacterial orphan genes predicted in 2023) using the BLASTp [14] approach.

**Fig 2:**
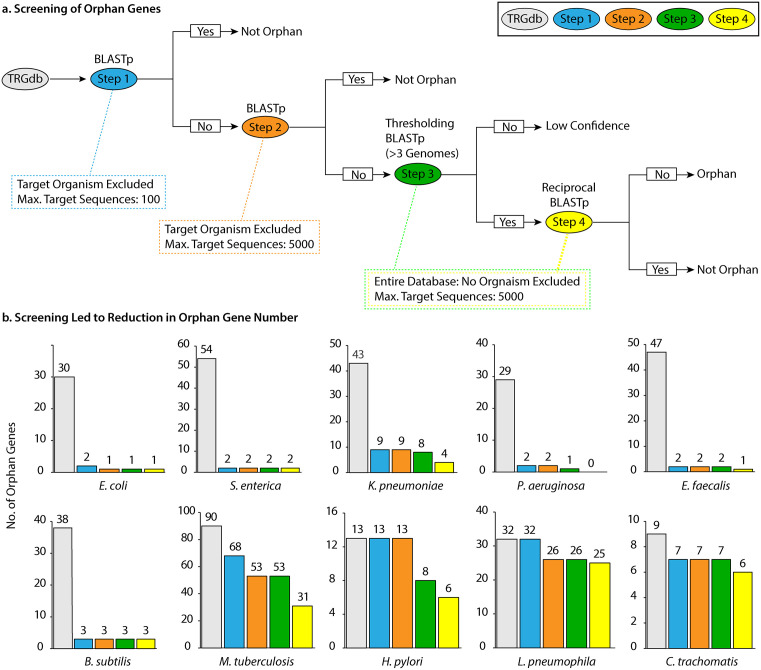
Stepwise BLASTp Screening Reveals Reduction of Orphan Genes Across Bacterial Species. **a.** Schematic Representation of the Screening Process. Step 1: BLASTp was performed on protein sequences obtained from TRGdb, excluding the target organism from the search. The maximum target sequences parameter was set to the default value of 100. Step 2: Protein sequences identified as orphan genes in Step 1 were reanalyzed using BLASTp with an increased maximum target sequence limit of 5000. Step 3 (**Thresholding BLASTp**): Sequences that remained orphan genes in Step 2 were searched again, this time including the target organism. A threshold of “3 genomes” was applied, meaning that proteins detected in more than 3 genomes of the same species were considered for further analysis. Step 4 (**Reciprocal BLASTp**): For sequences that were detected in more than 3 genomes in Step 3, the resulting protein hits were examined. Among these, the protein with the lowest sequence similarity to the query in Step 3 was selected and used as the query in a reciprocal BLASTp search against the database. E value was set to 1E-3 for all four steps. **b.** Reduction in orphan gene numbers across screening steps. The screening process resulted in a progressive decrease in the number of orphan genes across 10 bacterial species: *E. coli*, *S. enterica*, *K. pneumoniae*, *P. aeruginosa*, *E. faecalis*, *B. subtilis*, *M. tuberculosis*, *H. pylori*, *L. pneumophila*, and *C. trachomatis*. A marked reduction in orphan gene numbers was observed across all species within just two years, with the most remarkable decline occurring in Step 1.

The analysis covered 10 bacterial species: *Escherichia coli*, *Salmonella enterica*, *Klebsiella pneumoniae*, *Pseudomonas aeruginosa*, *Enterococcus faecalis*, *Bacillus subtilis, Mycobacterium tuberculosis*, *Helicobacter pylori*, *Legionella pneumophila*, and *Chlamydia trachomatis*. Our results demonstrated that for all bacterial species studied, most of the orphan genes lost their status due to the detection of significant similarity in other bacterial genera, families, or taxonomic units. Consequently, it led to their exclusion from further study. The BLASTp approach employed 4 steps (E value: 1E-3) to ensure the reliability of our results (Fig 2a). Each step was designed to progressively refine our identification of candidate orphan genes by checking for sequence similarity across different levels of relatedness.

### Step 1

In the first phase of the BLAST-based screening, our goal was to determine whether previously identified orphan genes in one species, identified two years ago, were still unique to that species. A large proportion of the putative orphan genes were not unique to the original species as orphans in TRGdb shared detectable homology when re-evaluated (Fig 2b). The number of target sequences was kept at the default BLAST setting (100) to optimize computational efficiency and reduce processing time. This reduced the number of hits considered. Only those genes that showed no similarity at the 100-target threshold were taken to Step 2.

### Step 2

After identifying initial orphan gene candidates, we next sought to determine whether some of these genes had simply escaped detection due to conservative BLAST search parameters. Step 2 re-examined all remaining candidates by altering the “maximum target sequences.” The increase in the “maximum target sequences” to the maximum limit allowed by NCBI allowed detection of homologs for some genes that exhibited no similarity in the first round. The count of orphan genes declined once more in Step 2 (Fig 2b). We opted not to perform Step 2 in the first place due to the intense computational demands associated with the high number of target sequences. Step 1 facilitated quicker elimination of genes that lost their status as “orphans” by reducing processing time. The longer processing time required for Step 2 made it a more suitable follow-up step after narrowing down the orphan genes. The difference in results when increasing the “maximum target sequences” in BLASTp is because of how BLAST processes its search set. BLAST does not merely return the first N hits that exceed the specified E-value threshold [15]. The BLAST search is divided into two phases. First, the database search phase, where the query sequence is divided into subsequences (smaller words), and these words are then compared to target sequences in the database. Second, the alignment phase, where BLAST executes detailed alignment of these selected target sequences with the query [14]. The former step is designed to rapidly identify the most promising target sequences that are likely to produce meaningful alignments. When the “maximum target sequences” parameter is set to a lower value, the search gets restricted to a subset of the most promising sequences, optimizing speed but missing potential homologs. By increasing the value to 5,000, BLAST could detect additional homologs that may not have been identified in an initial, smaller search. Although this leads to an increase in computational time, it enables a more comprehensive comparison, thereby enhancing the sensitivity of the alignment. This emphasizes the trade-off between computational efficiency and sensitivity in database searches.

### Step 3

To filter out low-confidence predictions, we applied a BLASTp thresholding approach in which any ORF detected in three or fewer genomes of the target species was classified as a low-confidence orphan gene (Fig 2a). This filtering step serves several important purposes.

First, many bacterial gene entries in NCBI, including coding sequences (CDSs) and open reading frames (ORFs), originate from automated annotation pipelines that classify any sufficiently long ORF as a “gene.” As a result, sequencing or assembly artifacts (e.g., misassembled contigs or repetitive regions) can yield spurious ORFs that (without supporting RNA-seq or proteomic evidence) remain hypothetical and may never correspond to true expressed proteins.

Second, mis-assembly, contamination, and mis-annotation remain pervasive issues in bacterial genome data, frequently leading to the prediction of false “genes” that disappear following resequencing or improved assembly. In several cases, re-sequenced genomes show a substantial reduction in annotated genes due to the removal of contaminant sequences (most commonly originating from human or laboratory sources). Recent studies have demonstrated that draft bacterial assemblies can contain hundreds of contaminant contigs derived from foreign species, resulting directly from sequencing and assembly errors [16]. Similarly, human contamination has been shown to generate thousands of spurious protein-coding predictions in bacterial assemblies, which were subsequently propagated into public databases [17].

More recently, the NCBI FCS-GX cleanup effort removed thousands of contaminated prokaryotic genomes and reported that most contaminant sequences (and consequently, most erroneous genes) were short in length [18]. It was also reported that predicted orphan genes are often shorter than non-orphan genes [19]. This overlap between short sequence length and higher error susceptibility underscores why many predicted orphan genes may, in fact, reflect annotation or assembly artifacts rather than true novel genes.

Because the current databases are not free of false positives, predicted small orphan genes could include spurious entries. Step 3 helps minimize the likelihood of such false positives. Although this filtering may eliminate a small number of true genes, ongoing genome sequencing efforts will enable future corrections and refinements. Overall, Step 3 resulted in the exclusion of only seven potential orphan genes from the entire dataset, representing a minimal reduction in the total number of candidates (Fig 2b).

### Step 4

To ensure that no distant homologs were missed in our initial screens, Step 4 employed a reciprocal BLASTp analysis [20,21] with an E-value cutoff of 1E-3. The E-value represents the expected number of alignments with a given score that could occur by chance when searching a query against the database; thus, a threshold of 1E-3 retains only hits with ≤ 0.001 probability of arising randomly. This commonly used cutoff [22] was also applied in the TRGdb database for gene identification [13], allowing direct comparison between studies. We deliberately used the weakest Step 3 match to cast a wider net for remote homologs for our reciprocal BLAST. Any gene that returned a hit with an E-value cutoff of 1E-3 to another organism in this reciprocal search was removed from our candidate list. This extra layer is vital; without it, highly divergent genes or those with low-complexity regions can masquerade as true orphans. Applying this strategy further refined our dataset, leading to an even greater reduction in orphan genes (Fig 2b).

### Steps 1–4

Through our screening method, the four intracellular pathogens we examined (*M. tuberculosis*, *H. pylori*, *L. pneumophila*, and *C. trachomatis*) had reduced orphan genes, but this decline was smaller compared to bacteria from broader environmental niches (Fig 2b). The percentage decline in orphan gene count for bacteria with a broad environmental habitat ranged from 90% to 100%. In contrast, for four intracellular pathogens, the decline was less, ranging from 28% to 66%.

We do not know why intracellular pathogens had a lower decline. This could be due to their isolated niches, which limit opportunities for horizontal gene transfer (HGT) and genome rearrangements, as well as the smaller number of available sequenced genomes. Intracellular bacteria are known to undergo reductive evolution [23,24], a process where non-essential genes are lost while highly specialized genes are retained, resulting in greater genetic stability. In contrast, bacteria inhabiting diverse environments are more prone to gene acquisitions and losses via HGT, leading to frequent changes in their orphan gene status. Although the isolation of these pathogens may contribute to the observed trend, we argue that the limited availability of sequenced genomes is likely the primary factor, as even restricted genomes showed fewer orphan genes as more genome sequencing data became available.

**Fig 3:**
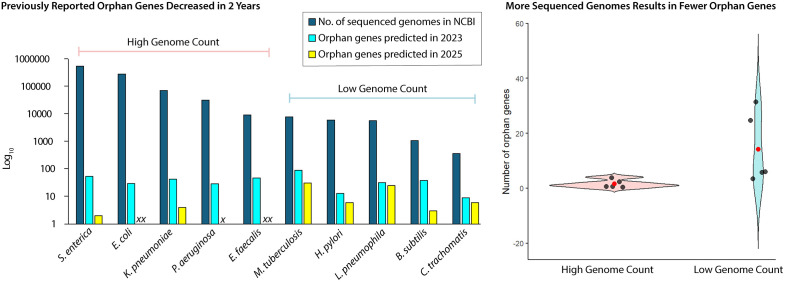
A Higher Number of Sequenced Genomes Generally Leads to a Reduction in Orphan Genes. The left panel demonstrates the number of orphan genes in 2023 and 2025, alongside the number of sequenced genomes available for each bacterial species in NCBI (S1 Table, individual data points without log transformation). Labels: *xx*: data points corresponding to a single orphan gene (Log 1 = 0); *x*: data point corresponding to zero orphan genes (Log 0 = undefined). The 10 bacterial species studied were divided into 2 groups and arranged in descending order based on the number of genomes available (NCBI). The right panel compares the top five High Genome Count species to the bottom five Low Genome Count species using a violin plot.

**General Trend: Increasing Genome Sequencing Reduces Orphan Genes.** We investigated how the number of available genomes in NCBI influences the count of orphan genes across bacterial species. Overall, species with a greater number of sequenced genomes tended to exhibit fewer candidate orphan genes. The 10 bacterial species analyzed were ranked in descending order based on the number of available sequenced genomes (NCBI) and then divided into two groups (Fig 3, Left). The top five species, with the highest number of sequenced genomes, had the fewest candidate orphan genes. However, the bottom five species, with fewer sequenced genomes, showed the highest number of candidate orphan genes (Fig 3, Right). This pattern suggests that the number of sequenced genomes strongly affects orphan gene identification, likely because increased sampling (more sequenced genomes) improves annotation accuracy and enables broader genomic comparisons, leading to the reclassification of genes previously labeled orphans.

Earth harbors immense microbial diversity with an estimated 10E12 microbial taxa [25], yet scientific attention is disproportionately focused on organisms of medical or industrial relevance. The majority of microbial taxa are uninvestigated. Indeed, half of all microbiology publications center on just 10 species (*E. coli* dominates the literature), and only 26% of known bacterial species have been studied, leaving over 90% vastly underinvestigated [26]. This uneven research landscape highlights the significant gap in scientific knowledge in microbial research and helps explain why poorly sampled species generate more apparent orphan genes.

While the number of sequenced genomes is a crucial factor, there are other factors that can contribute to the differences observed in the data. For example, *E. faecalis* and *M. tuberculosis* have similar numbers of genomes available in NCBI, yet they differ markedly in the number of candidate orphan genes identified in each species. We do not know why this is, but it could be due to the global prevalence and extent of genomic sampling. This is highlighted best by the World Health Organization (WHO) 2024 tuberculosis (TB) report. This report shows that much of the data on *M. tuberculosis* exhibits a limited geographical distribution, with high prevalence in specific regions. More than 66% of worldwide TB cases originated from just eight countries: India, Indonesia, China, Philippines, Pakistan, Nigeria, Bangladesh, and the Democratic Republic of the Congo [27]. However, *M. tuberculosis* is not confined to these regions, nor to human hosts. For instance, *M. tuberculosis* has been isolated from free‐ranging wildlife in South Africa [28] and from wild chimpanzee in West Africa [29]. The focus on human clinical isolates from select locales likely limits the detection of homologs in more diverse strains, thereby inflating the number of candidate orphan genes. In contrast, *E. faecalis*, a remarkably versatile organism, is commonly found as a commensal in the gastrointestinal tract of humans and animals [30], and is also frequently isolated from extra-enteric environments such as blood, urine, soil, water, plants, and a wide range of fermented foods (particularly in traditional dairy products) [31–34]. Furthermore, it can also be an opportunistic pathogen [30]. This widespread distribution promotes its inclusion in a broad range of studies, spanning microbiome research to clinical, environmental, and food-related research. Hence, comprehensive geographical and ecological sampling may be responsible for strengthening genome annotation and thereby leading to fewer candidate orphan gene identifications in *E. faecalis*.

Given the immense, underexplored microbial diversity, we predict two key trends:

1. New genes classified as orphans are likely to emerge over time.
2. As more genomes are sequenced, many genes once labeled as orphans will be reassigned, perpetuating a cycle of orphan designation and subsequent reclassification. To break this cycle, we must revise both our classification criteria and our terminology.

**Revising the Terminology for Orphan Genes with Descriptors That Better Reflect Current Understanding.** Our results demonstrate that genes once labeled as “orphan” (or taxonomically restricted) often lose their apparent uniqueness as more genomes are sequenced and annotated. Following the precedent established for newly proposed bacterial taxa, which are designated as “candidate species,” we suggest adopting similar provisional descriptors, such as “candidate” or “putative,” for newly identified genes lacking known homologs. These descriptors more accurately convey the provisional and potentially temporary nature of their apparent novelty. Using the term candidate is clearer and better reflects the underlying uncertainty when no homologs have yet been detected in nature. Another useful term is cryptogenic, a word commonly used in medicine to describe conditions of obscure or unknown origin, which is particularly apt as a descriptor for genes whose evolutionary origins remain uncertain. Regardless of whether the term is orphan, taxonomically restricted, cryptogenic, or some other term, we argue that a provisional descriptor is required.

The biological origins of these genes help clarify why a more flexible terminology is warranted. Such genes could have emerged through *de novo* gene birth (evolution from noncoding DNA) or through horizontal gene transfer (acquisition from another organism). While orphan genes have often been assumed to evolve unusually [35–37] rapidly, our proposed adjustment in terminology acknowledges an alternative explanation: their apparent uniqueness may stem not from accelerated evolution, but rather from insufficient sequence sampling. We also outline five additional reasons to employ descriptive qualifiers when referring to orphans, emphasizing the need for terminology that captures both uncertainty and the potential for future revisability.

1. Bioinformatic Artifacts: Many predicted genes might not be genuine but are artifacts of computational algorithms. The descriptor, such as candidate, emphasizes the provisional nature of these findings, recognizing that further evidence would be required to confirm their uniqueness.
2. Incomplete Genomic Sampling: Our understanding of genetic diversity is still tremendously inadequate. The genomes sequenced to date represent only a minute fraction of the total diversity on Earth. It is estimated that the Earth is home to approximately one trillion bacterial species. Fewer than one million bacterial genomes have been sequenced, with the majority belonging to the same species [38]. A recent study reported that, despite analyzing approximately 1.5 million microbial genomes, including Metagenome-Assembled Genomes and more than 18,000 metagenomic samples, a large fraction of prokaryotic diversity remains unsequenced, with about 42% of bacterial and 36% of archaeal phylogenetic diversity still lacking genomic representation [39]. Considering predictions of up to 1 trillion microbial species and that the NCBI database currently houses approximately 330,000 prokaryotic genomes, representing around 19,000 species, only roughly 0.000002% of prokaryotic genomes have been sequenced. Based on these figures, the unclear origin of many bacterial genes is primarily attributable to insufficient sequencing data, rather than the genes being truly unique.

In addition, bacterial phages (viruses), known reservoirs for gene transfer [40], are vastly underrepresented in the genome databases. Even though phages likely outnumber bacteria by a factor of ten or more [41], fewer than 5,000 phage genomes are cataloged in NCBI (a 2023 report) [42]. Moreover, phage genomes contain an exceptionally high proportion of novel genes with uncharacterized functions, indicating that much genetic diversity remains unexplored [41]. It has been argued that phages possess some of the greatest genetic novelty in the biological world, with as many as 80% of their encoded genes lacking similarity to known proteins and being of unknown function [41]. Thus, the challenge in tracing a gene’s origin is often more indicative of limited sequencing efforts than true genetic uniqueness.

1. Limited Domain Coverage: Our study focused solely on bacteria. However, similar issues exist for archaea and eukaryotes. For example, the Genome Taxonomy Database has 584,382 bacterial genomes versus 12,477 archaeal genomes (a 2024 report) [43], suggesting that orphan designations in archaea are likely due to under-sampling. In eukaryotes, approximately 41,000 genomes (from about 2,300 species) have been assembled (a 2024 report) [44], yet an estimated 8.7 million eukaryotic species exist worldwide [45]. This indicates that our current data represents only about 0.026% of eukaryotic genomes in nature. Thus, the inability to trace a gene’s origins in archaea and eukaryotes is often more a reflection of limited sequencing rather than true uniqueness.
2. Extinctions Lead to Limited Knowledge of Gene History: It is estimated that approximately 99.9% of all species that have ever lived on Earth are now extinct [46]. The vast majority of species went extinct before the advent of DNA sequencing. Tracing the evolutionary history of genes from extinct organisms is an extreme challenge. This gap in our data particularly affects genes that arise via *de novo* birth or horizontal gene transfer, since the donor species may be extinct. Thus, the inability to trace a gene’s origins is often more a reflection of the lack of sequenced genomes from extinct species rather than a true indication of uniqueness.
3. Implications for Evolutionary Interpretations: Overestimating *de novo* gene birth rates can mislead our understanding of gene evolution and, beyond science, fuel misconceptions about the origin of species. The assumption that orphan genes must have evolved extremely rapidly has even been used to argue for irreducible complexity, an argument often cited by creationists. Such conclusions rest on two flawed assumptions: first, that orphan genes will remain orphan indefinitely, and second, that any gene without a currently identifiable homolog must be unique. The limited scope of our sequencing data undermines both assumptions. Our study demonstrates that many genes previously classified as orphan genes do not retain that status indefinitely. We also showed that many genes, previously considered unique due to their apparent lack of homologs in external lineages, have since been shown to share homology, indicating they are not truly lineage-specific.

We initially retrieved 385 protein sequences from TRGdb for 10 bacterial species, and after completing all four BLASTp steps, 79 sequences remained (S1 File). However, four predicted proteins from *K. pneumoniae* were excluded from further analysis because the genome for this bacterial species was not fully assembled, which limited the ability to validate the presence of candidate orphan genes through this approach. As a result, genes from *K. pneumoniae* were not studied further. Of the 385 initial protein sequences, only 75 remained for further evaluation, representing an 81% reduction in potential orphan genes in just two years.

Overall, adopting provisional descriptors, such as candidates for newly identified genes lacking known homologs, would more accurately convey the provisional and potentially temporary nature of their apparent novelty. This change highlights the uncertainty in gene origin due to incomplete genomic data rather than inherent uniqueness. By adopting a change in terminology, we can foster clearer communication about what is known and what remains to be discovered in the evolution of genes.

**Prioritizing Candidate Orphan Genes for Experimental Studies**. Our analyses identified 75 candidate orphan genes, but the only definitive way to confirm whether these sequences represent true genes is through experimental validation. This next step focuses on prioritizing the most promising candidates for follow-up studies. While we highlight the strongest candidates here, it is important to note that those excluded from this subset may still encode functional genes; they were simply ranked lower based on the current computational evidence.

**Fig 4:**
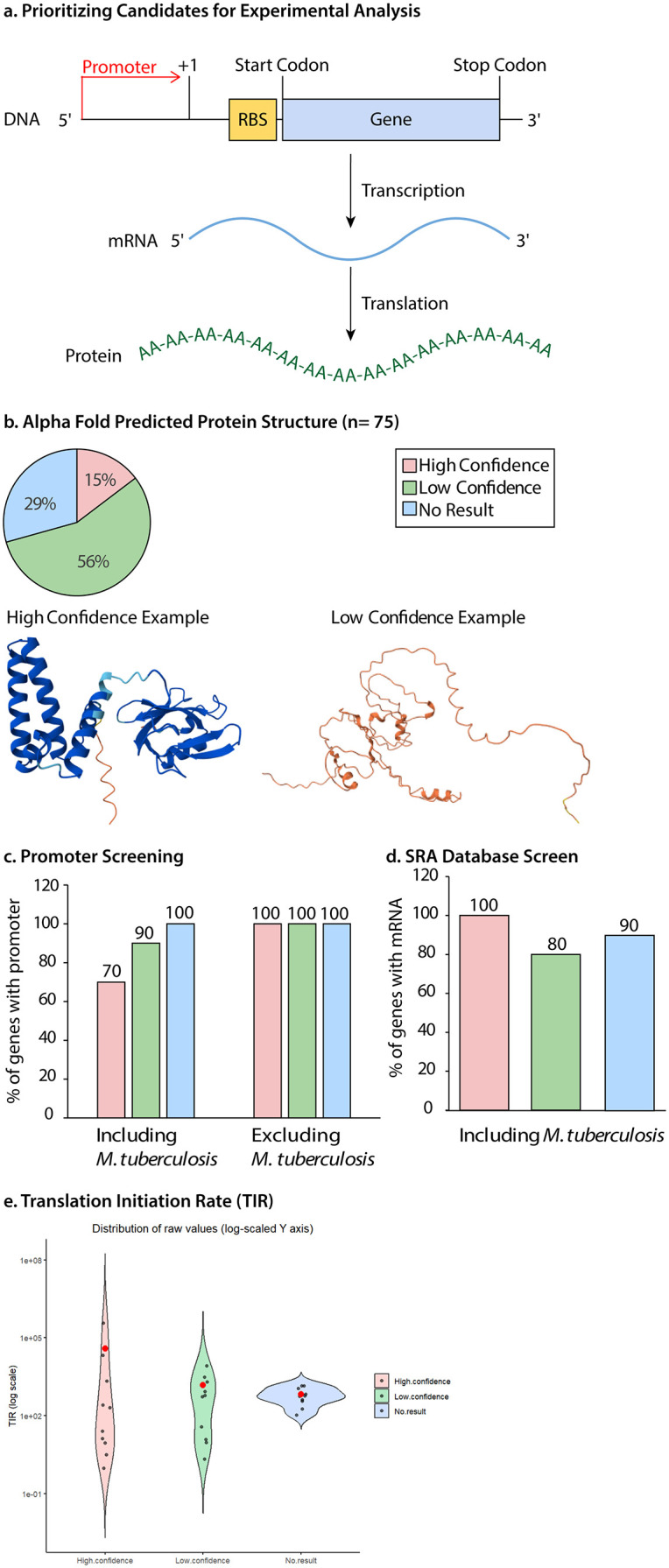
Prioritizing Candidate Orphan Genes for Experimental Validation. **a.** Workflow for prioritizing candidate orphan genes based on predicted protein structure, promoter elements, mRNA expression, and ribosome-binding site (RBS) strength. **b.** AlphaFold structural predictions for 75 candidate orphan proteins. Shown are representative models from each confidence tier: one with a high average predicted Local Distance Difference Test (pLDDT) score (90.56) and one with a low average pLDDT score (30.22). 10 proteins from each category (high confidence, low confidence, and no prediction) were selected for downstream analyses. **c.** Promoter motif search using BPROM and BioCyc. Excluding *Mycobacterium* species, every candidate orphan protein across all confidence tiers contained at least one predicted promoter site. **d.** mRNA expression profiling based on RNA-seq datasets retrieved from the SRA, confirming transcript presence (but not expression level) for selected candidate orphan genes. **e.** Translation Initiation Rate (TIR) estimates. High-confidence candidate orphan genes exhibited the highest mean TIR, while the no-prediction group showed the lowest average TIR.

In this analysis, we focused exclusively on protein-coding genes, as these are generally easier to assay experimentally than RNA-encoding genes (Fig 4a). AlphaFold [47,48] served as an initial filter to help identify candidate orphan genes whose protein sequences are capable of folding into stable structures. It provides an average confidence score referred to as pLDDT (predicted Local Distance Difference Test), which classifies predictions into four categories: very low (<50), low (50–70), high (70–90), and very high (>90) confidence. For our analysis, “very low” and “low” were grouped as low confidence, and “high” and “very high” as high confidence. The majority (56%) of candidate orphan protein sequences exhibited low structural confidence. Only 15% depicted high confidence (Fig 4b), while 29% of candidates did not produce any structural prediction (Fig 4b).

One reason for the high number of candidate orphan proteins with low confidence in structure could be correlated with the GC content. Codon usage is highly influenced by GC content in candidate orphan genes, which in turn plays a vital role in shaping the properties of proteins [49]. High GC genomes are likely to encode proteins that are enriched with amino acids such as alanine, proline, and glycine [49]. These amino acids tend to promote disorder, contributing to structural flexibility [50,51]. Consequently, candidate orphan proteins from genomes with a high GC proportion have often been identified as Intrinsically Disordered Proteins (IDPs). Such proteins do not possess a fixed three-dimensional (3D) structure in solution and exist in dynamic, flexible conformations, particularly in the absence of other proteins [52–54]. It has been revealed that low pLDDT scores might indicate the intrinsic disorder nature of proteins. Large-scale structural annotation of the human proteome using AlphaFold demonstrated that regions labeled as IDPs overlap with low-confidence predictions [55]. AlphaFold employs a structure-based algorithm that relies on sequence homology to known proteins; therefore, IDPs, due to their structural plasticity, are poorly predicted by such algorithms [55–57]. Although low-confidence AlphaFold predictions do not preclude candidate orphan genes from encoding real proteins, high-confidence models enable us to prioritize those candidates most likely to succeed in *in vitro* or *in vivo* experiments.

Following AlphaFold predictions (Fig 4b), 10 proteins from each category (high confidence, low confidence, and no result) were selected randomly to further investigate their likelihood of being functional genes (Fig 4c-e). This analysis included checking for the presence of start and stop codons, promoter prediction, assessing mRNA expression, and evaluating translation initiation efficiency by analyzing ribosomal binding sites. All the sequences had predicted start and stop codons; however, due to the high likelihood that these codons might occur by chance, we further examined the upstream sequence of the start codon for potential promoters using BPROM.

BPROM [58] predicted promoter regions for all candidates except those from *M*. *tuberculosis* (Fig 4c). This software is based on the Sigma 70 promoter motif, and according to the BPROM website, it has ~ 80% accuracy in predicting *E. coli* promoters. In contrast, *M*. *tuberculosis* does not rely on the classical Sigma 70 promoter but instead utilizes alternative sigma factors such as SigA, SigB, and SigE, which in turn recognize different promoter motifs. These motifs often diverge from the canonical −10 and −35 sequences targeted by BPROM [59–61]. Consequently, BPROM is not well-suited for identifying promoters in *M*. *tuberculosis*, so we also provided data where it is excluded (Fig 4c). We attempted to predict promoters for *M. tuberculosis* using BioCyc, but were only able to predict promoters for some of the *M*. *tuberculosis* genes. Consequently, promoter screening appears to have limited utility as a primary tool for *M. tuberculosis* promoters, which contribute to the majority of orphan proteins (of the 10 species we probed) and represent a special case due to their unique genomic and regulatory features.

To evaluate transcriptional activity of our candidate proteins, we queried the Sequence Read Archive (SRA) [62], a comprehensive repository of public RNA-seq datasets spanning diverse organisms and conditions. We used tBLASTn to detect matching mRNA reads for each protein. Quantifying read abundance by reconstructing RNA-seq reads across hundreds of millions of data points from various experiments and research groups is computationally challenging; therefore, we adopted a more qualitative approach. We examine the expression of each gene from three randomly selected SRA datasets to determine whether each gene exhibits detectable transcriptional evidence. Remarkably, all proteins with high-confidence structural models (100%) were supported by SRA reads, whereas 80% of low-confidence proteins and 90% of those lacking structural predictions showed evidence of transcription (Fig 4d). These results demonstrate that high structural confidence is strongly predictive of mRNA expression and thus provides a powerful criterion for prioritizing candidates for experimental validation.

To assess which candidate orphan genes had potential for high translational rates (protein production rate at the ribosome), we estimated them using the Ribosomal Binding Site (RBS) Calculator [63]. On average, proteins with high structural confidence exhibited the highest predicted Translation Initiation Rates (TIRs) (Fig 4e), suggesting that these proteins are more likely to be efficiently translated. These findings further support their potential for experimental validation. In summary, our integrated analysis, which combines structural prediction, transcriptional evidence from the SRA database via tBLASTn, and translation initiation efficiency, underscores the importance of considering both structural confidence and expression data when prioritizing candidate orphan genes for experimental validation. Proteins with high structural confidence not only demonstrate reliable expression but are also predicted to be more efficiently translated, making them prime candidates for further investigation.

Based on our findings, AlphaFold and the RBS calculator are particularly effective for identifying promising experimental candidates. Both tools are relatively fast, reliable, and can be easily automated. In contrast, the presence of start and stop codons was not informative because all gene-predicting programs are based on these features. Promoter prediction was less reliable for non-model organisms, such as *M. tuberculosis.* In addition, the distance a promoter is from a gene varies among organisms, making predictions often less useful, especially for non-model organisms, where promoters have been less studied. While SRA data can provide valuable expression data, analyzing it is time-consuming, making it less practical for large-scale screening. Furthermore, the SRA data alone provide a binary response (yes or no in the presence of RNA), without considering the quality or level of the RNA, without in-depth analysis.

Through this work, we identified 10 high-confidence candidate orphan genes for experimental validation and assessed their predicted subcellular localizations. Using bioinformatic tools, we classified 1 candidate as a membrane protein, 4 as soluble proteins, 3 as secreted proteins, and 2 as signal-peptide-only fragments (S2 Table). The occurrence of signal-peptide-only sequences is particularly intriguing, as it suggests these peptides may represent remnants of larger proteins from which downstream domains were lost through genetic mutation. Further *in silico* and experimental searches for additional homologs could test this hypothesis by uncovering the missing protein segments. Collectively, these findings imply that the 2 signal-peptide-only sequences may not constitute genes with functions, but rather vestiges of proteins that are no longer functional in these bacteria.

**Implications, Future Work, and Unanswered Questions**. This study utilized the TRGdb database, which was not constructed with all available genomes for each species at the time. As a result, our analysis was limited to individual genomes rather than considering all strains within a species. Developing an updated database that integrates a broader set of genomes, combined with the comparative framework established in this study, could further refine the estimated number of candidate orphan genes. Such an expansion would likely increase the detection of homologs present in additional strains or closely related taxa, thereby providing a more comprehensive and accurate view of orphan gene diversity.

A major challenge we encountered in this study was the frequent absence of clearly reported gene names or sequences in the literature. While many studies report the percentage of orphan genes identified, they often fail to provide a list of the specific genes (by name or gene ID), even in supplementary materials. In some instances, links to supporting data were nonfunctional, displaying messages such as “Page Not Found,” “This site can’t be reached,” or similar errors [3,64–67]. For this analysis, we relied on TRGdb [13], which provides a well-organized and searchable resource that facilitates gene identification and retrieval for downstream analyses. Nevertheless, the lack of detailed, gene-level information in previous studies remains a major barrier to reproducibility and comparative research, limiting the ability to validate reported findings.

Similarly, while analyzing bacterial promoters, we found that no single software tool was sufficient for accurate predictions across all species, requiring the use of multiple tools. This highlights the need for a more comprehensive bioinformatics tool capable of predicting promoter locations using genome sequences across a wide range of bacterial species.

Another significant challenge was analyzing gene expression data from the SRA. Although large-scale RNA-seq datasets are available, identifying accession numbers that match the exact strain used in our study proved difficult. Due to feasibility constraints, we limited our analysis to a small subset of accessions at a time. Additionally, our analysis focused solely on determining whether the candidate orphan gene sequences were transcriptionally active without taking into account expression levels, experimental conditions, false negatives in the data, contamination issues, or potential regulatory influences.

A further issue arises from the way BLAST/NCBI handles result ordering when excluding a specific organism. For example, in our *E. coli* screening pipeline, Step 1 requested the top 100 hits while excluding *E. coli*, yielding no hits from any other species. In contrast, Step 2 used the top 5,000 hits with *E. coli* excluded and did return matches from other organisms. This discrepancy occurs because BLAST first compiles all potential hits (the top were *E. coli* sequences), and only afterward removes those belonging to the excluded taxon. In Step 1, the first 100 results were entirely *E. coli*, so once those were filtered out, nothing remained. By expanding the search to 5,000 hits in Step 2, BLAST was able to gather enough non–*E. coli* matches to populate the filtered set.

While this workaround served our immediate needs, it has inherent limitations. As genomic databases continue to grow at an exponential pace, it will soon be common to see more than 5,000 high‐scoring *E. coli* sequences for a given gene. At that point, even requesting 5,000 hits will still capture only *E. coli*, and any more distantly related homologues, though present, will be excluded because they never enter the initial pool of top matches. In principle, one could raise the cutoff to 50,000 or beyond to ensure coverage of distant homologs, but NCBI currently caps BLAST results at 5,000 entries. To make candidate orphan gene identification and other evolutionary analyses more robust and accessible to the broader research community, BLAST/NCBI will need to either raise this 5,000‐hit ceiling or implement alternative filtering strategies that avoid this queueing limitation. A change needs to be implemented soon because the NCBI genomic repository is growing at an extraordinary pace. Alternatively, researchers may be able to circumvent this issue by using tools such as DIAMOND [68] or MMseqs2 [69], which support larger datasets and are not limited by this result.

In selecting candidate orphan genes for experimental validation, we prioritized proteins with high-confidence structural predictions from AlphaFold. These candidates also exhibited strong transcriptional evidence, including the presence of mRNA, promoters, and higher average Translation Initiation Rates. These indicators suggest that these genes are likely to be expressed and translated into functional proteins, making them suitable starting points for further characterization. However, it is essential to note that the absence of a confident AlphaFold prediction (or the other analysis we performed) does not necessarily imply that a gene is non-coding. Our strategy focuses on identifying candidates that are most likely to yield experimental validation; we do not exclude low-confidence or no-result candidates as legitimate protein-coding genes. The limitations we encountered in candidate selection mirror broader challenges in microbial genomics, particularly the uneven taxonomic representation of sequenced species.

A recent study determined that among the studied bacterial species, a disproportionate amount of research focuses on just 10 species, with more than 90% of bacteriology articles examining less than 1% of all species [26]. This limited representation affects our understanding of candidate orphan genes. In other words, as we sequence more genomes, we are likely to uncover additional orphan genes as genomic datasets from poorly studied or underrepresented species often do not contain sufficient annotation to recognize homologous genes, leading to an overestimation of orphan genes in earlier studies; however, with continued sequencing, genes once considered unique to a specific lineage may no longer be viewed as distinct.

We currently lack comprehensive experimental evidence confirming that candidate orphan genes represent genuine protein-coding sequences; however, our analysis has identified several promising candidates. Functional characterization of these genes would substantially advance the field, as experimental validation is essential to determine whether these sequences encode true proteins or merely reflect misannotated gene fragments.

## Conclusion

In this study, we reassessed orphan genes using a BLAST-based approach to determine their presence across bacterial genomes. Our findings indicate that the number of candidate orphan genes is influenced by the availability of sequenced genomes, with a general trend of decreasing candidate counts as more genomes are included. Many genes initially classified as orphans are simply lacking annotated homologs due to incomplete genomic data, rather than being truly unique to a species. However, the absence of enough genome sequences means that our estimates may still be inflated. Ultimately, experimental validation will be crucial in determining which candidate orphan genes encode functional proteins and in determining their biological significance.

## Methods

**Data Retrieval and Selection**. To investigate orphan genes in bacterial species, we obtained protein sequences from the Taxonomically Restricted Genes Database [13] (TRGdb) which contains taxonomically restricted genes from 80,789 bacterial species at the genus and species levels. We selected 10 bacterial species for this study based on their prevalence and frequent use in microbiology research and retrieved their corresponding orphan protein sequences (S2 File). For each species, a single genome was selected for analysis, even when multiple genomes were available. We selected either a fully assembled genome or the reference genome to ensure confidence in genome quality. In many cases, only one genome was available in TRGdb for a given species, which also facilitated consistency across species. We note that this choice is unbiased and should not substantially influence the results. The bacterial species and their respective genome accession numbers are listed in Table 1.

**Table 1:** A list of bacterial species and genome accession numbers used in this study.

Sr. no.	Bacterial Species	Genome Accession No.
1.	*Bacillus subtilis*	GCF_000009045
2.	*Chlamydia trachomatis*	GCF_000012125
3.	*Enterococcus faecalis*	GCF_000392875
4.	*Escherichia coli*	GCF_003697165
5.	*Helicobacter pylori*	GCF_001653475
6.	*Klebsiella pneumoniae*	GCF_000742135
7.	*Legionella pneumophila*	GCF_000008485
8.	*Mycobacterium tuberculosis*	GCF_000195955
9.	*Pseudomonas aeruginosa*	GCF_001457615
10.	*Salmonella enterica*	GCF_000006945

**Orphan Genes Screening Using BLASTp.** The screening of candidate orphan genes was conducted using a four-step BLASTp [14] approach, with an expected threshold of 1E-3 for all four steps, consistent with the TRGdb classification criteria for orphan genes. All BLASTp searches were performed using BLAST+ version 2.16.0. Steps 1 and 2 followed the BLASTp-based procedure described in TRGdb, which primarily focused on identifying orphan genes. In contrast, this study aimed not only to identify but also to analyze the potential function of these genes. Therefore, Steps 3 and 4 were newly introduced to enhance confidence in orphan gene selection. These additions ensured a robust set of high-confidence orphan gene candidates suitable for subsequent *in vitro* functional analyses. In Step 1, BLASTp was performed on the protein sequences of each bacterial species from TRGdb, excluding the target organism, with a maximum target sequence set to 100 (the default value). A protein would be classified as an orphan if it showed no significant similarity to any sequence in the database. Henceforth, protein sequences that exhibited no significant similarity in Step 1 were subjected to Step 2, where BLASTp was performed again. This time maximum target sequences were increased to 5,000. In Step 3, referred to as thresholding BLASTp, sequences that showed no significant similarity in Step 2 were reanalyzed, and the target organism was reintroduced while maintaining a maximum target sequence value of 5,000. Genes found in three or fewer genomes within the target organism’s species were classified as low-confidence orphan genes (excluded from further analysis), while those in more than three genomes were classified as high-confidence orphan genes. In Step 4, a reciprocal BLASTp search was conducted to identify potential hidden homologs for high-confidence orphan genes. The protein sequences with the lowest similarity scores from Step 3 results were used as queries against the complete BLASTp database. It also involved the inclusion of the target organism, with a maximum target sequence value set to 5,000. All other BLASTp parameters were set to their default values, and searches were conducted against the non-redundant (nr) protein database.

**Structural Analysis Using AlphaFold 3.** AlphaFold v3.0.1 [48] was then used to predict the structures of 75 proteins obtained in the end of BLAST analysis (S3 Table). For structure prediction, protein sequences were uploaded to the AlphaFold web server, which generated the predicted three-dimensional structures. The process begins with multiple sequence alignment (MSA), which is based on homology, followed by the prediction of protein structures. The confidence of each prediction is assessed using the predicted Local Distance Difference Test (pLDDT) score. The results were categorized into three groups: high-confidence (including very high and high pLDDT scores), low-confidence (low and very low pLDDT scores), and no result (for sequences without structural predictions).

**Screening for the Presence of Candidate Orphan Genes Using Custom tBLASTn.** After AlphaFold was used to predict the structures of the 75 proteins, we sought to confirm their actual preSsence in the respective genomes, as mentioned in TRGdb, before proceeding with further computational analyses. To do this, a custom tBLASTn analysis was performed using Geneious Prime 2024.0.7. This step ensured that the genes were truly present in the genomes of the selected bacterial species, thereby ruling out the possibility of potential annotation errors. The genome sequences of each bacterial species were obtained in “.gbff” and “.fasta” formats from NCBI and imported into Geneious bioinformatics software. A Custom tBLASTn search was conducted on ten proteins from each confidence category (high-confidence, low-confidence, and no result). Proteins that did not show 100% identity or 100% query coverage were excluded from further analysis. These proteins were replaced by other candidates from the same category that met the 100% identity and query coverage criteria. The custom tBLASTn step and the 100% identity/query coverage criteria were necessary. The protein sequences were obtained from TRGdb, but we could not rely solely on database annotations or software predictions. To ensure that each orphan gene is truly present in the selected bacterial genome, we performed a custom tBLASTn search of the proteins against the corresponding genome sequence. If a protein is genuinely encoded in the genome, the tBLASTn alignment should show 100% query coverage and 100% identity. This validation step was essential to confirm the presence of orphan genes before proceeding with downstream computational analyses and to provide confidence for future *in vitro* experiments. It also enabled precise mapping of each gene within the genome and retrieval of the corresponding nucleotide sequences (S4 Table).

**Functional Analysis of Candidate Orphan Genes.** To predict whether the candidate orphan genes encode functional proteins or are mere annotation artifacts, we proceeded with additional analyses on 10 randomly selected candidates from each confidence category (high-confidence, low-confidence, and no result) (S4 Table). For each candidate, we assessed the presence of key transcriptional and translational features, including predicted promoter regions, mRNA transcripts, and Ribosome Binding Sites (RBS). This multi-layered approach allowed us to better understand the potential expression and coding capacity of candidate orphan genes across different structural confidence levels.

**Promoter Region Detection Using BPROM.** The first step in assessing functionality was to check whether the genes were likely to be transcribed. We used BPROM [58], a tool that identifies potential promoter regions in bacterial genomes. We utilized a 100 bp upstream sequence from the start codon for each gene as an input sequence for BPROM that was retrieved from the genome. (S4 Table) and predicted the presence of a promoter. A confirmed promoter region suggests that the gene has the potential to be transcribed into mRNA. If no promoter was detected, we proceeded to the next step to investigate the possibility that the gene could be part of an operon.

**Operon Structure Analysis Using BioCyc.** If no promoter was detected, the next step was to explore whether the candidate orphan genes might be part of an operon. The presence of an operon is a common feature in prokaryotic genomes, where groups of genes are co-transcribed as a single mRNA molecule. We used BioCyc [70] to examine whether the gene could be part of an operon. It is a curated database that contains detailed information on metabolic pathways and operon structures. A gene within an operon may still be transcribed even without a clear promoter region.

**mRNA Expression Validation Using SRA Data.** To analyze whether the candidate orphan genes were actively expressed and not merely annotated without functional support, we checked for mRNA expression. This was accomplished using the NCBI Sequence Read Archive (SRA), a database that contains RNA sequencing datasets from various bacterial strains. For each selected gene, we performed tBLASTn searches against RNA-seq data corresponding to the strain in which the candidate orphan genes were identified. We used the SRA accession numbers (S4 Table) to search for RNA-seq data, ensuring that we used datasets from the correct strain. If no hits were obtained after testing three different SRA accessions, the gene was considered to have no detectable mRNA expression. This implies that it might not be actively transcribed or functional.

**Ribosome Binding Site (RBS) Analysis for Translation Potential.** To evaluate whether the candidate orphan genes had the potential for translation, we analyzed their Ribosome Binding Sites (RBS). The RBS is a critical component for initiating translation, as it guides the ribosome to the start codon. To estimate the translation initiation potential, we used the RBS Calculator v1.0 from Salis Lab [63]. We input the nucleotide sequence of the protein-coding region of the gene, along with 50 bp upstream, into the RBS Calculator. This tool estimates the strength of the RBS based on its thermodynamic properties. The calculator provides a total free energy value: lower free energy values indicate stronger ribosome binding and higher translation potential, while higher free energy values suggest weaker binding and reduced translation efficiency. This step helped us assess whether the candidate orphan genes were likely to be translated into functional proteins.

Through this detailed analysis that included promoter region detection, operon structure analysis, mRNA expression validation, and RBS evaluation, we were able to determine whether the candidate orphan genes were likely to encode functional proteins or if they were likely to be non-functional or annotation artifacts. This rigorous validation process ensured that the genes selected for further analysis had the potential to be biologically relevant and functional.

**Protein Property Prediction and Membrane Localization Analysis.** We performed *in silico* analysis employing bioinformatic tools to predict the solubility and association of proteins with the membrane (S2 Table) for 10 high-confidence candidate orphan genes. This step was designed to identify proteins that would favour downstream processing, such as expression and purification. Protein sequences corresponding to desired candidate orphan genes were analyzed using ProtParam [71], ProtScale [71] and DeepTMHMM [72]. ProtParam predicts certain physicochemical properties, including theoretical pI, instability index, grand average of hydropathicity (GRAVY), etc. GRAVY values estimate the overall hydrophobic or hydrophilic nature of a protein: positive values stipulate hydrophobicity, and negative values indicate hydrophilic nature. This provided us with the information related to solubility in aqueous environments. Then, the ProtScale tool from the ExPASy was utilised to assess the membrane association of proteins. A window size of 19 was applied, which is commonly used to detect transmembrane helices [71]. The Kyte & Doolittle hydrophobicity scale was selected, and the output predicts the hydrophobicity profile, where peaks exceeding a value of 1.6 suggest the presence of potential transmembrane segments. To predict the presence of the transmembrane region, we applied the DeepTMHMM tool. It classifies each region of protein as cytoplasmic, extracellular, signal peptide, or membrane-embedded.

## Supporting information

S1 Table Individual Data Points Without Log Transformation. This table presents the raw, untransformed data corresponding to Figure 3, showing the number of sequenced genomes available in NCBI for each microorganism alongside the predicted number of orphan genes in 2023 and 2025.
(XLSX)

S1 File Protein Sequences Retained After Step 4 BLASTp Filtering. This table lists the protein sequences obtained following Step 4 of the BLASTp filtering pipeline for each microorganism analyzed (*B. subtilis, C. trachomatis, E. faecalis, E. coli, H. pylori, K. pneumoniae, L. pneumophila, M. tuberculosis,* and *S. enterica*).
(DOCX)

S2 Table *In silico* Characterization of High-Confidence Candidate Orphan Genes. This table presents the outcome of *in-silico* analysis of protein sequences of ten candidates using ProtParam, ProtScale, and DeepTMHMM to predict physicochemical properties, solubility, and membrane association.
(XLSX)

S2 File Candidate Orphan Protein Sequences Retrieved from TRGdb. This table contains orphan protein sequences retrieved from TRGdb at the species level as the starting dataset for Step 1 of the analysis from 10 representative microorganisms.
(DOCX)

S3 Table AlphaFold3 Structural Predictions for Candidate Orphan Genes. This table lists all 75 candidate orphan genes identified after the four-step BLAST analysis pipeline. For each gene, the table provides the predicted protein structure using AlphaFold3, the species from which the gene was isolated, and the corresponding amino acid sequence for the candidate gene.
(DOCX)

S4 Table Analysis of 10 Candidate Orphan Proteins Per AlphaFold Confidence Category. This table lists 10 orphan proteins analyzed from each AlphaFold structural prediction confidence category (high, low, or no reliable structure). For each protein, it provides classification based on AlphaFold confidence, corresponding nucleotide sequence, 100 bp upstream of the start codon, and the SRA accession number used in analyses.
(XLSX)
