# Supplementary material for: Candidate orphan genes: Reassessing uniqueness
Source: PLoS One. 2025 Dec 31;20(12):e0338891. doi: 10.1371/journal.pone.0338891 (PMC12755737; doi:10.1371/journal.pone.0338891)
Supplement: S1 File — This table lists the protein sequences obtained following Step 4 of the BLASTp filtering pipeline for each microorganism analyzed (B. subtilis, C. trachomatis, E. faecalis, E. coli, H. pylori, K. pneumoniae, L. pneumophila, M. tuberculosis, and S. enterica). (DOCX) [file pone.0338891.s002.docx]

Bacillus subtilis

>NC_000964.3_524 genome:GCF_000009045 level:species cluster_id:NC_000964.3_524

MNGRDNYLSLLLLYGVLVALSSVNFGLTVFFLFSSPLHIINKHEKDFNENKMISVCISNFQNE*

>NC_000964.3_1946 genome:GCF_000009045 level:species cluster_id:NC_000964.3_1946

MLYLIGDHIERNTLLKTREKGAKKLLYKFKQLLYK*

>NC_000964.3_2243 genome:GCF_000009045 level:species cluster_id:NC_000964.3_2243

MILTQMKLFMQSLNGGYKIDLYNRELQRALNLHFWWICKVIYLVKEIKE*

Chlamydia trachomatis

>NC_007429.1_122 genome:GCF_000012125 level:species cluster_id:NC_007429.1_122

MAIIFGELRVSLYPPFLFTYISRQAYPKAILHCADKNFLESLHSNQCFRIANATCSTLSYAEIDR*

>NC_007429.1_162 genome:GCF_000012125 level:species cluster_id:NC_007429.1_162

MTEQPHRLSEEARSIANEAKERLLKLNDKPELSRPYLVKGFLSKISRGISSKEAASKFSTAIVTSLLGYL

WNHILQNKERILSADARDLEARSVLLDTKEILALCKYFAINRCTWLSSSSTQEEENLICQDIAKEMFSIS

LHALVEQQWIREYDLSVNNGSSYKLFF*

>NC_007429.1_168 genome:GCF_000012125 level:species cluster_id:NC_007429.1_168

MTFFSLFNRPLTVDFCSGVIPSFSSMFLGVRSYLLASRFSIFPEEASSFFSSIARFFLDKTAAVGGTALA

EIVFTPSSEQSTYERSLAV*

>NC_007429.1_296 genome:GCF_000012125 level:species cluster_id:NC_007429.1_296

MLAKKQDQKNLFLKPPLLNLNYKKTNQFNFSKKELNINCCKKQYLF*

>NC_007429.1_372 genome:GCF_000012125 level:species cluster_id:NC_007429.1_372

MGLEKGAIYSRSCEERAEIGKQRKIFACIKKSFHLDWRKFFWANLHGDRFQKASRHIYIMVRLLFSFCSY

PYLGKTSEEVEGCL*

>NC_007429.1_848 genome:GCF_000012125 level:species cluster_id:NC_007429.1_848

MRAFCKQIEMMKARLFASCFSPYSIDWIVFRNEKDSPINMKKQGEEDVYAE*

Enterococcus faecalis

>NZ_KB944590.1_866 genome:GCF_000392875 level:species cluster_id:NZ_KB944590.1_866

MDNLVIMKNQQAVTTSLQVEQRLIKIIVMF*

Escherichia coli

>NZ_CP033092.2_3836 genome:GCF_003697165 level:species cluster_id:NZ_CP033092.2_3836

MITGCGNDKDKFQCEAYLYPHPLIILDLEEHFTIQG*

Helicobacter pylori

>NZ_CP011487.1_433 genome:GCF_001653475 level:species cluster_id:NZ_CP011487.1_433

MSLGKGLISLIKDDTIPIETRLHENKLTIISKTDNIEIQNIEFNRGNCSDTAYNKGSERIEKESEEELAR

EYFYYELESDRNFIAKNEKTI*

>NZ_CP011487.1_555 genome:GCF_001653475 level:species cluster_id:NZ_CP011487.1_555

MKDYEDELEDFGEEELEGFEEEYEEYGDYKNVYDDDDYEDFNSDYEEE*

>NZ_CP011487.1_776 genome:GCF_001653475 level:species cluster_id:NZ_CP011487.1_776

MIDKVFTALNFLLASKETLEQQDKELKEKHLYPSDKNNCNTNNQTTAIPTKICLFTAKILKNQ*

>NZ_CP011487.1_779 genome:GCF_001653475 level:species cluster_id:NZ_CP011487.1_779

MLDVENEQKISVQLTKFSLKNICLGQRQD*

>NZ_CP011487.1_1453 genome:GCF_001653475 level:species cluster_id:NZ_CP011487.1_1453

MIEITFSGINDSNEFVSHGRFVQFTDKQGLGCPLHKLTKKGN*

>NZ_CP011487.1_1464 genome:GCF_001653475 level:species cluster_id:NZ_CP011487.1_1464

MAHHEQQQQANSQHHHHHHAHHHHYYGGEHHHHNVQQHAEQQAEQQAQQQQQQKAQQQNQQY*

Klebsiella pneumoniae

>NZ_KN046818.1_724 genome:GCF_000742135 level:species cluster_id:NZ_KN046818.1_724

MQPFKNFLYQTGLNISIIFVKQAIQVIISIDRIIFLSFINFFC*

>NZ_KN046819.1_27 genome:GCF_000742135 level:species cluster_id:NZ_KN046819.1_27

MWSNYYISHSSRISQFTTIATSHIDNYVIKLFIELTQRLADVSIFLNKGYAR*

>NZ_KN046820.1_46 genome:GCF_000742135 level:species cluster_id:NZ_KN046820.1_46

MSAPYASMLRHAVNIFTSFPVNTGNGIMGMQAFLMLNPDIKCFTKGCTTKNIIEPTGSIFAKHQILTVSQ

RKSLFVAPLDDRLHGCPESNFSIINQLKHRQ*

>NZ_KN046820.1_92 genome:GCF_000742135 level:species cluster_id:NZ_KN046820.1_92

MVAYQDRLSQRDSRCQRINMRKRLLRFNRPPVTVVTEPPGALLLAIKQGAVLVLDITGEGKQDILPPRRP

GWRHKEFLFGRPAKACFVDQGIRFFIESQ*

Legionella pneumophila

>NC_002942.5_21 genome:GCF_000008485 level:species cluster_id:NC_002942.5_21

MARDEPQEITAVCWSKNLPGAAFAPLNNTPGCINTYLTKSGLNLIDIGI*

>NC_002942.5_70 genome:GCF_000008485 level:species cluster_id:NC_002942.5_70

MDELKKRAYLALTNISRKVILTLLIELRSNYEQFEK*

>NC_002942.5_457 genome:GCF_000008485 level:species cluster_id:NC_002942.5_457

MFLTKKLGVFLSPEKIKLEGNPLECYKSSSPQIFKLLALAKSIIA*

>NC_002942.5_1063 genome:GCF_000008485 level:species cluster_id:NC_002942.5_1063

MTKLGLGSASSPRMLFVRAIQPQTWIVPCMPFWLSLISFFSSSLLSMFISSSLTLTIPISLALFRMKLPD

SCLPISKSTTFTCVNFSTLSFELRTPSLPMTHVELGYYERNHRYSNSPVTKPINAFRSLTTRTIT*

>NC_002942.5_1163 genome:GCF_000008485 level:species cluster_id:NC_002942.5_1163

MPSRSLASDLMFFCQGFETMLMYLLSPNVTWGSTGFYILFIYNSRYLVDRFRSCLFCRHELFNKKLFNHA

LL*

>NC_002942.5_1251 genome:GCF_000008485 level:species cluster_id:NC_002942.5_1251

MKFLLAQLFHYLELLNVKSLVQKHLGLDI*

>NC_002942.5_1323 genome:GCF_000008485 level:species cluster_id:NC_002942.5_1323

MQDDLKYYFWAGPDLIAVAILLLEVTNERQKSGVGLDCSLQVFSIRGHMMEMVAMGGLEPPTSAL*

>NC_002942.5_1603 genome:GCF_000008485 level:species cluster_id:NC_002942.5_1603

MNYQLELVADLVMPLINLSLNLVWSCLKVTKIVYTHSDKKNGFCPE*

>NC_002942.5_1923 genome:GCF_000008485 level:species cluster_id:NC_002942.5_1923

MIYYCIFEINPTIIGLINKLNRNLRLSLL*

>NC_002942.5_1984 genome:GCF_000008485 level:species cluster_id:NC_002942.5_1984

MSLNLKNIELFLETNDLQCYPGFIVTGLVNPNSQIKNCKDRTYAPTSTVPSAIMHVPAEEANNDPRSSYG

QQSK*

>NC_002942.5_2149 genome:GCF_000008485 level:species cluster_id:NC_002942.5_2149

MELIAGTEAGGFLLLLLKLKPENEEVYSVL*

>NC_002942.5_2238 genome:GCF_000008485 level:species cluster_id:NC_002942.5_2238

MANTRTSRFFYKLDFFEGLHAPFRNRTGLTKNSKVEAKNVFNEGA*

>NC_002942.5_2306 genome:GCF_000008485 level:species cluster_id:NC_002942.5_2306

MPYYLLTKIFDELFKFFRELSLREVIASFLYHFTKSFGFTQIKNYPGPFSILKRPGSYDR*

>NC_002942.5_2400 genome:GCF_000008485 level:species cluster_id:NC_002942.5_2400

MTLPKKLWSSASLDSSGAGYKDIILVEGYFRF*

>NC_002942.5_2410 genome:GCF_000008485 level:species cluster_id:NC_002942.5_2410

MLHDILIVNAHESMLKNSPLSYNLSKLLRIWFPSQVALPLA*

>NC_002942.5_2416 genome:GCF_000008485 level:species cluster_id:NC_002942.5_2416

MKRKRHLLSYQEFSINTLSVTTSILLVAQDFYKVLNKIN*

>NC_002942.5_2548 genome:GCF_000008485 level:species cluster_id:NC_002942.5_2548

MRRVILVQQFSIPYHVVRDNNGKDKQLELYIYKSY*

>NC_002942.5_2553 genome:GCF_000008485 level:species cluster_id:NC_002942.5_2553

MYRLPIFYLSEIGLSCESIQNEMVQNEGLAHLKVSEKFGSVRKATPFVCGE*

>NC_002942.5_2567 genome:GCF_000008485 level:species cluster_id:NC_002942.5_2567

MTQSRFVKDVISLDEFSCKLTIIVNLNRIAI*

>NC_002942.5_2575 genome:GCF_000008485 level:species cluster_id:NC_002942.5_2575

MHCKTHHAYKRLGRPTSELRIIIYLYIYFCLFEKIFFKSDPQKNL*

>NC_002942.5_2735 genome:GCF_000008485 level:species cluster_id:NC_002942.5_2735

MRKAQGDDEKASWDAKYGAFQMLISWEVILISPST*

>NC_002942.5_2926 genome:GCF_000008485 level:species cluster_id:NC_002942.5_2926

MRNKDEKAKEVATDSRCAEANRLTQLITGAILADNCFFPSGVYSIKPMVSSPQPPQEKTTPSM*

>NC_002942.5_2965 genome:GCF_000008485 level:species cluster_id:NC_002942.5_2965

MELATTGNEFISFAVAVVTFLAGWKLSSTNISSYFLS*

>NC_002942.5_2969 genome:GCF_000008485 level:species cluster_id:NC_002942.5_2969

MQIILAVYICDILLINAGILRFYGYFSFFTLYLLWALVIFVKTEKHYLLIEIKKRISICFTKMAKIQSVF

K*

>NC_002942.5_2994 genome:GCF_000008485 level:species cluster_id:NC_002942.5_2994

MKICLFICHDDELEIIILIKHSGLSYIAQSNKGLLTEAL*

Mycobacterium tuberculosis

>NC_000962.3_3002 genome:GCF_000195955 level:species cluster_id:NC_000962.3_3002

MWTSAGPADRHSWRPKPGPTSPPSADRRRPGTRGARPPARRSRWAPTPADRPGPGRLPARSWCVAHPGGP

VKPPAVPRAARRIRPSRCRPRKPADRGARPGRHRGAPDSNWAMRRRTAGLPPAAAAAASPAGSRRHGPAP

HQAATSKGSVRCGSEPSVPLQCSAARAIWPIAAARFVDNVVAVDSAAGKITSWVGVNYSAQLASAG*

>NC_000962.3_3244 genome:GCF_000195955 level:species cluster_id:NC_000962.3_3244

MHTSLRVTGVLPDRLLNQRFDVAGVEVDQPGGQPVTVVFAEGGCELAGEVVDVLASVVEVHDRGGFGQDR

GGQVPDPGGAIPRPQVRQW*

>NC_000962.3_3317 genome:GCF_000195955 level:species cluster_id:NC_000962.3_3317

MWGEAGFEGTTTRIREPTSTREQTQKSPISGEIGDFCVCSPRAPRLTTRRR*

>NC_000962.3_3359 genome:GCF_000195955 level:species cluster_id:NC_000962.3_3359

MLAMIALIKVIRSGGATVTAQCRLPAPQYPVPAQGRHIDHGPQPLALTERGDAADHVAGGLFGGSGFSHG

RFGHP*

>NC_000962.3_3523 genome:GCF_000195955 level:species cluster_id:NC_000962.3_3523

MQCDGQLYHAKSRAEMATGSRHCFYEPPTHLHGQLWQLAFGQCLHIARSADRGN*

>NC_000962.3_3662 genome:GCF_000195955 level:species cluster_id:NC_000962.3_3662

MAPRLGDTAGSPFVVKSGWTTRRRTPGAPSRPLGPSRLDAAAAALAARSAVAAGAAGATGWCLTTVSGAT

AAAGVSLAAIPAGAAVPTTTAGAAIAAGAARAGATAVAAGATLAAGGAGAASTTGSRGHSGLGVAAIAAG

AADTAGPTGAAVAIASRAGVAAVAAIAGRTAAAAGAAVAAGSADPGVAEAAGAAGAAGAAGAPVPAAGAA

GAAIAAIAGRTAGAAGAADLAALAAGTAVAAVAAIAASAAAKAADARAAGAAGAAVAAGAPVAAGVVSEW

AVAADGPVAAIAAGAALPAGAVGASGQATAAGPGPAAEATVAAVATVTARLRSHVCGVICSVAAVTAVTA

GATAAAGAASTGEAAVAAGAARARRAAVPAGTAVAAAGAAVAAHAGVAAVAAAAAVAAVAAARPGTAVAA

HAGDAAVAAVAAVAGAVAVAAVAGVAGVAASAAVAAGAAAAAPSAAGATVSTTTTVAASAAGAIAGITVA

AVTAIATCAGVAAVAAAAAVGSTLPAGPTTASVTALSTGAGGDRATADGAVAAGAAGTAVAAGATLTPGP

ATGAALAAGATLSGGAAVTGGPRGARGAAGDIAAVAAGPAGTAGTTLTTGTAAGAALATLSAVAGRPAGA

AGASGAAGAAAAAVASGAAGAAAAAVGS*

>NC_000962.3_3665 genome:GCF_000195955 level:species cluster_id:NC_000962.3_3665

MWRSGVRKANRCDPDQPGDYLVRLMPSDRPSAAVDAYTVGSTDTLGWLTEWPPQLPDRTVSGNMSPASSP

SPGDRPAPQADVFSCPPWAARPTPVSGCPRRTPAGLADGYPRECRTMAVDQVRSGFGEPSAAISRRARTP

VPYFHPHRCRLGPREARQPTHVDLPCGVSGAGRSRTHHPPLVTCPACTSSAGRASVCSLAQR*

>NC_000962.3_3822 genome:GCF_000195955 level:species cluster_id:NC_000962.3_3822

MCIRSLGMECRRRWITCIDVKPGESPIHRRLGLALGHGMPGRCLSPISIAPSSHPGMASPIRSLERLDTQ

ITCSVGTANRLAYL*

>NC_000962.3_3856 genome:GCF_000195955 level:species cluster_id:NC_000962.3_3856

MVVSRADTQPARSQLLHKGRIVALLFAKASCDT*

>NC_000962.3_4005 genome:GCF_000195955 level:species cluster_id:NC_000962.3_4005

MVEMLGVCVVATDLDGHRIVLRDSEKTLKMRNR*

>NC_000962.3_4065 genome:GCF_000195955 level:species cluster_id:NC_000962.3_4065

MRYVTGDHAVQVFQLTSTVIDLTTKRKHTTVVYAATSMSGTPPLHR*

>NC_000962.3_1657 genome:GCF_000195955 level:species cluster_id:NC_000962.3_1657

MLAQFPGGDQRAQPGQQPVVVALGLAAGVAVDGGRIGGGVDDRGVVVGVGRAGGGQFG*

>NC_000962.3_1780 genome:GCF_000195955 level:species cluster_id:NC_000962.3_1780

MSPWCIDYVPSYFVGVASAFALPACAFTTIDPVADMYRDGVSPEPGRQTLPTVTIIPLWGINTRVTRPKP

FSQKISYGD*

>NC_000962.3_1795 genome:GCF_000195955 level:species cluster_id:NC_000962.3_1795

MTEALCDKLVGAWDLVSYVERAAALALGYLAYGGR*

>NC_000962.3_1810 genome:GCF_000195955 level:species cluster_id:NC_000962.3_1810

MYTRFDLPSQDGATAFGQILILLLRRTASAR*

>NC_000962.3_1849 genome:GCF_000195955 level:species cluster_id:NC_000962.3_1849

MLKWTRPTRLTRQMRGGPVAQFKAREEELP*

>NC_000962.3_1981 genome:GCF_000195955 level:species cluster_id:NC_000962.3_1981

MPAASVGRAEPEAPVAGVVPAVAAGRAVEAEMAA*

>NC_000962.3_1995 genome:GCF_000195955 level:species cluster_id:NC_000962.3_1995

MAGGRRHHDEICCGVAAAGVSDGDGAVPEIDGCIVDPVLRWFQGRDRAVNLSCVRAVAQGIFAEPVGSRP

*

>NC_000962.3_2029 genome:GCF_000195955 level:species cluster_id:NC_000962.3_2029

MKDADDLADYGLSIEQVRAAVDSHVDVDHSVSAL*

>NC_000962.3_2069 genome:GCF_000195955 level:species cluster_id:NC_000962.3_2069

MPKDRLPDLTPTGAYAPANSGMTMARQDGPR*

>NC_000962.3_2153 genome:GCF_000195955 level:species cluster_id:NC_000962.3_2153

MFGNCDCVGAAAELGGVRSIVPGIGRVEVRQPVGDRCRDRLGDIGVVEDVFVGRVVVAHGAEQRVGIDRL

DTRMAGQHLVDPRVVPAAVVNHQLRVDDRGGVRGAGLVRMRVGMGAGEDGFDGYMPAGDRACHAAPHIPF

PFARPARPPDHRNDNHFQYNLVAAEDRAPWKTTGVVVGMDGGFHGCPVSSSLFSAVTPWAGIGSPHGSWC

H*

>NC_000962.3_2307 genome:GCF_000195955 level:species cluster_id:NC_000962.3_2307

MLANGRRKSSHPVYTLVLSGRTEPIKPAKG*

>NC_000962.3_2410 genome:GCF_000195955 level:species cluster_id:NC_000962.3_2410

MTARPRHYAKPSAARSHNQRAMIFRPWKKSLLARQRWDTGHVGAKRLHSPHE*

>NC_000962.3_2413 genome:GCF_000195955 level:species cluster_id:NC_000962.3_2413

MEFAPGAVDTIASDNMAAQNVHDTAVKTSR*

>NC_000962.3_2923 genome:GCF_000195955 level:species cluster_id:NC_000962.3_2923

MDPGELQMVREVVRPKTPRGDGNWIALTGLALILVVVRPKTPRGDGNLERVTADGTIETSRQTQNPERGR

KPSAQHR*

>NC_000962.3_7 genome:GCF_000195955 level:species cluster_id:NC_000962.3_7

MRMAWSTVGAHIGQRPGQAAYQMLETRRRGSVLRLGNPKRGIVSRRRYHTLRGARPTRPPPPMLG*

>NC_000962.3_353 genome:GCF_000195955 level:species cluster_id:NC_000962.3_353

MVTTCWSCWSATDFGGLLSVIMEPPGSPSWMSSPPDQPLPNSPPLAPLAISGPQQRLGRRIGGGLGAGTR

ARTHVQVLCKLAMERRRLIVEGVMPPTVCGNQCRDGRRHLVFGRRQHAGLVALRREVADPRCWRLVSLWW

PGPGGGAQWARWGRGRPGKNAAPWPGHERSRQARRRPACAVQEPHGPHRVPCSPSAATEPVSP*

>NC_000962.3_475 genome:GCF_000195955 level:species cluster_id:NC_000962.3_475

MDTQPATCRCGTGPKSGLKLTGVKARSFATNPEMLSTIRGTARWAHQPPFGGLILATSYEIVISITHGIR

RPIAYH*

>NC_000962.3_734 genome:GCF_000195955 level:species cluster_id:NC_000962.3_734

MRAAGPGSAARTCRTRWLPRRAYCWPTGGGELPTPAIWCGWMARSVGLRGRRRRARRSALRSGIHLRSRE

YSGRLGCRRCLSGRGGRTRSPSKRLRQTGLRRRQAGLVIPASAATVWSLIVRWVIVVSICSRRRTRRSGA

RWGQYRSAREARRWRRVGRVIVASAVGRRFCRPTVPTWLGPIRGAVWQSAGPLSASCSSSSISSRLNPWD

YARLTDETTDTASARYRDSPENDRAGAASRSSARNSAASGR*

>NC_000962.3_1117 genome:GCF_000195955 level:species cluster_id:NC_000962.3_1117

MQSVRQIFPQDIFIGRTMGVVVERYGSTDLSSRGMFVPFHDVDCVQ*

>NC_000962.3_1165 genome:GCF_000195955 level:species cluster_id:NC_000962.3_1165

MADEPRLEAGAHPFEEGRDKAPELRATQMDHVRFTEGRRERNRDRLERSQQFRQPGR*

Salmonella enterica

>NC_003197.2_2372 genome:GCF_000006945 level:species cluster_id:NC_003197.2_2372

MKFSSCQIVGQEYIYYARELDAFLPYIEVKK*

>NC_003197.2_3814 genome:GCF_000006945 level:species cluster_id:NC_003197.2_3814

MTVICLDYDGLMTMEIKKAGPGRHQTSLQGEMN*
