# Supplementary material for: Candidate orphan genes: Reassessing uniqueness
Source: PLoS One. 2025 Dec 31;20(12):e0338891. doi: 10.1371/journal.pone.0338891 (PMC12755737; doi:10.1371/journal.pone.0338891)
Supplement: S2 File — This table contains orphan protein sequences retrieved from TRGdb at the species level as the starting dataset for Step 1 of the analysis from 10 representative microorganisms. (DOCX) [file pone.0338891.s004.docx]

Bacillus subtilis

>NC_000964.3_166 genome:GCF_000009045 level:species cluster_id:NC_000964.3_166

MNEIWAFLYNIHLSSIDIIERREILNRGLAYDASEAEPCIGESDAAVNAD*

>NC_000964.3_343 genome:GCF_000009045 level:species cluster_id:NC_000964.3_343

MNARRTKETHVKVKDAADPSLIYANTGRNGQEA*

>NC_000964.3_344 genome:GCF_000009045 level:species cluster_id:NC_000964.3_344

MKPIFRINLSLADAPKAYDIFDEKENGNI*

>NC_000964.3_409 genome:GCF_000009045 level:species cluster_id:NC_000964.3_409

MAAPACPFPYENFHESLTKQIQMLYNNLEFLGKHRVKEVECKNNHVKEKKKNQNDRCTQYHYDRSSHFYD

QIGVLQAFSY*

>NC_000964.3_486 genome:GCF_000009045 level:species cluster_id:NC_000964.3_486

MWNYEYTAIADVDVGKPGTVIRKQKQWINGRRVLHQIL*

>NC_000964.3_524 genome:GCF_000009045 level:species cluster_id:NC_000964.3_524

MNGRDNYLSLLLLYGVLVALSSVNFGLTVFFLFSSPLHIINKHEKDFNENKMISVCISNFQNE*

>NC_000964.3_683 genome:GCF_000009045 level:species cluster_id:NC_000964.3_683

MCAPADTGLTMQVLPYALFFYEKRIKTPFSVNNEINGFFRFSALCEFVIMFKW*

>NC_000964.3_878 genome:GCF_000009045 level:species cluster_id:NC_000964.3_878

MISLFLFAAHLHEFLKKKSLNSLEKSFKMKLVVF*

>NC_000964.3_1333 genome:GCF_000009045 level:species cluster_id:NC_000964.3_1333

MEAIEEILLLYAKERYFFSFLHVYVGCRANERSVFVELQAESVEQG*

>NC_000964.3_1354 genome:GCF_000009045 level:species cluster_id:NC_000964.3_1354

MFFGVGHAGRAQIYDKTSEKRLKHAFKPKVTDEAGGWHGVCRAELRVYAVLYLHRA*

>NC_000964.3_1761 genome:GCF_000009045 level:species cluster_id:NC_000964.3_1761

MWERWFDRPADIVQMSNLIYQPLYVTVYKVIQLETVSIERLNL*

>NC_000964.3_1799 genome:GCF_000009045 level:species cluster_id:NC_000964.3_1799

MWKALSQLLKKQKNQSPSDEDYIQIPELEVKVLGMLHSINIDLVNVIAQAEKSKEFIGQIEGIWHSIANQ

FYSLAQGFENEDINKLSADLDHAAATWEAVANKAKEFVTNSYQG*

>NC_000964.3_1842 genome:GCF_000009045 level:species cluster_id:NC_000964.3_1842

MFGEGFYPTCSTMPYLLNCTSDWVKVKLKYTLHYILYEESELFYQMLN*

>NC_000964.3_1946 genome:GCF_000009045 level:species cluster_id:NC_000964.3_1946

MLYLIGDHIERNTLLKTREKGAKKLLYKFKQLLYK*

>NC_000964.3_1951 genome:GCF_000009045 level:species cluster_id:NC_000964.3_1951

MSLLPERSTDKNSFVFLLYLYKGIETVARNGEPYTVLNDLLFAAGNLHIGLGHDYLKCIALLINVKRFFS

LTESYGNISDKLAVVAMKGVQFDQFEEAYKAYKETVAINQNSLVDFLSALITLIKAGEKLQKAGESLAII

MNKLLEMSPENSMK*

>NC_000964.3_2049 genome:GCF_000009045 level:species cluster_id:NC_000964.3_2049

MLDAEISKEPSLLGRLFLCEKVFAGNVMPPFGHSYNMVKGVLRWEHEQLFD*

>NC_000964.3_2063 genome:GCF_000009045 level:species cluster_id:NC_000964.3_2063

MIEIFKDTGATHDLVYHSKINTFVWDVEFDIVLSDSKELNKCYFVKCFNPYRINGKCDFAVSSIDIFSEG

KRLLIENEFNFKITKAVHVATSKDVTEIVLHLSERISSPFPIVKEVVYLD*

>NC_000964.3_2065 genome:GCF_000009045 level:species cluster_id:NC_000964.3_2065

MKEIEFEEVKKFVEVMSRRLRSTKVIKIHISYAEWLVAEVERLRGLVK*

>NC_000964.3_2066 genome:GCF_000009045 level:species cluster_id:NC_000964.3_2066

MDSREQIDWTCNECNFSWIGDNSDFSCPSCDEIDIKPKNKILD*

>NC_000964.3_2085 genome:GCF_000009045 level:species cluster_id:NC_000964.3_2085

MIQGFYKDQKLHLLEDPMQQYTVMKVEENAVCVYRWIDDYRHKIERFTDVEEAKKLLGEGWPKQ*

>NC_000964.3_2088 genome:GCF_000009045 level:species cluster_id:NC_000964.3_2088

MILKEIAVYEETLNKHLPNETRMVLHYKNVEATENEDKITVRISNDLLVYKWVELGGVSKGTYKMKMTSL

EDETSEVNVKIERIQMNSDRDLAVEIEFNVLNPFQSVVGVPGIHSFSIKG*

>NC_000964.3_2089 genome:GCF_000009045 level:species cluster_id:NC_000964.3_2089

MSDDNAAKIIFKKKSTGKIVGEMKATSLAADTILQNDNKSK*

>NC_000964.3_2090 genome:GCF_000009045 level:species cluster_id:NC_000964.3_2090

MNKDTKDIWSGFFMGSGSLIVVGLLIFVEALTMSLIVYYGLNHVLNPLLIDTYNIQNVHVTLPHAFVIGV

LLNVFVKGVKRSDQEKDENIFKKAGKSLFHSTFALIVLYVSTLFI*

>NC_000964.3_2100 genome:GCF_000009045 level:species cluster_id:NC_000964.3_2100

MERFIKRLSASCESTIHHKVYQIMNEAKTEFEKVLKKLK*

>NC_000964.3_2137 genome:GCF_000009045 level:species cluster_id:NC_000964.3_2137

MEKMNLLKEITIFDLNKIKPGTKVQVTWYKGTEMEYTHNGEVIINNGEKFYYNYVDKEGYVGHCHVNALD

LKNYPDSLIVEIKSK*

>NC_000964.3_2138 genome:GCF_000009045 level:species cluster_id:NC_000964.3_2138

MKFAPMDKVKFKTASHLNKLRTLKKRVPELDDPLLGECWEFEEDGLRQFDWEENYEFVARPKHFNWD*

>NC_000964.3_2171 genome:GCF_000009045 level:species cluster_id:NC_000964.3_2171

MNAQLFNLESRLDELENEINTQYCELDTNLDALKSNRIELESQLEKFESSLTNRLQGSISNNCRNDLLNL

GYTHSQVDCMSDEEVYAALDKIDEEIHNTDQDYSTGFEDLEKQIIEMKRDYFIDRKERGSGNFDEAWEGE

ILDLEFEYTVLCLEKGLEPLNYIITWEG*

>NC_000964.3_2176 genome:GCF_000009045 level:species cluster_id:NC_000964.3_2176

MKIWIGCGNGSAMGTSVPLILPLDVNIPPYIGIVMRV*

>NC_000964.3_2232 genome:GCF_000009045 level:species cluster_id:NC_000964.3_2232

MYSIIFPVHKVCSLKGVLLIPYERGGKMTVYESLMIMINFGGLILNTVLLIFNIMMIVTSSQKKK*

>NC_000964.3_2243 genome:GCF_000009045 level:species cluster_id:NC_000964.3_2243

MILTQMKLFMQSLNGGYKIDLYNRELQRALNLHFWWICKVIYLVKEIKE*

>NC_000964.3_2559 genome:GCF_000009045 level:species cluster_id:NC_000964.3_2559

MSAFLFIGKIIMTGVHSVESLFSCQKELVFQHITSHKITFSLFDYLFFFFYNMR*

>NC_000964.3_2676 genome:GCF_000009045 level:species cluster_id:NC_000964.3_2676

MINEEKHTNPQANNNIFGRIDTWYSVHRSSNLRENKERPAYYVAGPYL*

>NC_000964.3_2694 genome:GCF_000009045 level:species cluster_id:NC_000964.3_2694

MSTYESLMVMIGFANLIGGIMTWVISLLTLLFMLRKKDTHPIYITVKEKCLHEDPPIKG*

>NC_000964.3_2772 genome:GCF_000009045 level:species cluster_id:NC_000964.3_2772

MGKKLIETIQYPPYYCDVAYLESSHMKSRRNLFQNKKSLDVWQWITLEIYWLI*

>NC_000964.3_2779 genome:GCF_000009045 level:species cluster_id:NC_000964.3_2779

MKGPWRFHKKQYRGFFKAMQDYNGSVQEGAFNPSINDGKGGGFLHSPSTSFDSLHAYKLFRP*

>NC_000964.3_3352 genome:GCF_000009045 level:species cluster_id:NC_000964.3_3352

MTHDLSENETHRKRICKKSQTKKIRLEKTRNHIVKKLAEFRKLFFTPLPGGQRFHTHPALLGALFLLRFL

RYV*

>NC_000964.3_3439 genome:GCF_000009045 level:species cluster_id:NC_000964.3_3439

MKRHLICSGVFFIKSQFLLLGIRSLNMQKTFFSLSSIKKLIVMRMQLNMNGSKKISDMIQIEPFLINKQK

SRGQAALLFI*

>NC_000964.3_4067 genome:GCF_000009045 level:species cluster_id:NC_000964.3_4067

MSLINSHQSADSGLNIWSQLFFYTIVTASEWIGGLQKRRKGKGNGEILYEIIGRKLSLCFIRLSFLADIE

VALVE*

Chlamydia trachomatis

>NC_007429.1_122 genome:GCF_000012125 level:species cluster_id:NC_007429.1_122

MAIIFGELRVSLYPPFLFTYISRQAYPKAILHCADKNFLESLHSNQCFRIANATCSTLSYAEIDR*

>NC_007429.1_162 genome:GCF_000012125 level:species cluster_id:NC_007429.1_162

MTEQPHRLSEEARSIANEAKERLLKLNDKPELSRPYLVKGFLSKISRGISSKEAASKFSTAIVTSLLGYL

WNHILQNKERILSADARDLEARSVLLDTKEILALCKYFAINRCTWLSSSSTQEEENLICQDIAKEMFSIS

LHALVEQQWIREYDLSVNNGSSYKLFF*

>NC_007429.1_168 genome:GCF_000012125 level:species cluster_id:NC_007429.1_168

MTFFSLFNRPLTVDFCSGVIPSFSSMFLGVRSYLLASRFSIFPEEASSFFSSIARFFLDKTAAVGGTALA

EIVFTPSSEQSTYERSLAV*

>NC_007429.1_235 genome:GCF_000012125 level:species cluster_id:NC_007429.1_235

MSYLFCSSCAPTLESPAELCLYKTHIYCKRRGNIEFAVSLGIFAILSCVALLCLLCGGSSLVFAGLGIGA

IMHWFCGFRSRVNVSLLVL*

>NC_007429.1_336 genome:GCF_000012125 level:species cluster_id:NC_007429.1_336

MNTLSFRNAFALISESGLQQQFSGCVCSQMIASYEVVFSQMMHPVTKRWVSLTEGWTEGG*

>NC_007429.1_372 genome:GCF_000012125 level:species cluster_id:NC_007429.1_372

MGLEKGAIYSRSCEERAEIGKQRKIFACIKKSFHLDWRKFFWANLHGDRFQKASRHIYIMVRLLFSFCSY

PYLGKTSEEVEGCL*

>NC_007429.1_503 genome:GCF_000012125 level:species cluster_id:NC_007429.1_503

MKKLLLLAMLTSAAAAGSVYADETEEEKEVSSLVVSLACEGEEGGEETRDPIIL*

>NC_007429.1_848 genome:GCF_000012125 level:species cluster_id:NC_007429.1_848

MRAFCKQIEMMKARLFASCFSPYSIDWIVFRNEKDSPINMKKQGEEDVYAE*

Enterococcus faecalis

>NZ_KB944588.1_128 genome:GCF_000392875 level:species cluster_id:NZ_KB944588.1_128

MKKRMEQIEEILSCEENSAGVRLKELVEALELEVTNQNLLKVTSILHMNPKFKKIYAYEDSRVITLYQLL

QNKPLEVTE*

>NZ_KB944589.1_20 genome:GCF_000392875 level:species cluster_id:NZ_KB944589.1_20

MTKKQASPFLETESCCCMVQNSAAYSFVMGKTSVISEKLPNTIPL*

>NZ_KB944589.1_204 genome:GCF_000392875 level:species cluster_id:NZ_KB944589.1_204

MENREKIIQLFKNPLVTGYGIEIMSNGRLYSANFQRYKNRVKKEENPLIFLRA*

>NZ_KB944589.1_205 genome:GCF_000392875 level:species cluster_id:NZ_KB944589.1_205

MTAKVEQLFLELAEEVIRTNPKTKQEFKEMIKEYSYKEDNKW*

>NZ_KB944589.1_209 genome:GCF_000392875 level:species cluster_id:NZ_KB944589.1_209

MKFKSVLFLFLASIILFGFSQQTNAESQESNVTPSQESYAHDVLNEQLSSNLSTTDAIQQRAAYAPSTWY

YTTYRSYVGYPPATIYVSVENTAVERRSHGYLTLISTGYAPGNWRTYAGTLYLQGGPYPIPSSLKATDLL

VKQDN*

>NZ_KB944589.1_219 genome:GCF_000392875 level:species cluster_id:NZ_KB944589.1_219

MAERLLLFLISNYQLSTTLYPVKTSYDVFHFKHFCGLVYA*

>NZ_KB944589.1_220 genome:GCF_000392875 level:species cluster_id:NZ_KB944589.1_220

MLDDLPGVADNAKKILFDMRCTTVIDDCNVHLRDVLNLLEDEMHSFRLSSIEANDLDKLYDTFYYYGKKE

QLIEFLLDQMTNAINFKQADKDFLIYSWLVEKLTEEQICEMLARINENTQYHWNNNLNSFVDEFVAYYKE

TNGIDLRINYLGAIYTNLKILSDEYTLSISDYELIFKFFEDIIESHSETIFDFQRKYKKDIETNFGNSLE

NFPKLNKLLFE*

>NZ_KB944589.1_258 genome:GCF_000392875 level:species cluster_id:NZ_KB944589.1_258

MTTTTVYYASNNEKQPNRFQLVIGSCSYELPKIEIYEILECIALAYGYQKEPFIEEQTECFVKGNRCIHV

SQQATTLTFVVNDLRLVMEKQCRNHEEVFQEVNHILLNIFEETPCVFLEK*

>NZ_KB944589.1_266 genome:GCF_000392875 level:species cluster_id:NZ_KB944589.1_266

MENFSSKSQPSLNKRAGTKDSLTFVPARLLIHQ*

>NZ_KB944589.1_398 genome:GCF_000392875 level:species cluster_id:NZ_KB944589.1_398

MEKMRNVRQLRRTFLVLNTLFVLSVFRENRWTKSALLFWLLANSFLYGLQWLIYFGGEKGWLTHLQRWYY

CAVLLQIVGTLIVYFSFFV*

>NZ_KB944589.1_541 genome:GCF_000392875 level:species cluster_id:NZ_KB944589.1_541

MAKATKMNVNYRESILDRAYRLELIKKDEYAKHQEKLSLLNEKLSDGEPAQFDAKEETFWKKISQKVIL*

>NZ_KB944589.1_615 genome:GCF_000392875 level:species cluster_id:NZ_KB944589.1_615

MAKTKQEKVARNNKIGQYIAIATGLYYLGKMILDRRQKK*

>NZ_KB944589.1_630 genome:GCF_000392875 level:species cluster_id:NZ_KB944589.1_630

MKLNGIIMIAVVGSVLSSCGWQKSKEESQKVQTVQTM*

>NZ_KB944590.1_1 genome:GCF_000392875 level:species cluster_id:NZ_KB944590.1_1

MTTMLAIIGTVAIITVQVIEYNMTEIHIPTAGTFNDDDAEPPHSSVTLVFIRTILITLSLNFPSHFFEMV

FLCILENNKQKRLLNQ*

>NZ_KB944590.1_29 genome:GCF_000392875 level:species cluster_id:NZ_KB944590.1_29

MVKDAAYIIEEITTILEGTYFVITKKANKRRMCLHVSDTAKEFLRCRLEYETFLEQQFDYATMFLETCTE

DVSVEIQTGLLEETNYQALTVEKLQQIATKM*

>NZ_KB944590.1_40 genome:GCF_000392875 level:species cluster_id:NZ_KB944590.1_40

MRAKLFLILRNRVLSRVVFVILFLHVLFTTNDFTVLLLLLLLNYLLTLEDSAKKNRKIQTWQEFKQSIDW

PFVAFYSGLALLVISLILLFLWFNYAR*

>NZ_KB944590.1_119 genome:GCF_000392875 level:species cluster_id:NZ_KB944590.1_119

MASWFWLSGGLAVICFMFFLFSLLTSMSFIQHAKEPRSILKETLLSWLMLLLSGGWITIGLLLYFSLKNQ

*

>NZ_KB944590.1_158 genome:GCF_000392875 level:species cluster_id:NZ_KB944590.1_158

MSKCFGLLMILFVLSGCSGSRNSQQEVVIEKSANNDKARQFVDQKNPHLGTQLEAESYTKQEAIEFLQNG

LGIPTNDSAFHYTFKQSNKGSYVVQIISEKNSVSKENELFGYYQVYQDGIIVKMMEKDQTFQEN*

>NZ_KB944590.1_171 genome:GCF_000392875 level:species cluster_id:NZ_KB944590.1_171

MLAGLFSVTGMILLGILFCLIGYQTFRKHHH*

>NZ_KB944590.1_191 genome:GCF_000392875 level:species cluster_id:NZ_KB944590.1_191

MALVISMTILVSLWLIQSQKQAVKVPVKIERNRNN*

>NZ_KB944590.1_214 genome:GCF_000392875 level:species cluster_id:NZ_KB944590.1_214

MKRIVAYYKNGDRYLKEMGSGKSSLHFFLFFFVLHSMREHIIQFIGFWPSFIFVLILVPNLMLGVGHINH

WYKNRS*

>NZ_KB944590.1_217 genome:GCF_000392875 level:species cluster_id:NZ_KB944590.1_217

MSQNYKKTLSDMLLLAIILLISSVSIKIGAIVIGMIGLMELLTE*

>NZ_KB944590.1_221 genome:GCF_000392875 level:species cluster_id:NZ_KB944590.1_221

MSNLTKRKKDLFEMKSVVFKDISKQQSEKAQKRKRLLQLMNQYPDWASQKNKLIMQEIQELGQAIGNWSM

DQSRPIQSIKAASFTKSEYLYLIWLGYSDEAIRHGLDMSKECYFIYRLTLLNE*

>NZ_KB944590.1_375 genome:GCF_000392875 level:species cluster_id:NZ_KB944590.1_375

MEMYQVIRYFLNEMEKKIWHALLTLVLFCLYISIFDVPFTIKNTFIGRLLFPKAFMNAGSANLIFYLISF

FGFLWSIFIFAYLLNKTRLKLLNKTFPYLLEILEVCILPLVILFAYIYIYLRVHGEGFSYIRESLLQQEL

TYSMKWFLCLGSLAPLFFFLSTLNLTKRVRQKKDMES*

>NZ_KB944590.1_818 genome:GCF_000392875 level:species cluster_id:NZ_KB944590.1_818

MKKIIKHFMKPVEIFETLDDDIGMFLIISICTALIFTFFSK*

>NZ_KB944590.1_859 genome:GCF_000392875 level:species cluster_id:NZ_KB944590.1_859

MPEFDSLGARQEPPEEKEVLELTWEYDEEEDER*

>NZ_KB944590.1_862 genome:GCF_000392875 level:species cluster_id:NZ_KB944590.1_862

MYVAIGEASRETYVIGETQAEVMRKLFEEYPYVSADKNVYPERLSIVQKEPRTSANEQGKY*

>NZ_KB944590.1_863 genome:GCF_000392875 level:species cluster_id:NZ_KB944590.1_863

MKAIRETRLVGAFLLMIVLGVLLKSHFSMPILATLSAPFFIHWFFNWDEAKYQYSKKGGDKKCM*

>NZ_KB944590.1_866 genome:GCF_000392875 level:species cluster_id:NZ_KB944590.1_866

MDNLVIMKNQQAVTTSLQVEQRLIKIIVMF*

>NZ_KB944590.1_910 genome:GCF_000392875 level:species cluster_id:NZ_KB944590.1_910

MRTHQKKLFLFSMILWLVEFLITRISDSFPGATPLFVVICLIQGILFTYFCLAFFYYPQ*

>NZ_KB944590.1_929 genome:GCF_000392875 level:species cluster_id:NZ_KB944590.1_929

MKKTIGTILTVITVSILLSGCSKAKYPIIHFTQKEFVADGNGVVEIKGQFLNGEPGTLEANINRKAGKVK

VDKDQNFHIRYQMDSIKDTDFYLGIKDEKNRIAVGTTKIDSSEVEAVQNDFEVIRTSDIISYFNKYDIKF

LDYNENNVAELSGLTSSVVFNVGTRMDGTSIKEGVYLFEDPESWNKANLSILDWWNKKVSNSLNKNYNDL

DKDSSVNFLYEPNFIDSAEEKMIKQIDFFDNNRFLWTYRDKEKFLILVSDPELTSIERGKSMLAIQSLLI

DKTFSLDKDEEDSERG*

>NZ_KB944590.1_934 genome:GCF_000392875 level:species cluster_id:NZ_KB944590.1_934

MLEELSVEVSSLDDVEFSVSAVSAEITSSALSLLDASIAYTIDELGTNVVSISIVITDILFILFIIYLTL

LFTYSLQLILI*

>NZ_KB944590.1_986 genome:GCF_000392875 level:species cluster_id:NZ_KB944590.1_986

MNDDPYDYLDADYEEYLRKEEVNESTKETSSD*

>NZ_KB944590.1_998 genome:GCF_000392875 level:species cluster_id:NZ_KB944590.1_998

MGKKKSKIKKKKRRLQEKAIANGTQNSKK*

>NZ_KB944590.1_1000 genome:GCF_000392875 level:species cluster_id:NZ_KB944590.1_1000

MEQLLLTKTGENEIGINATGMDDNEIVFTLAAALIGYSKELGLTEAILNESMSVLWKDGE*

>NZ_KB944590.1_1003 genome:GCF_000392875 level:species cluster_id:NZ_KB944590.1_1003

MKRLKISYIDLAVIIESIYYGEDEDVSDIDDLLKYLRNNGHLSTVLTVSRGISDE*

>NZ_KB944590.1_1006 genome:GCF_000392875 level:species cluster_id:NZ_KB944590.1_1006

MKLTSVTFKPSAERFPPIVAIDLDQLTPDEYVTLRNLGYDTQLSKITKRTFEELEGHLGIRGDVAKKNGF

YVLVK*

>NZ_KB944590.1_1013 genome:GCF_000392875 level:species cluster_id:NZ_KB944590.1_1013

MFKAVGKDSLKIYVVEDTKALVFQKLKEKYPDTAINKAVFPEALFIQETKK*

>NZ_KB944590.1_1014 genome:GCF_000392875 level:species cluster_id:NZ_KB944590.1_1014

MTRKEKLQQTKKLADLWYQQQKNQIYIMQQKERREFRCLKQ*

>NZ_KB944590.1_1552 genome:GCF_000392875 level:species cluster_id:NZ_KB944590.1_859

MPEFDSLGARQEPLEEKEALEPTWEYDEEEENQ*

>NZ_KB944590.1_1554 genome:GCF_000392875 level:species cluster_id:NZ_KB944590.1_1554

MEQQIKTYFDGHQSRSITKINVYKSITQIKTEEKQ*

>NZ_KB944590.1_1555 genome:GCF_000392875 level:species cluster_id:NZ_KB944590.1_1555

MNVFDVIGIVAIPLAILCFHNWVISERLSEAENRINSLTIQQMNSRPTFQDSRTGAVLPTRQQAIPPHMK

TKTKSVLNEHETEMVKEVVLEKIDVLKNNLQFMQSNQRKHNSVYTLNQLERQLTLYERIYKKMSDDEE*

>NZ_KB944590.1_1556 genome:GCF_000392875 level:species cluster_id:NZ_KB944590.1_1556

MADQVNIDLLGQAYCNVMTRKTGITHTYTIKGKDDKKQNEKNVQKPK*

>NZ_KB944590.1_1583 genome:GCF_000392875 level:species cluster_id:NZ_KB944590.1_119

MEITMWIWICAGLAVLCFALFLLFLVNSMNFVHLAKPKQSLVKQSMFSWLLLVMCGGWVAVAILLNIALQ

TQLT*

>NZ_KB944590.1_1584 genome:GCF_000392875 level:species cluster_id:NZ_KB944590.1_1584

MMTMKSKGSLLVTLGILLTVGIASLIVSSESFAEEVGQTNIGVTFYGGKEPLKTEGVIKPIEQPVTDKDK

KTSQQQDKVSRKTTAKTNPTNAQTSLPRTGERNSTWLYSLGIACLLVVLTSFYYLNKKRKKEK*

>NZ_KB944590.1_1685 genome:GCF_000392875 level:species cluster_id:NZ_KB944590.1_1685

MNQKWQKLLPTGTHLRGGSAQAITTDPCALYEGTRGKQILAFPA*

>NZ_KB944590.1_1830 genome:GCF_000392875 level:species cluster_id:NZ_KB944590.1_1830

MKKKRYLMIVCLLSSPSFFINVEASDGGSSSVGIEFYQNPRTPAPKDAPPKTDAPAADPKEPAGPPKGDQ

RSGGSTQTTTTGSQLPRTGSKSQANLSILGLALIGLAGMVHRKKGRHEAN*

Escherichia coli

>NZ_CP033092.2_158 genome:GCF_003697165 level:species cluster_id:NZ_CP033092.2_158

MTRSLELRVCIAHKSIPALVEQGMKYCCRMALHAEAQRHRVWLLIIR*

>NZ_CP033092.2_210 genome:GCF_003697165 level:species cluster_id:NZ_CP033092.2_210

MSIDFTPGVINTYNGDIYNCTTNTDNVKTPDTPKWPCDNREEQHPINAPFSGEWIHL*

>NZ_CP033092.2_787 genome:GCF_003697165 level:species cluster_id:NZ_CP033092.2_787

MQQKKYSFNYLIFKHDFIKRRMLVNPATHPVGVSVSEQTLPQCDQQVDNAVPGFAQKL*

>NZ_CP033092.2_830 genome:GCF_003697165 level:species cluster_id:NZ_CP033092.2_830

MVSMRAGNNPRCFRLLWYPRPTQAIIINGKKCIHCYEINSPDVLFNELVF*

>NZ_CP033092.2_1205 genome:GCF_003697165 level:species cluster_id:NZ_CP033092.2_1205

MALDSYASPNIIPELRQPNAAAGKITLGGLDRMAVTPSVKKRRLSMVEFPYAQTLLSDYTAWKAL*

>NZ_CP033092.2_2166 genome:GCF_003697165 level:species cluster_id:NZ_CP033092.2_2166

MSKNDIIIRTHYESPLRMHIDSDILTPSSEPINQFARQLITLLDTSDLSSMLSYCVTQEFTASCRKISHN

CYTTALFIISFTTSPIHAENTLITLHYKKEIISLFLETTPIKANHLRSILDYIEQEQLTAEKRNHCMKLS

KKNS*

>NZ_CP033092.2_2336 genome:GCF_003697165 level:species cluster_id:NZ_CP033092.2_2336

MNRQRLDSPLVYVGAHNVYYVKLPVKVLYFTIHIHTQINQYVVISDFPPVSVEKFNLCSVMAHHRP*

>NZ_CP033092.2_2346 genome:GCF_003697165 level:species cluster_id:NZ_CP033092.2_2346

MHATTVKNKITQRDNYKEIMSVIVVVLLLTLTLIAIFSAIDQLGISEMGRMARDLTHFIINSLLD*

>NZ_CP033092.2_2597 genome:GCF_003697165 level:species cluster_id:NZ_CP033092.2_2597

MLRLLRHYFYFVPAQLSKAKGIGKNILLKLQLVIGISLTASPHHHVIFATS*

>NZ_CP033092.2_2683 genome:GCF_003697165 level:species cluster_id:NZ_CP033092.2_2683

MLLSGESVKEKKTAALFVVTFIFIGYASRNTRRDKTETHKASQWLARPHMY*

>NZ_CP033092.2_2849 genome:GCF_003697165 level:species cluster_id:NZ_CP033092.2_2849

MSLSLVVDLRLGLLIPPVYAIYEKKARTFVRALL*

>NZ_CP033092.2_3123 genome:GCF_003697165 level:species cluster_id:NZ_CP033092.2_3123

MHCSIINSLFTMPFYKNESGIIFFNFICLLVFLVKLSIIHCFIILRMMEIKGFLMIIKHILMLFNHEFFN

MIMYQYVILPDVLLNYRLLL*

>NZ_CP033092.2_3252 genome:GCF_003697165 level:species cluster_id:NZ_CP033092.2_3252

MFLFFGDGELMVTEAIWLLLQYDQLRYSWLAAKNFDD*

>NZ_CP033092.2_3407 genome:GCF_003697165 level:species cluster_id:NZ_CP033092.2_3407

MSLTTTPGYVRCAMPFGANDHCDFAAMHATIHHLIYLLSSPVPQQYLEIFADIYINLDEL*

>NZ_CP033092.2_3453 genome:GCF_003697165 level:species cluster_id:NZ_CP033092.2_3453

MAVQLFKTLLNQIPLFSSLQSGTLPLFGYSGRVRAMKKAHTGESGLKWEAKDSSKLIGNKAHALRGLSSP

MVGIRQIRLIT*

>NZ_CP033092.2_3473 genome:GCF_003697165 level:species cluster_id:NZ_CP033092.2_3473

MLTVSGNEQVTIARNISGVILKLSGISSVVYLCMHSTFISQIEQVSLEFAGEIVTMKHESYLSVLYKDSV

LIVER*

>NZ_CP033092.2_3836 genome:GCF_003697165 level:species cluster_id:NZ_CP033092.2_3836

MITGCGNDKDKFQCEAYLYPHPLIILDLEEHFTIQG*

>NZ_CP033092.2_3849 genome:GCF_003697165 level:species cluster_id:NZ_CP033092.2_3849

MVLCPLGAVNQIFAGCGVNALSGLQRAILRPLLISNYTISTGSWHVQVGKKTKTGQPNISLIDQ*

>NZ_CP033092.2_3862 genome:GCF_003697165 level:species cluster_id:NZ_CP033092.2_3862

MQLILSIYHVICSVTSKINIKNSAKFNIRSTSRLTYDIISQLHLPQNGYFTERSNFLVAIIMLILRSLAI

IPAWFVSLGHEIVTFLFILEIWGNAGDAFSSTSVGNYGFGHYDLNLPQSQLFAGRYGKQTK*

>NZ_CP033092.2_3898 genome:GCF_003697165 level:species cluster_id:NZ_CP033092.2_3898

MKRGVSRKYNESSQDMHYQVIPEGIKLSPRIRIYNISDNKTS*

>NZ_CP033092.2_3901 genome:GCF_003697165 level:species cluster_id:NZ_CP033092.2_3901

MAEPYRYTGWLLRKDASARTRDKEGVVFVRTGREAGVLIIAELFSEVVTVSQPVPGEEETDITDGIRLSD

WYYKYKKSSESNVKDLPE*

>NZ_CP033092.2_3906 genome:GCF_003697165 level:species cluster_id:NZ_CP033092.2_3906

MVASGAVCSDAFPGNGLPVRGLWSLIPVLVAFPLILCQQPPVTDRLPRHSIRISSNRGLYCILLCRFRCA

FPGASAPA*

>NZ_CP033092.2_4098 genome:GCF_003697165 level:species cluster_id:NZ_CP033092.2_4098

MWGFLTDCLVIPFVSPVTGVCIVAVLVYVSLCICTGSARFEKEYPLQITEALLSEPELFLLL*

>NZ_CP033092.2_4107 genome:GCF_003697165 level:species cluster_id:NZ_CP033092.2_4107

MKRVAIPAPALRKSALSPMLATLPLIFLLTPLVCQLFVIIS*

>NZ_CP033092.2_4113 genome:GCF_003697165 level:species cluster_id:NZ_CP033092.2_787

MQQKKYSFNYLIFNDDFIKRRILVNPATHPVGVSVSEQTIPQCFQQADNAVPGFTQKL*

>NZ_CP033092.2_4251 genome:GCF_003697165 level:species cluster_id:NZ_CP033092.2_4251

MGDFFGLCVLPDATLMGDAHLIELNKTYTKNIDLRHIFALYRKLATVKEVLL*

>NZ_CP033091.2_7 genome:GCF_003697165 level:species cluster_id:NZ_CP033091.2_7

MAKSCDYEAIRVFISVNVGKYTNNAITVNYSDSVCATRHYHSTKINSGG*

>NZ_CP033091.2_8 genome:GCF_003697165 level:species cluster_id:NZ_CP033091.2_8

MNDLRTPLHYTNKHHRLGIYKFSNKSIIFTETK*

>NZ_CP033091.2_70 genome:GCF_003697165 level:species cluster_id:NZ_CP033091.2_70

MHKSVAEHSDLIPDEHEWIFRKQKSLYMRREMAR*

>NZ_CP033091.2_72 genome:GCF_003697165 level:species cluster_id:NZ_CP033091.2_72

MNKLVSDGSVKKINYPVLYESGITPPLCEVSAPEPDAGGKRIVAYVYKSSRSTVFENPDIVKTCTVRDLK

KDFVNCDEKGEGQ*

Helicobacter pylori

>NZ_CP011487.1_433 genome:GCF_001653475 level:species cluster_id:NZ_CP011487.1_433

MSLGKGLISLIKDDTIPIETRLHENKLTIISKTDNIEIQNIEFNRGNCSDTAYNKGSERIEKESEEELAR

EYFYYELESDRNFIAKNEKTI*

>NZ_CP011487.1_434 genome:GCF_001653475 level:species cluster_id:NZ_CP011487.1_434

MQNKVQELLDSNKSELNLIDETDKAQDNSQEFEAKKTAMSKLKFGESLTIEHDCKNLMEIKIQTDKDSKI

LHFKLPQPKE*

>NZ_CP011487.1_555 genome:GCF_001653475 level:species cluster_id:NZ_CP011487.1_555

MKDYEDELEDFGEEELEGFEEEYEEYGDYKNVYDDDDYEDFNSDYEEE*

>NZ_CP011487.1_776 genome:GCF_001653475 level:species cluster_id:NZ_CP011487.1_776

MIDKVFTALNFLLASKETLEQQDKELKEKHLYPSDKNNCNTNNQTTAIPTKICLFTAKILKNQ*

>NZ_CP011487.1_779 genome:GCF_001653475 level:species cluster_id:NZ_CP011487.1_779

MLDVENEQKISVQLTKFSLKNICLGQRQD*

>NZ_CP011487.1_821 genome:GCF_001653475 level:species cluster_id:NZ_CP011487.1_821

MKIYFAQNLGQRFKNAKRMIMISKSQNKLKHGLKKFKNPFNKEGIKRVVTLTLIERMGVRKISLKSV*

>NZ_CP011487.1_900 genome:GCF_001653475 level:species cluster_id:NZ_CP011487.1_900

MPSDFKNIFNGLKDEYQCFIYEPIIRANVKLLNSGYLWS*

>NZ_CP011487.1_1051 genome:GCF_001653475 level:species cluster_id:NZ_CP011487.1_1051

MIIPENYTLADDIEKELLVIKPPFANIQFISEEDFIYEGIEKVLNVIMS*

>NZ_CP011487.1_1052 genome:GCF_001653475 level:species cluster_id:NZ_CP011487.1_1052

MGTLILQTLDNIIDEVDDIITQEQYFFISNKILSIVNNIYDIRNFTFNEIKQIAEADDRSSFSTLN*

>NZ_CP011487.1_1282 genome:GCF_001653475 level:species cluster_id:NZ_CP011487.1_1282

MGIKIPLLVFKRIDALKHEIYRAKSVGLLSLSFFRILSIVLDK*

>NZ_CP011487.1_1453 genome:GCF_001653475 level:species cluster_id:NZ_CP011487.1_1453

MIEITFSGINDSNEFVSHGRFVQFTDKQGLGCPLHKLTKKGN*

>NZ_CP011487.1_1464 genome:GCF_001653475 level:species cluster_id:NZ_CP011487.1_1464

MAHHEQQQQANSQHHHHHHAHHHHYYGGEHHHHNVQQHAEQQAEQQAQQQQQQKAQQQNQQY*

>NZ_CP011487.1_1479 genome:GCF_001653475 level:species cluster_id:NZ_CP011487.1_1479

MKFLKFFAKSVILFYSIMQTYGDIGDILRYPNLLRMLLKSYCIIKIKIKLFYNQHPIRTKPSYFYSISDS

*

Klebsiella pneumoniae

>NZ_KN046818.1_32 genome:GCF_000742135 level:species cluster_id:NZ_KN046818.1_32

MDSVYPHGQLQTRFPQLPAFQQSKSLSFVKLDQGGGIADR*

>NZ_KN046818.1_393 genome:GCF_000742135 level:species cluster_id:NZ_KN046818.1_393

MASLPGAVMKKSSGYGSALSLARAISVSARAGDSGSLGCGSGQPYSQRALGQFGLQPVVGGSHVAAAHIL

AAAQQALRRRRVFQRFRQPEGAVYGVGEALPAAGAALAQ*

>NZ_KN046818.1_394 genome:GCF_000742135 level:species cluster_id:NZ_KN046818.1_394

MASAKRCQRRERRLLNDIRHVASGLVHRIRHRLQAARAAAEDQFLPVNHAAAVGGFKQHAVRGGAGNQPR

AVHLPDGGDVVGDLLLGRAQLPAAG*

>NZ_KN046818.1_724 genome:GCF_000742135 level:species cluster_id:NZ_KN046818.1_724

MQPFKNFLYQTGLNISIIFVKQAIQVIISIDRIIFLSFINFFC*

>NZ_KN046818.1_725 genome:GCF_000742135 level:species cluster_id:NZ_KN046818.1_725

MRLGMSPSLNIRSYMVFITGSRDSVQKQLRVICEYYAKGQVIIVQKVNWFCCIYKMTSMSLL*

>NZ_KN046818.1_734 genome:GCF_000742135 level:species cluster_id:NZ_KN046818.1_734

MFSYASIMLENHFLIFSIVCSFHLVNIYSFIKVNVYSIDITDGIRLRYYMNAEKGRVSIIIHGLCHLLSP

CQAAGQQLELYRSTYVMLILLVIYFQCGNRKEFSRRESYPTMFHWDSPKFGFVGVREIHPGIDR*

>NZ_KN046818.1_735 genome:GCF_000742135 level:species cluster_id:NZ_KN046818.1_735

MKWMTKYIFRGWGAVLTWGIACGASAELPAWLSTSDIPTLAEPMNVSPPNDIGLTRTTLDVFTCSAGNMS

ANIIRTLWRGTVKYLYPKNGPAAILSSLHILTSPMSPMQRPYSENYCNGRRDGVYVGIWISIGDTNPTKV

GTTYWAEQVQAEPVQCWITASNVDLGDFQPGTQSPAIAIPTTLVCDGDADLSVTIQSLEGKEILTFSPGV

TGLMTVGRSSVSNTTKLSVKRYVSVDPLASVRLTVLPGAKPGAYEAYGVIYVTVQ*

>NZ_KN046818.1_738 genome:GCF_000742135 level:species cluster_id:NZ_KN046818.1_738

MAESDQCPLIRISLDMGNFKLVLNVASLQGERHRFAAPMVPFGENLCLLFTIVRFVLSRQFIKLRIDNFQ

ALLCLNMIKNPALREKTLQHIATAFLLVFFGAVFIR*

>NZ_KN046818.1_749 genome:GCF_000742135 level:species cluster_id:NZ_KN046818.1_749

MSYASECLASQNFGSYAEAESYARTLSGVPVNEDLPWEHLVTSTSTELK*

>NZ_KN046818.1_750 genome:GCF_000742135 level:species cluster_id:NZ_KN046818.1_750

MSLQTHGGHASETGFVKMDPVFYACNLGKGTTCSGMLMTTYYISNILLRNLLSTRVDFDVKLRLRKGNNS

QAHVA*

>NZ_KN046818.1_751 genome:GCF_000742135 level:species cluster_id:NZ_KN046818.1_751

MPPYWIAILNLHCGKNHSHRYDTLQQKQPVGFYDAQSLPDYQEEPPNQPKVDLYELDQRDTPDRKRIHNI

CGGPRHLDHEVS*

>NZ_KN046818.1_820 genome:GCF_000742135 level:species cluster_id:NZ_KN046818.1_820

MVYIFCRDQDLFTPVLIKFLRQTVLIYSLALIIALRYNRENKLMDSFICA*

>NZ_KN046818.1_1501 genome:GCF_000742135 level:species cluster_id:NZ_KN046818.1_1501

MPPSGTTTGTEPDRPAALRLPGLETATAFRECFSDFEAVSTGSFSNNGE*

>NZ_KN046818.1_1667 genome:GCF_000742135 level:species cluster_id:NZ_KN046818.1_1667

MRYGNLSAQGWLSFSEIAMWGDKPLRLRLSMKAIADGPYGRESRFTDMQNMNPETLARTSEAPPGDNRKS

TLSATGHAFRLRALLGRGFLP*

>NZ_KN046818.1_2205 genome:GCF_000742135 level:species cluster_id:NZ_KN046818.1_2205

MGRVAFRVDKGKRSLCARADVNVDNYVTSEAEHNHYQLHLFLFFVES*

>NZ_KN046818.1_2382 genome:GCF_000742135 level:species cluster_id:NZ_KN046818.1_2382

MIFVLRFKGASQKDRNTDLIAASYLYRIHFFNAFKQLCSCCIMTCTLTKKECDHD*

>NZ_KN046818.1_2395 genome:GCF_000742135 level:species cluster_id:NZ_KN046818.1_2395

MLYSVKFYRFDIVGIFIFWVRRFSGKRRYILLIWRKHSDMVHYLKKSWVIILTIFTHPLYCLTFIFIPVT

FNQHKVASMIWITNA*

>NZ_KN046818.1_2469 genome:GCF_000742135 level:species cluster_id:NZ_KN046818.1_2469

MFMLMMKNIINISDHAQRAGQVPARSLYSTLLRNAAVRGLVGALKICPGLPSS*

>NZ_KN046818.1_2471 genome:GCF_000742135 level:species cluster_id:NZ_KN046818.1_2471

MNAIYLAVLDPVFAGNFPNVQPYFVSLLAEVEEPEVLGDVLHNEDLIIEAWAASKALVDYIEDQVDYTEC

NSATNNLQMLKLVLGLQFWICDSEEVEELEQISIKMLKICNVLQEENMLPVHVPASSPVNQR*

>NZ_KN046818.1_2472 genome:GCF_000742135 level:species cluster_id:NZ_KN046818.1_2472

MSPSPLPRIKQLVKLMPAIEAGECTLNEIYVYLNEIRLARIEKDANSKMASEKTLRKCWNLLKEAKQKYP

HLEKYPLAQAAYQIHKGETVKAAPKAKCSEAAYNYARLLAGEPVNGKILVDDANYQLMLKDSEILGRLLS

EETVNGQRLIGEIPFNALEWIKANDQKAYEKAIEATTADLKAKAEEKLQTALAEQEKLLREQIYAQLGLT

APESQADNPAELPAPEQTAPTFTPLGVADAVGKDVAEELKAKKKKESDADFLDRMKALICENKAMDNADY

QRLLDLKGKNNAMATELVELFETSPEEPEAANA*

>NZ_KN046818.1_2474 genome:GCF_000742135 level:species cluster_id:NZ_KN046818.1_2474

MKKVLLAFLPLMLTGCVNPVNQVPAYNPTPVVQPEEAPLVTITPDQVKKLTNEYLAYCNVTPYTFLGKEQ

LASDKEACDKETKYRLKHKKLTKAEEQKNESYAKEMDDFDQKIKEQGQKSLKALEACNENPIICRIHMTD

KELDEGGM*

>NZ_KN046818.1_2476 genome:GCF_000742135 level:species cluster_id:NZ_KN046818.1_2476

MASQITLKLSNKEVRRLKEIAHDLGYDDEGEMIVTVIKMLSTASCTVDEETGELSFYQTMFNNA*

>NZ_KN046818.1_2848 genome:GCF_000742135 level:species cluster_id:NZ_KN046818.1_2848

MNINIPALINFDPAGIKKSTCMYISDLPGIVLYDTIPFTEYSAELLSTYSVKESISCFHP*

>NZ_KN046818.1_2895 genome:GCF_000742135 level:species cluster_id:NZ_KN046818.1_2895

MTELESLFYEMMPGHIAHAMKIEGAKILAESTQKEAERQSTLTGLSSELIIQTAIQAISMQMKNWQVVH*

>NZ_KN046818.1_3239 genome:GCF_000742135 level:species cluster_id:NZ_KN046818.1_3239

MRLLDVPTAILVAALGLPPKHTRPDMYYSKGALCLMATAEGLTPMDFK*

>NZ_KN046818.1_3252 genome:GCF_000742135 level:species cluster_id:NZ_KN046818.1_3252

MICRDESLNADAKAKAKAKAKSMKYLISHKKQSHVITRLCYS*

>NZ_KN046818.1_3275 genome:GCF_000742135 level:species cluster_id:NZ_KN046818.1_3275

MSGAGSSNFDGINVGKDPNFISIQAYANTTGLLAGKR*

>NZ_KN046818.1_3722 genome:GCF_000742135 level:species cluster_id:NZ_KN046818.1_3722

MNIAEEASLIRQLEEARAIINQRNGEILHLQREAARYREQRDSANAMVKFLRGLFENSSQATQ*

>NZ_KN046818.1_3736 genome:GCF_000742135 level:species cluster_id:NZ_KN046818.1_3736

MDFDFVNYSRRSLLLFVMVANIIGWVAIVAILYVAYLAIEWVTA*

>NZ_KN046818.1_3748 genome:GCF_000742135 level:species cluster_id:NZ_KN046818.1_3748

MVGQLQQHPNHPRLLRLSNLKQRNNQQQIQNLRQLMVINLSRLAISQLLLLNHRFQKNMN*

>NZ_KN046818.1_3767 genome:GCF_000742135 level:species cluster_id:NZ_KN046818.1_3767

MHDLICTSVTGIASSYFVVGETYSADEEWRLTTPNPDGSLALWTVEGNMIYGIVGDHDSEVLAKFEGL*

>NZ_KN046818.1_3774 genome:GCF_000742135 level:species cluster_id:NZ_KN046818.1_3774

MPCYSLKNFIIDLVNVFSLKIYNMNNACHMAIRAGVNYQGRSDPRLLFSKVVFGYRLVNGRCRIFYITFI

LRLTRLAHNRQRQSGNDQR*

>NZ_KN046818.1_4173 genome:GCF_000742135 level:species cluster_id:NZ_KN046818.1_4173

MRLNHHSFLTINTRHGITTARTAVIVDGASLDDGKDVIPLHLRVCSPFEQHHAQTTAVDRTARLRVKSMN

QAILCLQPITVRFIAALLLHAKRDATR*

>NZ_KN046818.1_4246 genome:GCF_000742135 level:species cluster_id:NZ_KN046818.1_4246

MQINDNFTYHLMGMRLICQYFYKSWKTLTFPFC*

>NZ_KN046819.1_27 genome:GCF_000742135 level:species cluster_id:NZ_KN046819.1_27

MWSNYYISHSSRISQFTTIATSHIDNYVIKLFIELTQRLADVSIFLNKGYAR*

>NZ_KN046819.1_41 genome:GCF_000742135 level:species cluster_id:NZ_KN046819.1_41

MISETDLNLYSFTSTATIFGPNKSVICIFIEIH*

>NZ_KN046819.1_53 genome:GCF_000742135 level:species cluster_id:NZ_KN046819.1_53

MQEGLLATLILFFFHHLSFSCLTYKNTQLRLRHIR*

>NZ_KN046820.1_13 genome:GCF_000742135 level:species cluster_id:NZ_KN046820.1_13

MSYQWHCFYNQYSNIVFFQIQGEWEINQDASLACLWYFV*

>NZ_KN046820.1_46 genome:GCF_000742135 level:species cluster_id:NZ_KN046820.1_46

MSAPYASMLRHAVNIFTSFPVNTGNGIMGMQAFLMLNPDIKCFTKGCTTKNIIEPTGSIFAKHQILTVSQ

RKSLFVAPLDDRLHGCPESNFSIINQLKHRQ*

>NZ_KN046820.1_92 genome:GCF_000742135 level:species cluster_id:NZ_KN046820.1_92

MVAYQDRLSQRDSRCQRINMRKRLLRFNRPPVTVVTEPPGALLLAIKQGAVLVLDITGEGKQDILPPRRP

GWRHKEFLFGRPAKACFVDQGIRFFIESQ*

>NZ_KN046821.1_15 genome:GCF_000742135 level:species cluster_id:NZ_KN046821.1_15

MNDRYYKLEINTEMVVESLFKFYKEYYLSRYEKIEDVDWDEQSIMKHHEYYVENKGEVYYLFMVDMYKEY

QKEKYEISESEFLYPNGAGFKKRTSYNCSGFFESDENILIDVKNVKDDELLTGLKVKYEEFISNLKGVGK

*

>NZ_KN046821.1_19 genome:GCF_000742135 level:species cluster_id:NZ_KN046821.1_19

MKKYINILSANVNYLLATLFVFMVILTFNIYDYGHVNVSVLMKECGLCIVNFALLYVIFELPRKALK*

>NZ_KN046821.1_55 genome:GCF_000742135 level:species cluster_id:NZ_KN046821.1_55

MGYGLALWVGFYTPLISLYGMGYSPSHKGKLGEY

Legionella pneumophila

>NC_002942.5_21 genome:GCF_000008485 level:species cluster_id:NC_002942.5_21

MARDEPQEITAVCWSKNLPGAAFAPLNNTPGCINTYLTKSGLNLIDIGI*

>NC_002942.5_70 genome:GCF_000008485 level:species cluster_id:NC_002942.5_70

MDELKKRAYLALTNISRKVILTLLIELRSNYEQFEK*

>NC_002942.5_457 genome:GCF_000008485 level:species cluster_id:NC_002942.5_457

MFLTKKLGVFLSPEKIKLEGNPLECYKSSSPQIFKLLALAKSIIA*

>NC_002942.5_742 genome:GCF_000008485 level:species cluster_id:NC_002942.5_742

MQGDGTCPDDIPYAESRYASNPGALSAYSVVIAWGEGGATTLVETFAWFIVAAHYKHELAKIVEGHRGYP

DDVVAQAEIMAKKILSSTKLTLYSEIMS*

>NC_002942.5_982 genome:GCF_000008485 level:species cluster_id:NC_002942.5_982

MQSEFGKVEGNQRRLLDIGDSHSGWCLNQTVAFIQTNGKQDKSIMLGLAPVSSQSRE*

>NC_002942.5_1063 genome:GCF_000008485 level:species cluster_id:NC_002942.5_1063

MTKLGLGSASSPRMLFVRAIQPQTWIVPCMPFWLSLISFFSSSLLSMFISSSLTLTIPISLALFRMKLPD

SCLPISKSTTFTCVNFSTLSFELRTPSLPMTHVELGYYERNHRYSNSPVTKPINAFRSLTTRTIT*

>NC_002942.5_1163 genome:GCF_000008485 level:species cluster_id:NC_002942.5_1163

MPSRSLASDLMFFCQGFETMLMYLLSPNVTWGSTGFYILFIYNSRYLVDRFRSCLFCRHELFNKKLFNHA

LL*

>NC_002942.5_1251 genome:GCF_000008485 level:species cluster_id:NC_002942.5_1251

MKFLLAQLFHYLELLNVKSLVQKHLGLDI*

>NC_002942.5_1265 genome:GCF_000008485 level:species cluster_id:NC_002942.5_1265

MSPEKIKTTVTCELKSRAKIIKENYHNPELTLGNVLEALAQYYGFKDWNTAFAKLKDTPYNSSAYPAENS

ERLDRIINKILDGRRNKCDMSYRRNHNYITFLKKYIDKGLETWAPINWDDFVLKINLKFSDDYVFSVNRM

LANVYHKGLFNE*

>NC_002942.5_1323 genome:GCF_000008485 level:species cluster_id:NC_002942.5_1323

MQDDLKYYFWAGPDLIAVAILLLEVTNERQKSGVGLDCSLQVFSIRGHMMEMVAMGGLEPPTSAL*

>NC_002942.5_1603 genome:GCF_000008485 level:species cluster_id:NC_002942.5_1603

MNYQLELVADLVMPLINLSLNLVWSCLKVTKIVYTHSDKKNGFCPE*

>NC_002942.5_1923 genome:GCF_000008485 level:species cluster_id:NC_002942.5_1923

MIYYCIFEINPTIIGLINKLNRNLRLSLL*

>NC_002942.5_1984 genome:GCF_000008485 level:species cluster_id:NC_002942.5_1984

MSLNLKNIELFLETNDLQCYPGFIVTGLVNPNSQIKNCKDRTYAPTSTVPSAIMHVPAEEANNDPRSSYG

QQSK*

>NC_002942.5_2123 genome:GCF_000008485 level:species cluster_id:NC_002942.5_2123

MNSFLQFHKGKQEVLLSPGELAVIMGDENHIQRYLDKVIEPHFRSANRADCAEKRNTRAAIAEGSDCMS*

>NC_002942.5_2149 genome:GCF_000008485 level:species cluster_id:NC_002942.5_2149

MELIAGTEAGGFLLLLLKLKPENEEVYSVL*

>NC_002942.5_2238 genome:GCF_000008485 level:species cluster_id:NC_002942.5_2238

MANTRTSRFFYKLDFFEGLHAPFRNRTGLTKNSKVEAKNVFNEGA*

>NC_002942.5_2306 genome:GCF_000008485 level:species cluster_id:NC_002942.5_2306

MPYYLLTKIFDELFKFFRELSLREVIASFLYHFTKSFGFTQIKNYPGPFSILKRPGSYDR*

>NC_002942.5_2326 genome:GCF_000008485 level:species cluster_id:NC_002942.5_2326

MTHVVQYVAIHVGGKTQQDFRKSLFSLLDMSPFEDRESKLNPLPANFPKWDNLEEEGG*

>NC_002942.5_2400 genome:GCF_000008485 level:species cluster_id:NC_002942.5_2400

MTLPKKLWSSASLDSSGAGYKDIILVEGYFRF*

>NC_002942.5_2410 genome:GCF_000008485 level:species cluster_id:NC_002942.5_2410

MLHDILIVNAHESMLKNSPLSYNLSKLLRIWFPSQVALPLA*

>NC_002942.5_2416 genome:GCF_000008485 level:species cluster_id:NC_002942.5_2416

MKRKRHLLSYQEFSINTLSVTTSILLVAQDFYKVLNKIN*

>NC_002942.5_2548 genome:GCF_000008485 level:species cluster_id:NC_002942.5_2548

MRRVILVQQFSIPYHVVRDNNGKDKQLELYIYKSY*

>NC_002942.5_2553 genome:GCF_000008485 level:species cluster_id:NC_002942.5_2553

MYRLPIFYLSEIGLSCESIQNEMVQNEGLAHLKVSEKFGSVRKATPFVCGE*

>NC_002942.5_2567 genome:GCF_000008485 level:species cluster_id:NC_002942.5_2567

MTQSRFVKDVISLDEFSCKLTIIVNLNRIAI*

>NC_002942.5_2575 genome:GCF_000008485 level:species cluster_id:NC_002942.5_2575

MHCKTHHAYKRLGRPTSELRIIIYLYIYFCLFEKIFFKSDPQKNL*

>NC_002942.5_2735 genome:GCF_000008485 level:species cluster_id:NC_002942.5_2735

MRKAQGDDEKASWDAKYGAFQMLISWEVILISPST*

>NC_002942.5_2758 genome:GCF_000008485 level:species cluster_id:NC_002942.5_2758

MSNLIELALRNAALDRTKLLSQVQKIEFLNGYDPRFVEGLISLEMIGDIDEYVASLMYVKMTDKYTQEEK

EQLTDTILTINNDRLISCQYRFPQLYQKLMQDQFLSTFFSSSHLLPNIAPGTNNQSYETVSTNNKMKMES

AKLYGHYEYYQCVVALITQIDGSRYFYHLNPNRIFGDYMGSAPLKLSGSELKDVKDITFILHPRSMVDYR

EFQRLQERNIVFKTVMLPVGAKSVSLSYDTDKDVLTVNNLEAGQTVTYNNLLQTPQYNRYQYTPEQYFMT

QLYRGQTWAEALGEALDKYIVTRQEGATWWDLKPLFFNRKTTKNNMIAAARIKKEIEAGATSFDFTGVSR

NGLLNQLLTTAVDYQLEYQRGLQNGVLSAEDLTTSAALR*

>NC_002942.5_2926 genome:GCF_000008485 level:species cluster_id:NC_002942.5_2926

MRNKDEKAKEVATDSRCAEANRLTQLITGAILADNCFFPSGVYSIKPMVSSPQPPQEKTTPSM*

>NC_002942.5_2965 genome:GCF_000008485 level:species cluster_id:NC_002942.5_2965

MELATTGNEFISFAVAVVTFLAGWKLSSTNISSYFLS*

>NC_002942.5_2967 genome:GCF_000008485 level:species cluster_id:NC_002942.5_2967

MDKLNWEQPEVCAIPIDSVSASTEEADVQDGSSESGLSSSSSSGG*

>NC_002942.5_2969 genome:GCF_000008485 level:species cluster_id:NC_002942.5_2969

MQIILAVYICDILLINAGILRFYGYFSFFTLYLLWALVIFVKTEKHYLLIEIKKRISICFTKMAKIQSVF

K*

>NC_002942.5_2994 genome:GCF_000008485 level:species cluster_id:NC_002942.5_2994

MKICLFICHDDELEIIILIKHSGLSYIAQSNKGLLTEAL*

Mycobacterium tuberculosis

>NC_000962.3_7 genome:GCF_000195955 level:species cluster_id:NC_000962.3_7

MRMAWSTVGAHIGQRPGQAAYQMLETRRRGSVLRLGNPKRGIVSRRRYHTLRGARPTRPPPPMLG*

>NC_000962.3_14 genome:GCF_000195955 level:species cluster_id:NC_000962.3_14

MRLPLPVTPVAAKGERTWREGVRLNGPNGVSVYRHVPWRVHKVYSSDEPT*

>NC_000962.3_62 genome:GCF_000195955 level:species cluster_id:NC_000962.3_62

MPVVTAVGRRRGFAMPWVSTARSGAVMLANYSAGVCGRVSSPGLNVRKMCLKANTPGAVTWLDTPKRFLS

TQTASRCMAVNSSDVVTGRIDPQVLHTPLNTDVDGYAHAMHSSINSGPLEYLPATFSVFPALGDVGDLGG

GVGAATYALDRLSNMRSGACVGGGESPWRSLMT*

>NC_000962.3_69 genome:GCF_000195955 level:species cluster_id:NC_000962.3_69

MELSVSVIAGLVIALLAAITPAAGERPESRRQALANAAEAGEHPATSPLRR*

>NC_000962.3_209 genome:GCF_000195955 level:species cluster_id:NC_000962.3_209

MSFLVIEVTGIFGAGGAHARMDAAHEFMRLFGFCPKGKT*

>NC_000962.3_297 genome:GCF_000195955 level:species cluster_id:NC_000962.3_297

MPTRGTSPAKIGGLQNNSGPGKGFGISQNISHIYAVGASADGSWQHPWAGAERSLVSGWDLHCGAPWCGI

VVMKIMFISPLWCLRL*

>NC_000962.3_353 genome:GCF_000195955 level:species cluster_id:NC_000962.3_353

MVTTCWSCWSATDFGGLLSVIMEPPGSPSWMSSPPDQPLPNSPPLAPLAISGPQQRLGRRIGGGLGAGTR

ARTHVQVLCKLAMERRRLIVEGVMPPTVCGNQCRDGRRHLVFGRRQHAGLVALRREVADPRCWRLVSLWW

PGPGGGAQWARWGRGRPGKNAAPWPGHERSRQARRRPACAVQEPHGPHRVPCSPSAATEPVSP*

>NC_000962.3_397 genome:GCF_000195955 level:species cluster_id:NC_000962.3_397

MVSGRWEAGNADGNGGSAGLIGSGGAGGDGGSGGATGAGGEGGDAGASGSINGNAGDPGNSGERGAVGKP

GAPG*

>NC_000962.3_475 genome:GCF_000195955 level:species cluster_id:NC_000962.3_475

MDTQPATCRCGTGPKSGLKLTGVKARSFATNPEMLSTIRGTARWAHQPPFGGLILATSYEIVISITHGIR

RPIAYH*

>NC_000962.3_599 genome:GCF_000195955 level:species cluster_id:NC_000962.3_599

MKRAGPAAIGPSIRVRTAEVSHHRSAAEFASADASPGFAE*

>NC_000962.3_700 genome:GCF_000195955 level:species cluster_id:NC_000962.3_700

MHGGLIDSTASIWSYCPRPDSQPWQAATRAFSPEATKGAMRALWVSQREDLTADAKRVNLLGSMRRMWPK

EVEIAS*

>NC_000962.3_724 genome:GCF_000195955 level:species cluster_id:NC_000962.3_724

MAAGALVVGAFGGHIVWLSGRLGQRAGRTHPCDRRVDIATVHIGTALGCGLRPAAPGHQMHARRLPAIRD

AARFDGPPQHVA*

>NC_000962.3_734 genome:GCF_000195955 level:species cluster_id:NC_000962.3_734

MRAAGPGSAARTCRTRWLPRRAYCWPTGGGELPTPAIWCGWMARSVGLRGRRRRARRSALRSGIHLRSRE

YSGRLGCRRCLSGRGGRTRSPSKRLRQTGLRRRQAGLVIPASAATVWSLIVRWVIVVSICSRRRTRRSGA

RWGQYRSAREARRWRRVGRVIVASAVGRRFCRPTVPTWLGPIRGAVWQSAGPLSASCSSSSISSRLNPWD

YARLTDETTDTASARYRDSPENDRAGAASRSSARNSAASGR*

>NC_000962.3_876 genome:GCF_000195955 level:species cluster_id:NC_000962.3_876

MQKQRRSTVWDNQRKTFGVVISPARLTAWIAASALILLTGTELFGTALSWVKLSPIWHVFSFAVDTRPIA

TLLDYASHPIDPRSYSSVAVTAVGAEATLLAFFFATVGVVASTALNTLRSSPPLLLSSPRLTTGCDR*

>NC_000962.3_928 genome:GCF_000195955 level:species cluster_id:NC_000962.3_928

MRLAGNVGNIPIPIDCTLRGLTQYSRTNNAEVVQSVETHHRPANFDALT*

>NC_000962.3_961 genome:GCF_000195955 level:species cluster_id:NC_000962.3_961

MMCYRPAGSPLPGPEPATSGKRAPLDESPRHEKLDGGAGIVAHDVMLQGAGQPMAFGVPLTGSISAAGDH

ATVASIAQVARRATA*

>NC_000962.3_1099 genome:GCF_000195955 level:species cluster_id:NC_000962.3_1099

MSVPRRTPNSHAPAGPVPDFLRHRYETSTHRVICLRSVPKTSGIGAVEALTPSTAGRAGLAQRALPRRAG

RPLGPHRPRHLPTRRRVDRRLGSDRGRHAPPRRYDLPGLRTHAPRPDRRDPRRAGHRHPPLVEDTGQHRR

DCVAPLRPGHILDRTRRDHDPGIGSDNRNLTHPSARSPIHSGSATKSVTNWRVTRCESGCAEAANPPG*

>NC_000962.3_1117 genome:GCF_000195955 level:species cluster_id:NC_000962.3_1117

MQSVRQIFPQDIFIGRTMGVVVERYGSTDLSSRGMFVPFHDVDCVQ*

>NC_000962.3_1165 genome:GCF_000195955 level:species cluster_id:NC_000962.3_1165

MADEPRLEAGAHPFEEGRDKAPELRATQMDHVRFTEGRRERNRDRLERSQQFRQPGR*

>NC_000962.3_1191 genome:GCF_000195955 level:species cluster_id:NC_000962.3_1191

MCRLVRRVPAGGPGFTGPGVGPARGGVISRHPALPVLTQVYEPAFLVALLTTIRTASNLARRRYGPWNGP

RRSRAIQHSAATIRAVERPGNGGPTRQNLNPSGASNVADNMESIAGGAIPPFGLGCIAGPARIVCIGPGF

SSESVLLRW*

>NC_000962.3_1264 genome:GCF_000195955 level:species cluster_id:NC_000962.3_1264

MCRTTPNTLRRFRSEGDRRASGFRAQSRYPHWTLDGNQALWMRAFNTAGLSRSFNTAGVSRPFNTAGVSR

PFNAVRPSSRR*

>NC_000962.3_1412 genome:GCF_000195955 level:species cluster_id:NC_000962.3_1412

MHRPSNVATATRKKSSIGWVLLACSVAGCKGIDTTEFILGRAGAFELAVRAAQHRHRYLTMVNVGRAPPR

RCRTVCMAATDTPRNIRLNG*

>NC_000962.3_1419 genome:GCF_000195955 level:species cluster_id:NC_000962.3_1419

MTDFYALSLTIFASQDSRIYCDGSGQDDFGGVLRVATAVGEVPTGL*

>NC_000962.3_1497 genome:GCF_000195955 level:species cluster_id:NC_000962.3_1497

MVLQRGRHWRPGHCAGIAADAGVARRARRRRVRGCGASHPVRPGRGPASARTCGRRGRRTGQNRLTRHRS

ASWRRIGQRILDPGHANDGSTDHTIPGSTSQDRPKRNQRSSDRYHRSRNGGYRYRCARYRYRSAGDWYRC

ARYRYRCARDWYRCADGHRCSCRHWPRRDERNTSHVSKCGTARSRGCRSTGSHRVAGGYRRVARHALDAR

VATRHQRVSRRVRA*

>NC_000962.3_1503 genome:GCF_000195955 level:species cluster_id:NC_000962.3_1503

MFGHERNRDSATASSLAMHLAGAFELVSGPKWTACRFEAGTVRIPSQGKRRWQA*

>NC_000962.3_1509 genome:GCF_000195955 level:species cluster_id:NC_000962.3_1509

MFLGQATIWAGWAILCGRLPVATGLAVFVGIWFVASP*

>NC_000962.3_1590 genome:GCF_000195955 level:species cluster_id:NC_000962.3_1590

MPSSGTALAHPDQSLEDIRTGGLTDLKDGPQGYLMALSVVESAATGPIPHAPSIEARRAIYQDLGM*

>NC_000962.3_1615 genome:GCF_000195955 level:species cluster_id:NC_000962.3_1615

MRLAPATKRAVRYGPLRSGAIQPSCLVTHAAFVSAGKDGRYAHLLATRNSDLSRCRGQIVHLAALRLVRQ

LQPIQRG*

>NC_000962.3_1644 genome:GCF_000195955 level:species cluster_id:NC_000962.3_1644

MGRKLSRRNKWRRWPPDRGAGAAESQAAAARGAGSWPPGSIFWFGAHGLQTRIRASFDENRHCYRRSPRH

LRRHLPAAQTNRQRNPQRRQRNPPVRGETNGAAGRRIAELERPKAKERQREGGRLGASITNAGFGPMEPK

PSQRHTDKEVGAALGISAGTYKRLKRIDNATRSDDKEIRLFAEKQMAPLAAGSPSWNGRKPSSGNRKAAT

MAARLDILAWGPWAPSQNRSVVRRKQTLLSAQPSASPPAPTGGSNESTTQPAASWRVGGPAPLSRGRPRL

ALSYLRGSLHLQNSKRVAHQHI*

>NC_000962.3_1649 genome:GCF_000195955 level:species cluster_id:NC_000962.3_1649

MAETPDHAELRRRIADMAFNADVGMATCKRCGDAVPYIILPNLQTGEPVMGVADNKWKRANCPVDVGKPC

PFLIAEGVADSTDDTIEVDQ*

>NC_000962.3_1657 genome:GCF_000195955 level:species cluster_id:NC_000962.3_1657

MLAQFPGGDQRAQPGQQPVVVALGLAAGVAVDGGRIGGGVDDRGVVVGVGRAGGGQFG*

>NC_000962.3_1780 genome:GCF_000195955 level:species cluster_id:NC_000962.3_1780

MSPWCIDYVPSYFVGVASAFALPACAFTTIDPVADMYRDGVSPEPGRQTLPTVTIIPLWGINTRVTRPKP

FSQKISYGD*

>NC_000962.3_1795 genome:GCF_000195955 level:species cluster_id:NC_000962.3_1795

MTEALCDKLVGAWDLVSYVERAAALALGYLAYGGR*

>NC_000962.3_1810 genome:GCF_000195955 level:species cluster_id:NC_000962.3_1810

MYTRFDLPSQDGATAFGQILILLLRRTASAR*

>NC_000962.3_1812 genome:GCF_000195955 level:species cluster_id:NC_000962.3_1812

MLGPYLVVVTATALAHASSRTSRLLRAIGGQAVMVLMALSGGISATSAGFDETLELGGRTTAAGGR*

>NC_000962.3_1849 genome:GCF_000195955 level:species cluster_id:NC_000962.3_1849

MLKWTRPTRLTRQMRGGPVAQFKAREEELP*

>NC_000962.3_1966 genome:GCF_000195955 level:species cluster_id:NC_000962.3_1966

MSIRAQSRLISVSVTAADRASVLRGLTNGMAVWIYRGFLRIAARGYRLCSPREAELAFTA*

>NC_000962.3_1981 genome:GCF_000195955 level:species cluster_id:NC_000962.3_1981

MPAASVGRAEPEAPVAGVVPAVAAGRAVEAEMAA*

>NC_000962.3_1995 genome:GCF_000195955 level:species cluster_id:NC_000962.3_1995

MAGGRRHHDEICCGVAAAGVSDGDGAVPEIDGCIVDPVLRWFQGRDRAVNLSCVRAVAQGIFAEPVGSRP

*

>NC_000962.3_1999 genome:GCF_000195955 level:species cluster_id:NC_000962.3_1999

MLSDGVCGGLSKIQRTGVHLRRPAVATTIAAPVYGPAPGEKKPRSIWPMLLTPELNAAVR*

>NC_000962.3_2029 genome:GCF_000195955 level:species cluster_id:NC_000962.3_2029

MKDADDLADYGLSIEQVRAAVDSHVDVDHSVSAL*

>NC_000962.3_2042 genome:GCF_000195955 level:species cluster_id:NC_000962.3_2042

MIPTPSIGAVINAKISHRACRTFPRPTDIHPRRYLPRKHGGTNPRRLSMNPGGMRIRCRRGDKSRKLLSR

SQVQPLVGRPAKIPSPAANAPPSRARTASPVFENLELRAAAGLAFGFRLRPFGGTAADSPPVAAQDLDPC

RWADSPALHLAVGVETMVVGQLDSPSFGQGVPLVAGHWAPGETGIGRDNISRVNGGSARRPVRS*

>NC_000962.3_2050 genome:GCF_000195955 level:species cluster_id:NC_000962.3_2050

MLLPRPQEVSDERLASLRGIGHPYHPCSILNIGLTKYIIAPVYAITLQFILITLLRVHISNRHIALLAPQ

EAVDEFAVRNDMSRR*

>NC_000962.3_2069 genome:GCF_000195955 level:species cluster_id:NC_000962.3_2069

MPKDRLPDLTPTGAYAPANSGMTMARQDGPR*

>NC_000962.3_2153 genome:GCF_000195955 level:species cluster_id:NC_000962.3_2153

MFGNCDCVGAAAELGGVRSIVPGIGRVEVRQPVGDRCRDRLGDIGVVEDVFVGRVVVAHGAEQRVGIDRL

DTRMAGQHLVDPRVVPAAVVNHQLRVDDRGGVRGAGLVRMRVGMGAGEDGFDGYMPAGDRACHAAPHIPF

PFARPARPPDHRNDNHFQYNLVAAEDRAPWKTTGVVVGMDGGFHGCPVSSSLFSAVTPWAGIGSPHGSWC

H*

>NC_000962.3_2170 genome:GCF_000195955 level:species cluster_id:NC_000962.3_2170

MGVAWVLLAISAIANAVKGSLWWDIWSLGLLVLIPAVVYGKMRRSRRISSDQDR*

>NC_000962.3_2177 genome:GCF_000195955 level:species cluster_id:NC_000962.3_2177

MVMLVLELSAGRYLLKMNLAPWEQCRMVMLVLELSAGRWLSSNDWAQTEQHRMGILQASSPS*

>NC_000962.3_2221 genome:GCF_000195955 level:species cluster_id:NC_000962.3_2221

MIGDGANGGPGQPGGPGGLLYGNGGHGGAGAAGQDRGAGNSAGLIGNGGAGGAGGNGGIGGAGAPGGLGG

DGGKGGFADEFTGGFAQGGRGGFGGNGNTGASGGMGGAGGAGGAGGAGGLLIGDGGAGGAGGIGGAGGVG

GGGGAGGTGGGGVASAFGGGNAFGGRGGDGGDGGDGGTGGAGGARGAGGAGGAGGWLSGHSGAHGAMGSG

GEGGAGGGGGARGEAGAGGGTSTGTNPGKAGAPGTQGDSGDPGPPG*

>NC_000962.3_2229 genome:GCF_000195955 level:species cluster_id:NC_000962.3_2229

MSPDPQEGGQALPRQADPTGAVELFRKPANRGAVPRGTLVDGIEQEVCVNEH*

>NC_000962.3_2307 genome:GCF_000195955 level:species cluster_id:NC_000962.3_2307

MLANGRRKSSHPVYTLVLSGRTEPIKPAKG*

>NC_000962.3_2385 genome:GCF_000195955 level:species cluster_id:NC_000962.3_2385

MLEKCPHASVDCGASKIGITDNDPATATNRRLASTIRKPPIEHAAGPLGSTSRAGHRSYGGVAS*

>NC_000962.3_2410 genome:GCF_000195955 level:species cluster_id:NC_000962.3_2410

MTARPRHYAKPSAARSHNQRAMIFRPWKKSLLARQRWDTGHVGAKRLHSPHE*

>NC_000962.3_2413 genome:GCF_000195955 level:species cluster_id:NC_000962.3_2413

MEFAPGAVDTIASDNMAAQNVHDTAVKTSR*

>NC_000962.3_2480 genome:GCF_000195955 level:species cluster_id:NC_000962.3_2480

MVMGPLGRPRPGNGGAGGSGAPGQAGEWDSDDRLPACHVAVFTVAR*

>NC_000962.3_2670 genome:GCF_000195955 level:species cluster_id:NC_000962.3_2670

MAAAGSVDCIDHASDQISELSNYVHIGRNLGSIPILEVIYVNLWESHCPNAFQAPDANSGERVTNPAGQD

GQPASAGADYASWGPGGQPTGGAGRTASTTWPPDSFR*

>NC_000962.3_2745 genome:GCF_000195955 level:species cluster_id:NC_000962.3_2745

MPLGANITLAELPEPELRQLFPHEELQIPVSCGGLGAGAGGRMDMRAVGLVPVRRASRYCTGFFVLIHSY

LTLMGRGARLRLSR*

>NC_000962.3_2765 genome:GCF_000195955 level:species cluster_id:NC_000962.3_2765

MSGHALAARTLLAAADELVGGPPVEASAAALAGDAAGAWRTAAVELARALVRAVAESHGVAAVLFAATAA

AAAAVDRGDPP*

>NC_000962.3_2771 genome:GCF_000195955 level:species cluster_id:NC_000962.3_2771

MSGGWLAEHLGLSTNRLRHELADRLDAHYGPPAQNRELARPSLRIINEGTDG*

>NC_000962.3_2877 genome:GCF_000195955 level:species cluster_id:NC_000962.3_2877

MTTLPALGYLSGALLHALVDYPTFSDRVLRCGAC*

>NC_000962.3_2923 genome:GCF_000195955 level:species cluster_id:NC_000962.3_2923

MDPGELQMVREVVRPKTPRGDGNWIALTGLALILVVVRPKTPRGDGNLERVTADGTIETSRQTQNPERGR

KPSAQHR*

>NC_000962.3_3002 genome:GCF_000195955 level:species cluster_id:NC_000962.3_3002

MWTSAGPADRHSWRPKPGPTSPPSADRRRPGTRGARPPARRSRWAPTPADRPGPGRLPARSWCVAHPGGP

VKPPAVPRAARRIRPSRCRPRKPADRGARPGRHRGAPDSNWAMRRRTAGLPPAAAAAASPAGSRRHGPAP

HQAATSKGSVRCGSEPSVPLQCSAARAIWPIAAARFVDNVVAVDSAAGKITSWVGVNYSAQLASAG*

>NC_000962.3_3112 genome:GCF_000195955 level:species cluster_id:NC_000962.3_3112

MDVIWSATIATTVATGMRKPRMHGMPPITSGSMVTRVTRMSIRLAGDSTLGRFSTSRLGLSSAKSKPEGD

FGTACGAVSGGDAGVVALAEGVDDGQSKPGAAGGARGVGGFRESRADCGEQFGVASWTPQGEFEFGGQEA

KGVRSSWPASLTN*

>NC_000962.3_3113 genome:GCF_000195955 level:species cluster_id:NC_000962.3_3113

MARIADELTFSLERVLQPLQKVVECCSKIPDFVSSQWQWQTGMWVGGADARGADAHAFHGSKSRGGQQIR

AQGVDEDVWPCPDRCDRASSWFHNHPIDHG*

>NC_000962.3_3244 genome:GCF_000195955 level:species cluster_id:NC_000962.3_3244

MHTSLRVTGVLPDRLLNQRFDVAGVEVDQPGGQPVTVVFAEGGCELAGEVVDVLASVVEVHDRGGFGQDR

GGQVPDPGGAIPRPQVRQW*

>NC_000962.3_3314 genome:GCF_000195955 level:species cluster_id:NC_000962.3_3314

MDHPTEREWASIAEHTRASNFTGDLLRMPPYPLILTLRTLVGSAEVVTASHTLFLSAATEY*

>NC_000962.3_3317 genome:GCF_000195955 level:species cluster_id:NC_000962.3_3317

MWGEAGFEGTTTRIREPTSTREQTQKSPISGEIGDFCVCSPRAPRLTTRRR*

>NC_000962.3_3350 genome:GCF_000195955 level:species cluster_id:NC_000962.3_3350

MGLGGVTMRAGHGRVTTLMLCLFEPRPPCAAPD*

>NC_000962.3_3359 genome:GCF_000195955 level:species cluster_id:NC_000962.3_3359

MLAMIALIKVIRSGGATVTAQCRLPAPQYPVPAQGRHIDHGPQPLALTERGDAADHVAGGLFGGSGFSHG

RFGHP*

>NC_000962.3_3449 genome:GCF_000195955 level:species cluster_id:NC_000962.3_3449

MNGDSASTIDIDKAVTRTPVRRIVRSALDRLWLRQSQPRRYRFLEDSCMARALNRL*

>NC_000962.3_3473 genome:GCF_000195955 level:species cluster_id:NC_000962.3_3473

MGALRSQIQRTTPEPLAEAHPQAFDPAPVVGMGACRRNQRMVIVASGARPARAHAVRCRWLFSSGNSFPQ

AAIQRYGLTEIRLVTIARTAGGSHVPSVNLLVH*

>NC_000962.3_3484 genome:GCF_000195955 level:species cluster_id:NC_000962.3_3484

MLGGKGGDGGNGDHGGPATNPGSGSRGGAGGSGGNGGAGGNATGSGGKGGAGGNGGDGSFGATSGPASIG

VTGAPGGNGGKGGAGGSNPNGSGGDGGKGGNGGAGGNGGSIGANSGIVGGSGGAGGAGGAGGNGSLSSGE

GGKGGDGGHGGDGVGGNSSVTQGGSGGGGGAGGAGGSGFFGGKGGFGGDGGQGGPNGGGTVGTVAGGGGN

GGVGGRGGDGVFAGAGGQGGLGGQGGNGGGSTGGNGGLGGAGGGGGNAPDGGFGGNGGKGGQGGIGGGTQ

SATGLGGDGGDGGDGGNGGNSGAKAGGAGGKGQAGQPNSGTEPGFGGDGGLGGAGATP*

>NC_000962.3_3511 genome:GCF_000195955 level:species cluster_id:NC_000962.3_3511

MNLCADSETGDATRRKRVVDFTLAASGGQPAQPVGVVHTRVGVIFSRVL*

>NC_000962.3_3515 genome:GCF_000195955 level:species cluster_id:NC_000962.3_3515

MWFALVNPEMLAAAATDLGGIRSGISAAYARPLR*

>NC_000962.3_3523 genome:GCF_000195955 level:species cluster_id:NC_000962.3_3523

MQCDGQLYHAKSRAEMATGSRHCFYEPPTHLHGQLWQLAFGQCLHIARSADRGN*

>NC_000962.3_3528 genome:GCF_000195955 level:species cluster_id:NC_000962.3_3528

MYIRTQGEVLTYLFPLCAIAAEAAATSLFKGSFGDFRVCSPGHDGAITAMPSVLAASRIRSS*

>NC_000962.3_3548 genome:GCF_000195955 level:species cluster_id:NC_000962.3_3548

MNETFTGAAQVDADASPQADGAPSAARIVTGLSRLPGSFSGRYAARIVTGLRLDCPAPPRAATRPASSPG

*

>NC_000962.3_3572 genome:GCF_000195955 level:species cluster_id:NC_000962.3_3572

MSLCRFGFQLDQSNLKLDQSNLTCKRISSIFTMV*

>NC_000962.3_3574 genome:GCF_000195955 level:species cluster_id:NC_000962.3_3574

MTINNQVSDADTHGATTGAPVDRHVIPQGLASRNSPGVCLSGRS*

>NC_000962.3_3581 genome:GCF_000195955 level:species cluster_id:NC_000962.3_3581

MGPRRRPSLSTLSETLRVEMFTEPEGEYEENEVD*

>NC_000962.3_3661 genome:GCF_000195955 level:species cluster_id:NC_000962.3_3661

MAAVASGAVSAATGAASPGRPTGTTAAALTSLPAVAAGTGVGPAAAGAARTADFAVRAVAALAAADATDT

VAAGATTAASAAGAAVAAGRAGAGPTVAASRGGPGSAAVAAVAAGTVGADGTAVAARGTTGTGVSALSAN

PTVSAAISATTGVSASAADGAVAAVAAGTSGPAADAVDAATASTAGSTVAAGRAGSAMPAVASAGCAGDA

DGTSRAISAVAAIASDTVGARSGTGAARARVSALSAAAARAAAVAGGAAGDACSTVAAFAAIAARPASPG

GAAVGGAAVTAGSAGATTTTGAAGRAGAYGAGCAGSANAAVAALTPIAAGPRRARSGAGTGLAGVAAVAA

FTAGTTAAACECTVAAGAAFAAGAAVAAVAAVALLAAGAGAGGSAGSAGSAGPAGPAGPACGAGGGGGGV

VCAVSAVAADPAVTAVAGVAVGALG*

>NC_000962.3_3662 genome:GCF_000195955 level:species cluster_id:NC_000962.3_3662

MAPRLGDTAGSPFVVKSGWTTRRRTPGAPSRPLGPSRLDAAAAALAARSAVAAGAAGATGWCLTTVSGAT

AAAGVSLAAIPAGAAVPTTTAGAAIAAGAARAGATAVAAGATLAAGGAGAASTTGSRGHSGLGVAAIAAG

AADTAGPTGAAVAIASRAGVAAVAAIAGRTAAAAGAAVAAGSADPGVAEAAGAAGAAGAAGAPVPAAGAA

GAAIAAIAGRTAGAAGAADLAALAAGTAVAAVAAIAASAAAKAADARAAGAAGAAVAAGAPVAAGVVSEW

AVAADGPVAAIAAGAALPAGAVGASGQATAAGPGPAAEATVAAVATVTARLRSHVCGVICSVAAVTAVTA

GATAAAGAASTGEAAVAAGAARARRAAVPAGTAVAAAGAAVAAHAGVAAVAAAAAVAAVAAARPGTAVAA

HAGDAAVAAVAAVAGAVAVAAVAGVAGVAASAAVAAGAAAAAPSAAGATVSTTTTVAASAAGAIAGITVA

AVTAIATCAGVAAVAAAAAVGSTLPAGPTTASVTALSTGAGGDRATADGAVAAGAAGTAVAAGATLTPGP

ATGAALAAGATLSGGAAVTGGPRGARGAAGDIAAVAAGPAGTAGTTLTTGTAAGAALATLSAVAGRPAGA

AGASGAAGAAAAAVASGAAGAAAAAVGS*

>NC_000962.3_3663 genome:GCF_000195955 level:species cluster_id:NC_000962.3_3663

MSRMHVIGWPRLGLLLGPAVVRLTGGRTLHLVDKPQTARHFVARDLGAHERVQLGQRRPTTRPGLHHGGH

LLAVTRIGHPDHQSVEHVGMPLQSGLDLFGVDLLAAAVDRHRATTQHGDRAVLLDLGVVPRNGVAHTVNR

PERLGRLLLVLVVADGNVALLGERARGSHCLAPGAPVAGRLLPRALQVRCGITRKPAHIR*

>NC_000962.3_3665 genome:GCF_000195955 level:species cluster_id:NC_000962.3_3665

MWRSGVRKANRCDPDQPGDYLVRLMPSDRPSAAVDAYTVGSTDTLGWLTEWPPQLPDRTVSGNMSPASSP

SPGDRPAPQADVFSCPPWAARPTPVSGCPRRTPAGLADGYPRECRTMAVDQVRSGFGEPSAAISRRARTP

VPYFHPHRCRLGPREARQPTHVDLPCGVSGAGRSRTHHPPLVTCPACTSSAGRASVCSLAQR*

>NC_000962.3_3822 genome:GCF_000195955 level:species cluster_id:NC_000962.3_3822

MCIRSLGMECRRRWITCIDVKPGESPIHRRLGLALGHGMPGRCLSPISIAPSSHPGMASPIRSLERLDTQ

ITCSVGTANRLAYL*

>NC_000962.3_3856 genome:GCF_000195955 level:species cluster_id:NC_000962.3_3856

MVVSRADTQPARSQLLHKGRIVALLFAKASCDT*

>NC_000962.3_3875 genome:GCF_000195955 level:species cluster_id:NC_000962.3_3875

MTSRSVAARPTPGFQQRRVQCRHISDVDDQPRRAAPGNDMHPQCGPDTLPGFTELIAHRRAEDHARISDV

HRCAGVLKTARIDFDLRFGLSVSIRPTGNRQISAQRRQVGTKQRREARHMHDRIHSELDGLTENILGARH

VRTQHPRSLTRVGCHQRGAVHDGVASVQRLPYRIAVGYVCNRKVRHVDAQFRDRRLQPCGISHQEPDGVA

RVSDGFGSPPAHKSRTAGDQNAHGGILHWITAQLSLGWQRSSGYRRLAAKFAPWPTMPFTAYRPCATGLL

PSPQIESPSAHN*

>NC_000962.3_4005 genome:GCF_000195955 level:species cluster_id:NC_000962.3_4005

MVEMLGVCVVATDLDGHRIVLRDSEKTLKMRNR*

>NC_000962.3_4006 genome:GCF_000195955 level:species cluster_id:NC_000962.3_4006

MWRRSMGWVGKKKSTAGQLAGTANELTKEVLERAVHRESPVIRPDVVVGIPAVDRRPKQ*

>NC_000962.3_4037 genome:GCF_000195955 level:species cluster_id:NC_000962.3_4037

MGIGGVGGLGGAGSGPAMGMGGVGGLGGAGSGPAMGMGGVGGLDAAGSGEGGSPAAIGIGVGGGGGGGGG

GGGGADTNRSDRSSDVGGGVWPLGFGRFADAGAGGNEALGSKNGCAAISSGASIPSCGRKSLS*

>NC_000962.3_4065 genome:GCF_000195955 level:species cluster_id:NC_000962.3_4065

MRYVTGDHAVQVFQLTSTVIDLTTKRKHTTVVYAATSMSGTPPLHR*

Pseudomonas aeruginosa

>NZ_LN831024.1_140 genome:GCF_001457615 level:species cluster_id:NZ_LN831024.1_140

MCPSGCRQAIRGKRSGTLNPPCEMPGRQLALLWVCLWKSCSHSPLKKCLRRSRERHWTGPQDFSRLAKEP

EKKRCGMRHDSASRNCGQRIHPWRRRACFRRSPRTFLESRKTQYSWASWRLKGKSWRHFHGHSRSAGFLW

KSCWQTVPEAGRRGCPRDSADPWLGRERSSCRAPPNGCRYLVDKAVGKVLEKMPCQVAPMACRVGCFLSI

AMLRKSPGRSAREASQGPGWAPDTLWITL*

>NZ_LN831024.1_251 genome:GCF_001457615 level:species cluster_id:NZ_LN831024.1_251

MPRHPHTTLLLCLCLTLPGCESGRSYSLSEGAPVTGIRIQRDDRSPLQRLRDCERQGCSERQKIDPFTPD

PDYLRAEGHFPLPW*

>NZ_LN831024.1_307 genome:GCF_001457615 level:species cluster_id:NZ_LN831024.1_307

MGRPQFYPKDRQEIALVPAIRALERYREA*

>NZ_LN831024.1_575 genome:GCF_001457615 level:species cluster_id:NZ_LN831024.1_575

MNRPPLRDQSQGVGGSSASFGEGELALAERNPMDVAALFAA*

>NZ_LN831024.1_1054 genome:GCF_001457615 level:species cluster_id:NZ_LN831024.1_1054

MAANENARHDGGRSLCEGCAQCAPSLFSSGWPA*

>NZ_LN831024.1_1667 genome:GCF_001457615 level:species cluster_id:NZ_LN831024.1_1667

MHATFQKRTVFRQDGRQALGEGLIGQAHGVIGPIHMPGSERRTIRQPNCRHGIEKAEELDRDRQTGKAIF

HPTIGIADPG*

>NZ_LN831024.1_1807 genome:GCF_001457615 level:species cluster_id:NZ_LN831024.1_1807

MIFTLVRLDESSVVSKSGAIFAFESRLVLQVLNIRGV*

>NZ_LN831024.1_2031 genome:GCF_001457615 level:species cluster_id:NZ_LN831024.1_2031

MAEKTQDMHLPSLKVHGEVFEDYRNEAIEFNYICVSLLSAKRQRKDNGIYFPIMLGGQPRPAPGSAGVAR

APGRRKNQLTMIPTRNYSAK*

>NZ_LN831024.1_2178 genome:GCF_001457615 level:species cluster_id:NZ_LN831024.1_2178

MRWYSRISLGLSLAVASAFLVAYWRSLVSHNYPNMNAPGLIAYLLVFPACALLLHSPALAFVLQQRKLGS

ARERALQWTINLALWMAPLCFIFASL*

>NZ_LN831024.1_2745 genome:GCF_001457615 level:species cluster_id:NZ_LN831024.1_2745

MPTPAMLPAPTRPARLSIRAWKELSWPAWPRSDSLNTPNMWPKWRNWTKRERIVK*

>NZ_LN831024.1_3170 genome:GCF_001457615 level:species cluster_id:NZ_LN831024.1_3170

MNCAAAPALQREALFRRRDPVSNTHPAKMAVPTTQ*

>NZ_LN831024.1_3646 genome:GCF_001457615 level:species cluster_id:NZ_LN831024.1_3646

MTRQSVLATGPEASDWWCFGSGDEPARESFSAGGRSLAASRKPVGAQSGMAVAEQANRAQASGQDAAPGL

RTFDLLPGNPPKNP*

>NZ_LN831024.1_3650 genome:GCF_001457615 level:species cluster_id:NZ_LN831024.1_3650

MASPHALVRTQGMTFAGLQSGADLAVIEQQGFPDQPASHPTSDQGDPEIPILELCLPGFVETAYRQPVIA

MKDRRDTERIEVTQQSGMKAGGTMQPLLLSHHLDPAVRQADARVSLQQDQALFQIGGRQPVIRIQATDIT

PLARATPAFRAADGPRLGWRRSVMPGSWRASRRSRVEGSSEPSSTRMISKSWKLCRARLSSARLKYLPWL

KQGMTTLSRGFCGSHGVACPETCRSAEAEPISRTRWTRSLPFSPSHERKAGGRENSRRCRKWADLTPFTW

TPVRFPWRPLAQAARPQAGSRVCSWPHP*

>NZ_LN831024.1_3659 genome:GCF_001457615 level:species cluster_id:NZ_LN831024.1_3659

MPELSFRRRSPGLRPRLPWLFPTSRAMVRETAMGKYCNLPIGPDRRQWTTGGARRQSSAYGKGAYSFLSK

PWVFPSCENEKRIPCSVMLSRRSPPGRVAPAAKRRRGPSALLATRAKMPPSSRTMPRSSQSTLISATPST

PTETRFDSASRSSSTVLPHHDIPANLPATVVSTATDGTSARTHFRASGVGKAERSALGAKTSPAKMEATR

SRRFQRAKA*

>NZ_LN831024.1_3695 genome:GCF_001457615 level:species cluster_id:NZ_LN831024.1_3695

MSRVLFLLLAMVLAGLGQPTLAAPLPDELPAALPAAPPVAPLPMGSAPQPQPEAAEPDLPGSDAASADGE

ATTKDGLKIGNGSLRLRQDDPGPTRESPLRDPRLNPYAPRQP*

>NZ_LN831024.1_3885 genome:GCF_001457615 level:species cluster_id:NZ_LN831024.1_3885

MKPLLTALLLVLLGAPAAFAADLGYPLDAPAPTAAAPAARWPKAAPRRRWRPRKTRT*

>NZ_LN831024.1_4135 genome:GCF_001457615 level:species cluster_id:NZ_LN831024.1_4135

MQLVTGVTEALQLAPQVTDVHVQAAIMLGMTAVQDMLVKEGLGQGLAFRGEEGFQQAVFGRRQAGEPLAK

ADIADIGRELQRGPRQRFGAAARRAPRSRAIRRRMASMRTRSSARLKGFGR*

>NZ_LN831024.1_4367 genome:GCF_001457615 level:species cluster_id:NZ_LN831024.1_4367

MNWLKGTAASVGLMVSCAAVADSAAQVELMDNLHQRMLDALQASSVDQVTEVFGDLDRYRPGIRSSANEI

CFKAYEALGWVLSDLVVQADMDDPLPELKGHQMDYERKRLACAAGA*

>NZ_LN831024.1_4368 genome:GCF_001457615 level:species cluster_id:NZ_LN831024.1_4368

MDKPIDPVVLTALLICLSQMSEQDQLDLLRLACAYRDR*

>NZ_LN831024.1_4379 genome:GCF_001457615 level:species cluster_id:NZ_LN831024.1_4379

MHELLKMLDNPRSLLNFSLAILAVLALFFMLKSGAQAASQPASSFSAPKEADSRGKRP*

>NZ_LN831024.1_4386 genome:GCF_001457615 level:species cluster_id:NZ_LN831024.1_4994

MNWTSYFAALGLAFLAYLAGFFFAVAVTPTGPVWPL*

>NZ_LN831024.1_4390 genome:GCF_001457615 level:species cluster_id:NZ_LN831024.1_4990

MEKMKALFRNASIATAGLAVANVSFAESLLDETTKGVLAQASTDGGSVAKLVIAAVAVLVGLALVIGAMR

KA*

>NZ_LN831024.1_4394 genome:GCF_001457615 level:species cluster_id:NZ_LN831024.1_4394

MLADTLKALLLLCLIQAARTVVDPVKGRAPGSSEPPHRSGERKHGRSAPLNASPLKQPPLGSVGAASPPR

APEPSAARAG*

>NZ_LN831024.1_4626 genome:GCF_001457615 level:species cluster_id:NZ_LN831024.1_4626

MLCYAAVICTPRYDCINVDIFDVDAIFFCP*

>NZ_LN831024.1_4770 genome:GCF_001457615 level:species cluster_id:NZ_LN831024.1_4770

MTLSSVLAAFTVSATLVLSKAFCLGEAATIAPCGVWVGLALLAAMVLPLKT*

>NZ_LN831024.1_4986 genome:GCF_001457615 level:species cluster_id:NZ_LN831024.1_4986

MDTFQFCFAGIVGSVSGRVVTWGGLTVDIDQIENAWLRQAIEDYRCGRRGQK*

>NZ_LN831024.1_4990 genome:GCF_001457615 level:species cluster_id:NZ_LN831024.1_4990

MEKMKTLFRNASIATVGLAVANVSFADSLIDETTKEVLTQAGTDGSSVAKLVIAAVAVLVGLALVIGAMR

KA*

>NZ_LN831024.1_4994 genome:GCF_001457615 level:species cluster_id:NZ_LN831024.1_4994

MNWTSYFAALGLAFLAYLAGFFFAVAVTPTGPVWPL*

>NZ_LN831024.1_5039 genome:GCF_001457615 level:species cluster_id:NZ_LN831024.1_5039

MANAFRAKRQPFQDAENLGIVGFQRSGSRFCAGMLSENFPKQA*

Salmonella enterica

>NC_003197.2_192 genome:GCF_000006945 level:species cluster_id:NC_003197.2_192

MLNPTYKLHNINMLWQYFVGLKIAERYQVRELSCHVLSRELCFYFQQEAGVIFVSISHELFQWRAAVS*

>NC_003197.2_287 genome:GCF_000006945 level:species cluster_id:NC_003197.2_287

MLVRWDLFAHDQMNYIYSLYLLREFFHLWFRFWFYISLYNISRNL*

>NC_003197.2_291 genome:GCF_000006945 level:species cluster_id:NC_003197.2_291

MCKSRRGYLFPAFYYQLLINSCSVLLRLITGISPVLFSAPEMFLTTVLHSVY*

>NC_003197.2_299 genome:GCF_000006945 level:species cluster_id:NC_003197.2_299

MYRSPNKVSRGNKQNIARLFLLESPTRLDIICESGGNRTDGLNKTLTQNTNLCAGSSHFVTSLISSFTGA

RGRYLRKAIIPAQQQPLPLGLCTFKISHNQKPAGRSPAPVTCCLQILRNRVYHGLRCYLISAQNQHHHLG

YSLQPLPADDGGGFGMQHFHPGLWLSQLSDVPPYAGPAAKKIRAWCDKTDDVSASPHSFAPLR*

>NC_003197.2_487 genome:GCF_000006945 level:species cluster_id:NC_003197.2_487

MVVFYKKIRSEGKKSTERKSDKYGMDKARWHQDVQAVKAYL*

>NC_003197.2_504 genome:GCF_000006945 level:species cluster_id:NC_003197.2_504

MIHDVNNFAPVFKTARTFLVAQIDNSLIFSSTNH*

>NC_003197.2_550 genome:GCF_000006945 level:species cluster_id:NC_003197.2_550

MGFGHMRILACIGQLPESGLMHYGSVGFFFGTDGALRLLAKKPDGAFVTYDM*

>NC_003197.2_711 genome:GCF_000006945 level:species cluster_id:NC_003197.2_711

MPWRDHVNPDGEVVSCCDNVIITGYHRLFVHPLWMFIMVFDVHSR*

>NC_003197.2_715 genome:GCF_000006945 level:species cluster_id:NC_003197.2_715

MHQSTVTSLLFGSPLCERGDDLGTEQWALAGNDLINFELAANQERSNPSNVTCLSLITVLKEWVNIIVLL

HNLLTGERVTP*

>NC_003197.2_724 genome:GCF_000006945 level:species cluster_id:NC_003197.2_724

MSVFDMVGHLYGGLFYGFIVCGKVRVKHPGYKIIGFVNTEL*

>NC_003197.2_789 genome:GCF_000006945 level:species cluster_id:NC_003197.2_789

MLFVVYVLQPEVPLVIYHSSHDHGDKNGKRRKTFLRYLCCFALNIVLKKILAQGQDFKRSL*

>NC_003197.2_907 genome:GCF_000006945 level:species cluster_id:NC_003197.2_907

MMNNKHTNHHLLKIKDIKTMSYNMMMMHEKSKMRQIPRRCRPVEDLSSRSTFLQMRIVTINLIYVPVVQG

IIFS*

>NC_003197.2_931 genome:GCF_000006945 level:species cluster_id:NC_003197.2_931

MGIQTSKFMPEKSKAKTKKIVVSTQSVLFKKNKHIPVIYYTATIQRRETYLPQYHLIATISFQSLNYCGF

NFTPHPGNCGWDFLTIQVDLMMTQADWIILISTVVIIIFGIILYTVICHIFKR*

>NC_003197.2_971 genome:GCF_000006945 level:species cluster_id:NC_003197.2_971

MIHLQYVLRLRRYELMNEPEFLAVRVMQHRFAVYFILNRGKIAPLQHILEQPHIL*

>NC_003197.2_1000 genome:GCF_000006945 level:species cluster_id:NC_003197.2_1000

MILLNLFVRDTVVPEKAADELFHKNSHLEVWMSEIVVKNCAIRAEKSNPQ*

>NC_003197.2_1020 genome:GCF_000006945 level:species cluster_id:NC_003197.2_1020

MYQRMIFEEGKEPRLYSSQHDSKFIPISNNNGKVQKVPVMELKDTRGDVFYAYNYAYSGKKPSNDEIYKA

IDELKPIGEKFEMSKTISD*

>NC_003197.2_1027 genome:GCF_000006945 level:species cluster_id:NC_003197.2_1027

MKMHNDPHSMDSQSIFAGSQLLPMEKTSHLALSVGFRSHLA*

>NC_003197.2_1189 genome:GCF_000006945 level:species cluster_id:NC_003197.2_1189

MGNSLTRTYTAVDTLWDKTHRTSVRNTNAFFLVGEWIDQETGPHEESEDRQALNCSQQALSSWLELEAVE

TAPQ*

>NC_003197.2_1241 genome:GCF_000006945 level:species cluster_id:NC_003197.2_1241

MKNFFKIITDFIADISLDLFAIFLCMLFVYKTGPSIGVISFFIALIIYIILHFFYSFLEKIIKKIFK*

>NC_003197.2_1329 genome:GCF_000006945 level:species cluster_id:NC_003197.2_1329

MMNLRMPLYINTVTIMCNYQKHLHCLMFVLQMLDSFMLSEAQ*

>NC_003197.2_1533 genome:GCF_000006945 level:species cluster_id:NC_003197.2_1533

MKTRYPCDHLKKSHHVINKKHGFLSTLRDAITTLYLLNITNNIKLIYVFE*

>NC_003197.2_1598 genome:GCF_000006945 level:species cluster_id:NC_003197.2_1598

MLPLRLTRNVYLIYFASYMNYHHRHAEEISIFSTIKMLSVSIQISVLKMAKIFRYVL*

>NC_003197.2_1626 genome:GCF_000006945 level:species cluster_id:NC_003197.2_1626

MFQAKPKLNWSAQKPITYITNIGRKLMLWAEYTTAIIQTGC*

>NC_003197.2_1693 genome:GCF_000006945 level:species cluster_id:NC_003197.2_1693

MRLPNSNVSTSSNATEGNNVPWVAQMPAVFNTGK*

>NC_003197.2_1846 genome:GCF_000006945 level:species cluster_id:NC_003197.2_1846

MVYVSDMFLRRKSIEGEQAIVSSSLRQGITLPWRSQLYFIAMITKHLLANGEIRYLQQRTLTKQKNLHTL

SFYICPG*

>NC_003197.2_1858 genome:GCF_000006945 level:species cluster_id:NC_003197.2_1858

MFVIHLEGYLADSIKRQLWPLLQYPVCHYSATTLVAVGQN*

>NC_003197.2_1859 genome:GCF_000006945 level:species cluster_id:NC_003197.2_1859

MQYSQRGAFAETRREQHGNDDRRQNEFGIGIGKQGAQSLIPTSSSAKSATMS*

>NC_003197.2_1990 genome:GCF_000006945 level:species cluster_id:NC_003197.2_1990

MLTQHRLPEDPRWNALHEEFSFDDDDNLLGMNMPPEGGEHQAKVKGDRLLLQGRPPL*

>NC_003197.2_2219 genome:GCF_000006945 level:species cluster_id:NC_003197.2_2219

MHQVNEVRCDKDIPGRTTSDSTDDVQVKYPDDG*

>NC_003197.2_2372 genome:GCF_000006945 level:species cluster_id:NC_003197.2_2372

MKFSSCQIVGQEYIYYARELDAFLPYIEVKK*

>NC_003197.2_2504 genome:GCF_000006945 level:species cluster_id:NC_003197.2_2504

MKTKTAVMILLIPDVKYLPIKNRILLRENNARLQDSGKERNKNNVCRSFYISLNGRPDKRERHPAWRILS

GFNR*

>NC_003197.2_2507 genome:GCF_000006945 level:species cluster_id:NC_003197.2_2507

MYMGCRPDKTHGVAIRQNAGWRFAYPAYKNTVQAVSKFAMVRTPRPVAPAAHCSTGALR*

>NC_003197.2_2596 genome:GCF_000006945 level:species cluster_id:NC_003197.2_1020

MYQRMIFEEGKEPRLYSSQHDSKFIPISNNNGKVQKVPVMELKDTRGDVFYAYNYAYSGKKPSNDEIYKA

IDELKPIGEKFEMSKTISD*

>NC_003197.2_2723 genome:GCF_000006945 level:species cluster_id:NC_003197.2_2723

MLRPQKLAKGPLKAFKGKGDKCAIVAVNSFCLALPDFLLAWENWHIFSFLDPEELRPASE*

>NC_003197.2_2753 genome:GCF_000006945 level:species cluster_id:NC_003197.2_2753

MGKVMLAGLPGLTHERVGRTSTTYFSSQLTGVNLRFTAVFTEQT*

>NC_003197.2_3010 genome:GCF_000006945 level:species cluster_id:NC_003197.2_3010

MPVPDGGAVAPYPAYENSTCEPDGVALSGYPAFNVISLFQRVGQRDDMQGVGIVANAEDAFGF*

>NC_003197.2_3127 genome:GCF_000006945 level:species cluster_id:NC_003197.2_3127

MGMIIIPCFIVIAIIAVVIFIMAIKACNMEKREDRQMTLAGIILMGVIAAIPGWFLYEIFSHAP*

>NC_003197.2_3344 genome:GCF_000006945 level:species cluster_id:NC_003197.2_3344

MLQNNPDSITKYEGDGCDPLKRQYLLSAGSSCLSRRIVTKKLFQAFI*

>NC_003197.2_3802 genome:GCF_000006945 level:species cluster_id:NC_003197.2_3802

MIKLGQAYNVIRLGKLIPSCSGHNQQNTGVYKSAAEIICLFVFVIYKYYQIHI*

>NC_003197.2_3814 genome:GCF_000006945 level:species cluster_id:NC_003197.2_3814

MTVICLDYDGLMTMEIKKAGPGRHQTSLQGEMN*

>NC_003197.2_3883 genome:GCF_000006945 level:species cluster_id:NC_003197.2_3883

MADAFILLGIVMAMVSLGFILINKLFCFISAGCLLSLCASMASFQLWDASYWGRWGKVCPGLDVIISCDN

YHFLYDLGWELYGIAFLFFTALMLTCAAIILINMIMALERYCAGWRR*

>NC_003197.2_3885 genome:GCF_000006945 level:species cluster_id:NC_003197.2_3885

MFFVITHHIDVLPLATLPTHETCPYCNNQDVWLIIRQKRTRTCGLSQARKRDKFGLAICNHCSNEIKEKR

WSPALRQLFTEQKPLFNLTFWQRYGFWVGWLSVWPALFLATWLYFTFRGW*

>NC_003197.2_3887 genome:GCF_000006945 level:species cluster_id:NC_003197.2_3887

MPKMTPLNRFAHLIKHLKNALRGVGIGSCIYVIIESIMHGTINGVEAAGVLTISAVLGLLSALLDIKAKP

RAPLILLHFLLCGGCVALMVAMLSALNHFPVNASVILITGALFIVIYLPLSFILYRSQR*

>NC_003197.2_4334 genome:GCF_000006945 level:species cluster_id:NC_003197.2_4334

MTGRCPQPGGIANVERQKMTFINHETFICYCELALFVPHTATGGTLLVYYTDYFKDMRLLFFNTERQMIF

QYCFNVSRRAECAFDGKMPHIKQPATRLADRIVI*

>NC_003197.2_4337 genome:GCF_000006945 level:species cluster_id:NC_003197.2_4337

MKFFILFLHSLEEKFHAKDHEMEMFQERTKSTAPDKLSSGTVAKSIKSSNFIKIEESTA*

>NC_003277.2_2 genome:GCF_000006945 level:species cluster_id:NC_003277.2_2

MIMMPVKEATEKNVTMIPGGAGQETLENAAVLILAGMERNDRATTREGNNNLS*

>NC_003277.2_11 genome:GCF_000006945 level:species cluster_id:NC_003277.2_11

MLRWSGNCWPVTLMQTDDKGTIQAGDILPLRALILLLLTSVFISGFLTGVLVVIWLISRGT*

>NC_003277.2_15 genome:GCF_000006945 level:species cluster_id:NC_003277.2_15

MLLAPGITQMLWPQYTTHGYFLSPVNYGYIACLAYVA*

>NC_003277.2_16 genome:GCF_000006945 level:species cluster_id:NC_003277.2_16

MLKLLIASVIIMGGTVITYPAHAKTTTVSTYSQGIYQYQMQASNFMIKSDQAALSMVTMTSFSQCNVQII

SRTCNGTAMVIAPDGTRYPAEVECKVADKGFGVMFRAPGLVQNGIVAGEGYILPAGYTLMFSLSRTGEVR

TTRTLYDECEASSLGSWLHQYHLPSPSVTLTSSSGARYTLGNGVMSTSFPAGGTTKSITVEHPDSINSKA

GVNGAFEQVILKASGTPGASIRVTQSITPSEVARNAQLLKTTGGQCTTMRAGDECKLVLAPGTIRPGHTS

SGTVLISVQLQ*

>NC_003277.2_33 genome:GCF_000006945 level:species cluster_id:NC_003277.2_33

MAVIVTFPAGQRDRRHVKLVAGLQVVKAAGESAEPVQFFQLWDGQAVRLRHRLPLFQGIRHGPDPGEGGI

PVGRERALPDPGRDVRPGRHGRRER*

>NC_003277.2_56 genome:GCF_000006945 level:species cluster_id:NC_003277.2_56

MSWQKLVLLNYTLMCLADAPLGLVQPFACSDASSEQTHGLRCGLTK*

>NC_003277.2_76 genome:GCF_000006945 level:species cluster_id:NC_003277.2_76

MKSQLVAAADRAAMSVAYGQEAADHYGIQYGFIRSVRDWITGFTEGIKGERC*

>NC_003277.2_93 genome:GCF_000006945 level:species cluster_id:NC_003277.2_93

MKVYLQRYGVAGFLLVYWSLSFVNEYFLHKHPLSAESWK*

>NC_003277.2_94 genome:GCF_000006945 level:species cluster_id:NC_003277.2_94

MRLQALLVTGSVVVTVLRLFLLNSVVRREATAIMTGVAAGLHVFALLAPSVWLFYQWQWNGLFLLQWQGA

VFLFCLFALPVSTFCFS*
