# Supplementary material for: Candidate orphan genes: Reassessing uniqueness
Source: PLoS One. 2025 Dec 31;20(12):e0338891. doi: 10.1371/journal.pone.0338891 (PMC12755737; doi:10.1371/journal.pone.0338891)
Supplement: S3 Table — This table lists all 75 candidate orphan genes identified after the four-step BLAST analysis pipeline. For each gene, the table provides the predicted protein structure using AlphaFold3, the species from which the gene was isolated, and the corresponding amino acid sequence for the candidate gene. (DOCX) [file pone.0338891.s005.docx]

**Table S5.** Results from AlphaFold.

| **No.** | **Predicted Structure: AlphaFold3** | **Lineage** | **Amino Acid Sequence** |
| --- | --- | --- | --- |
| 1 | 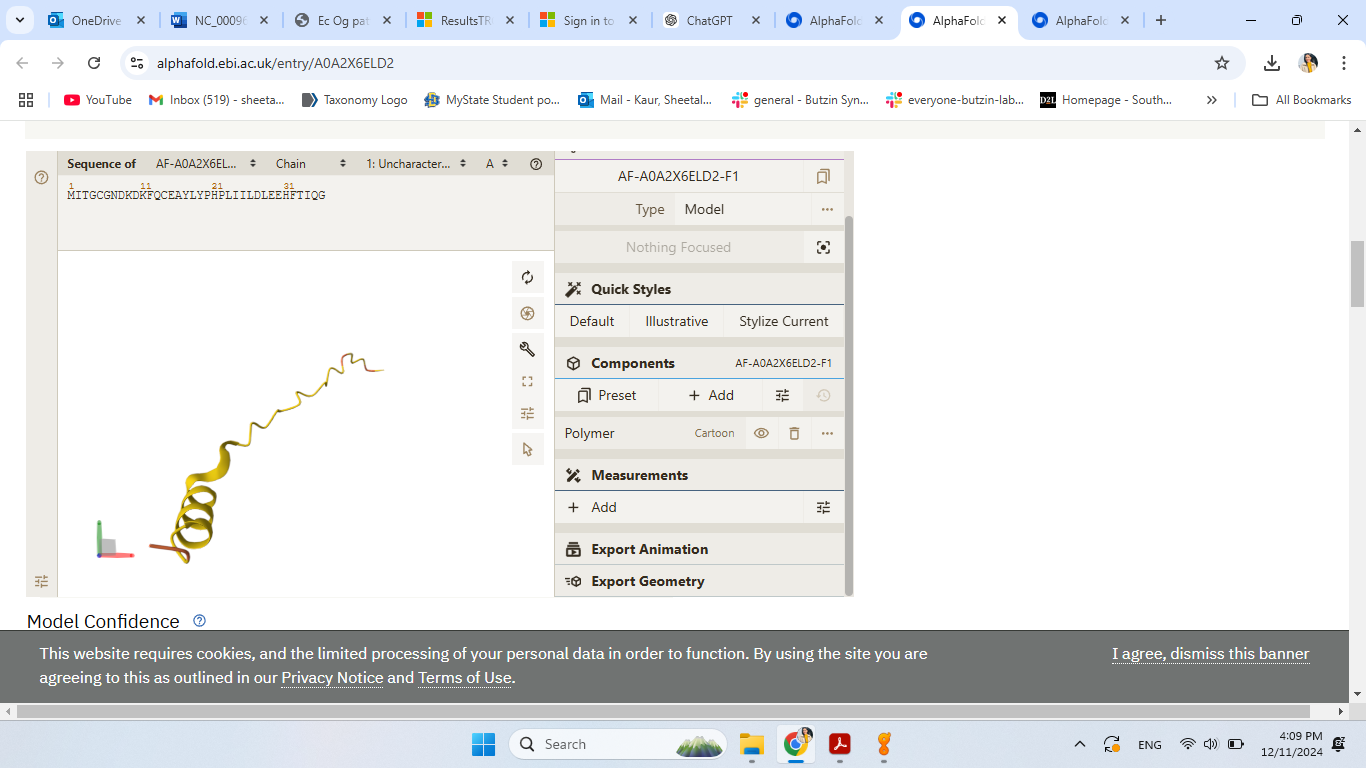 | *E. coli* | MITGCGNDKDKFQCEAYLYPHPLIILDLEEHFTIQG |
| 2 | 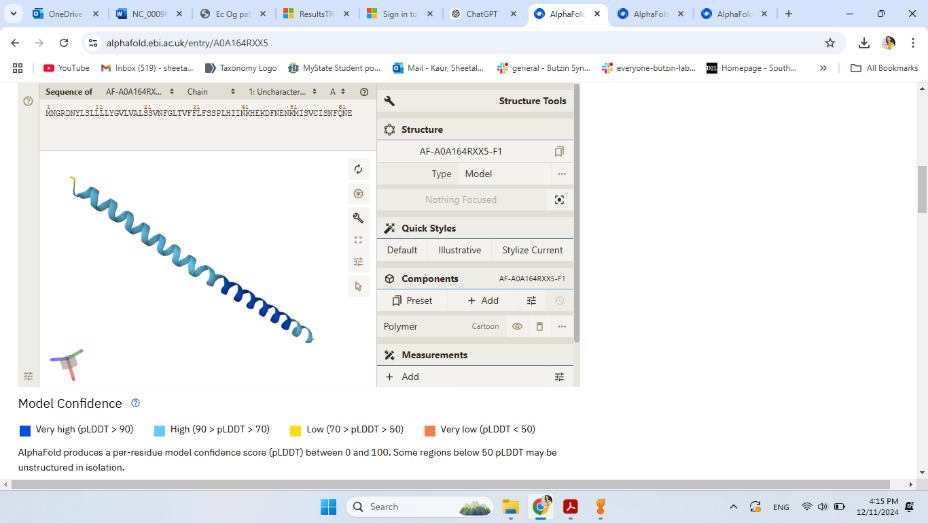 | *B. subtilis* | MNGRDNYLSLLLLYGVLVALSSVNFGLTVFFLFSSPLHIINKHEKDFNENKMISVCISNFQNE |
| 3 | 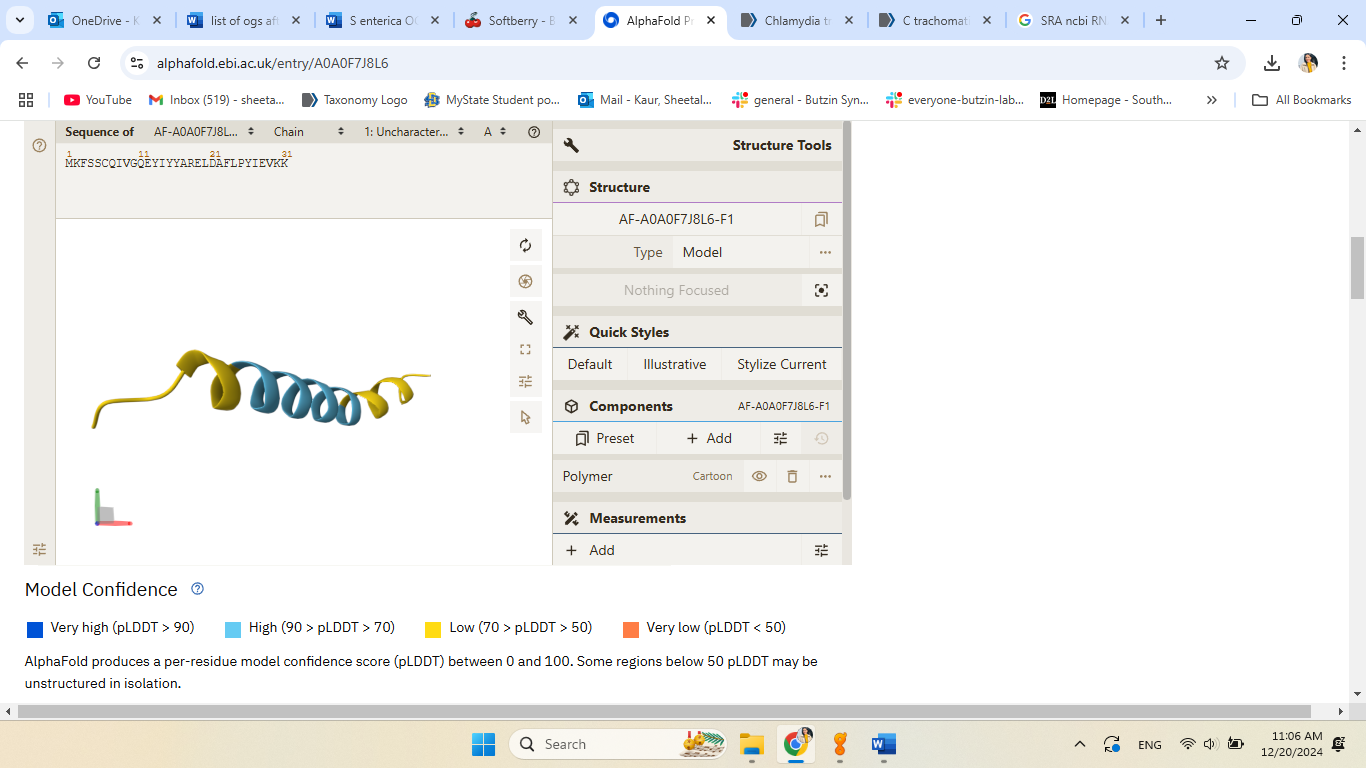 | *S. enterica* | MKFSSCQIVGQEYIYYARELDAFLPYIEVKK |
| 4 | 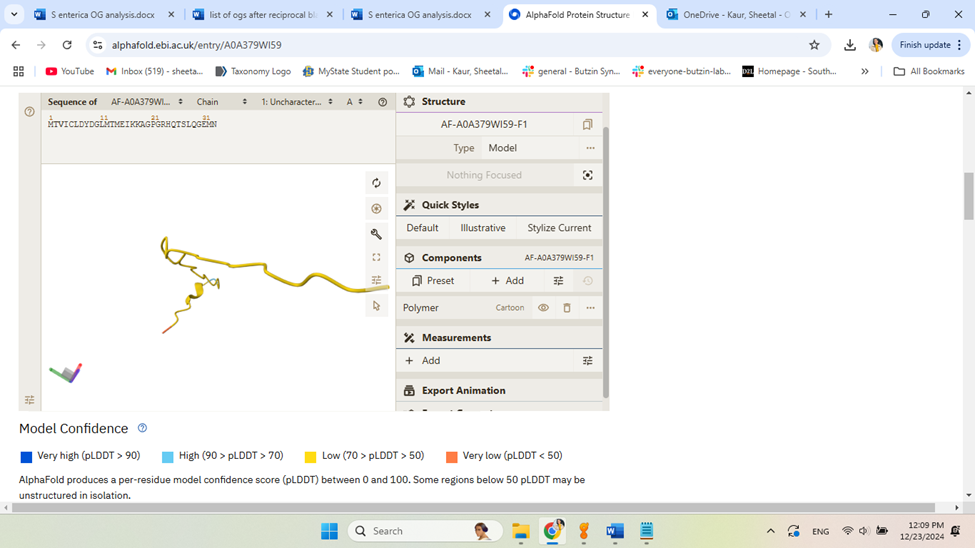 | *S. enterica* | MTVICLDYDGLMTMEIKKAGPGRHQTSLQGEMN |
| 5 | 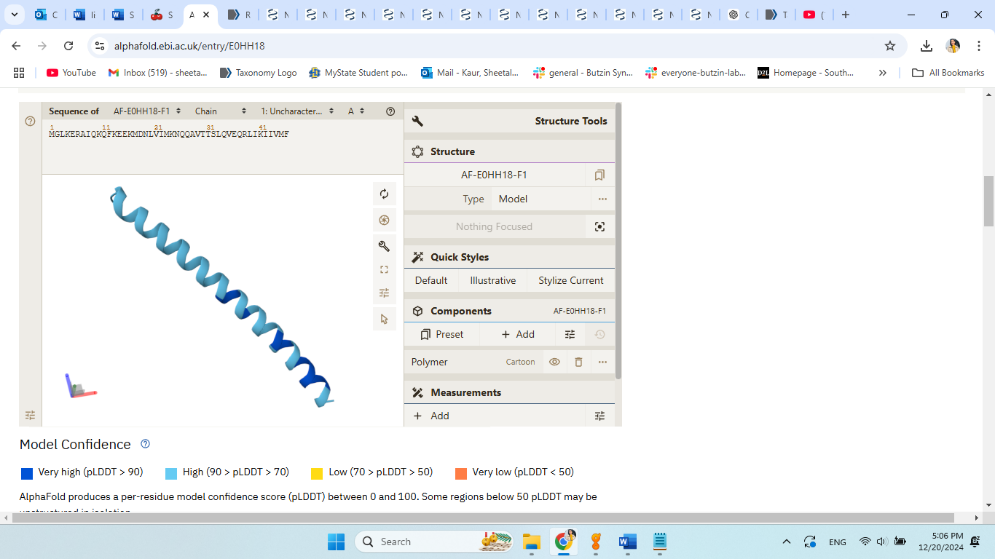 | *E. faecalis* | MDNLVIMKNQQAVTTSLQVEQRLIKIIVMF |
| 6 | 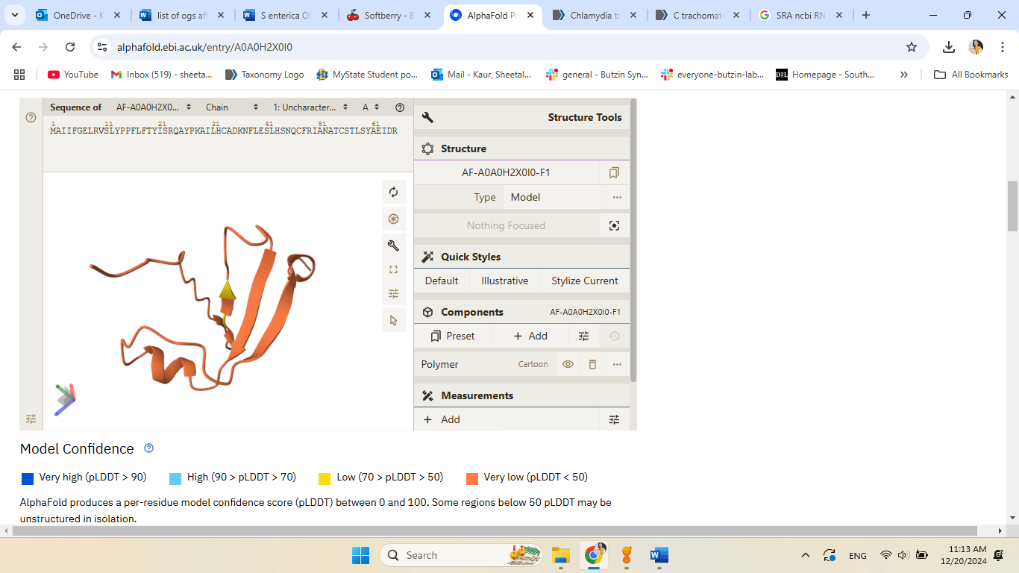 | *C. trachomatis* | MAIIFGELRVSLYPPFLFTYISRQAYPKAILHCADKNFLESLHSNQCFRIANATCSTLSYAEIDR |
| 7 | 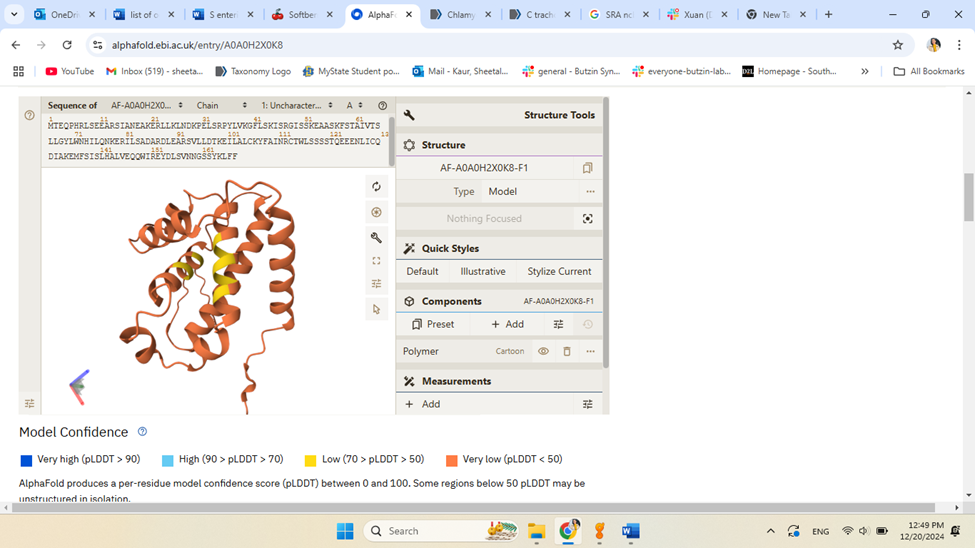 | *C. trachomatis* | MTEQPHRLSEEARSIANEAKERLLKLNDKPELSRPYLVKGFLSKISRGISSKEAASKFSTAIVTSLLGYLWNHILQNKERILSADARDLEARSVLLDTKEILALCKYFAINRCTWLSSSSTQEEENLICQDIAKEMFSISLHALVEQQWIREYDLSVNNGSSYKLFF |
| 8 | **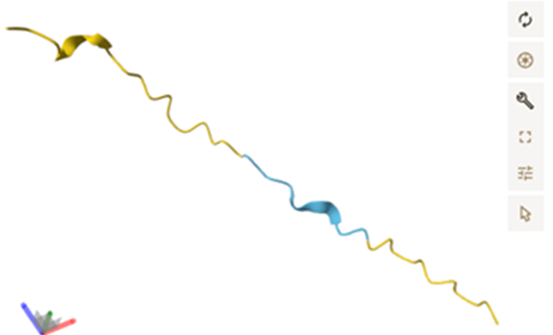** | *C. trachomatis* | MLAKKQDQKNLFLKPPLLNLNYKKTNQFNFSKKELNINCCKKQYLF |
| 9 | 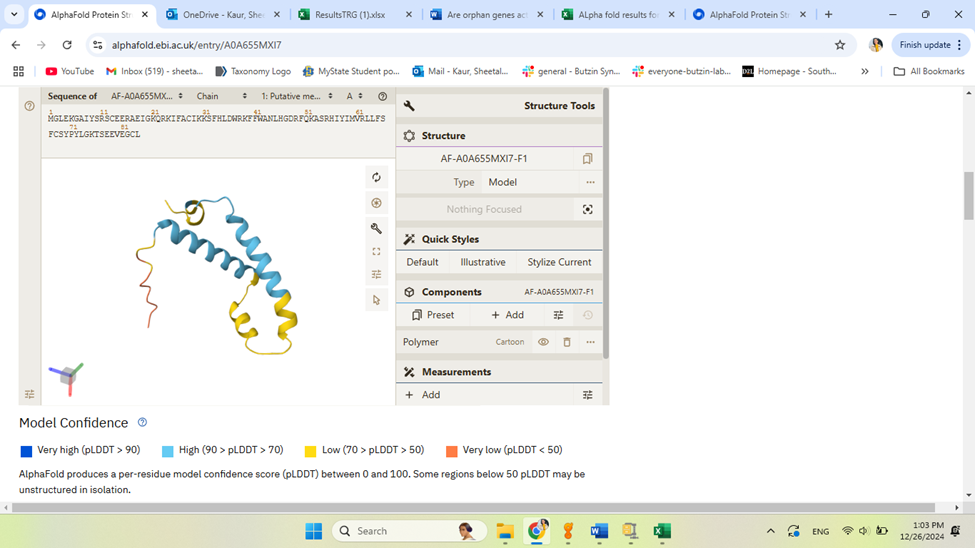 | *C. trachomatis* | MGLEKGAIYSRSCEERAEIGKQRKIFACIKKSFHLDWRKFFWANLHGDRFQKASRHIYIMVRLLFSFCSYPYLGKTSEEVEGCL |
| 10 | 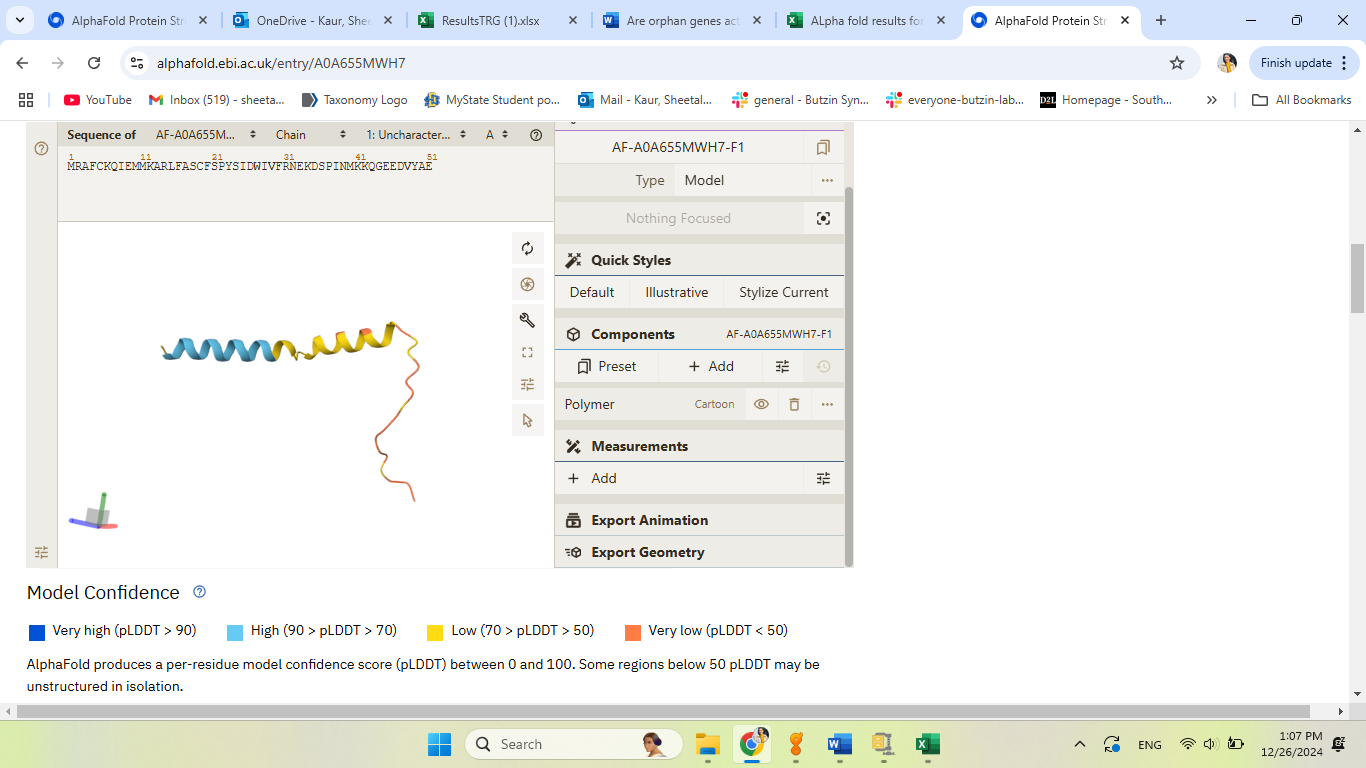 | *C. trachomatis* | MRAFCKQIEMMKARLFASCFSPYSIDWIVFRNEKDSPINMKKQGEEDVYAE |
| 11 | 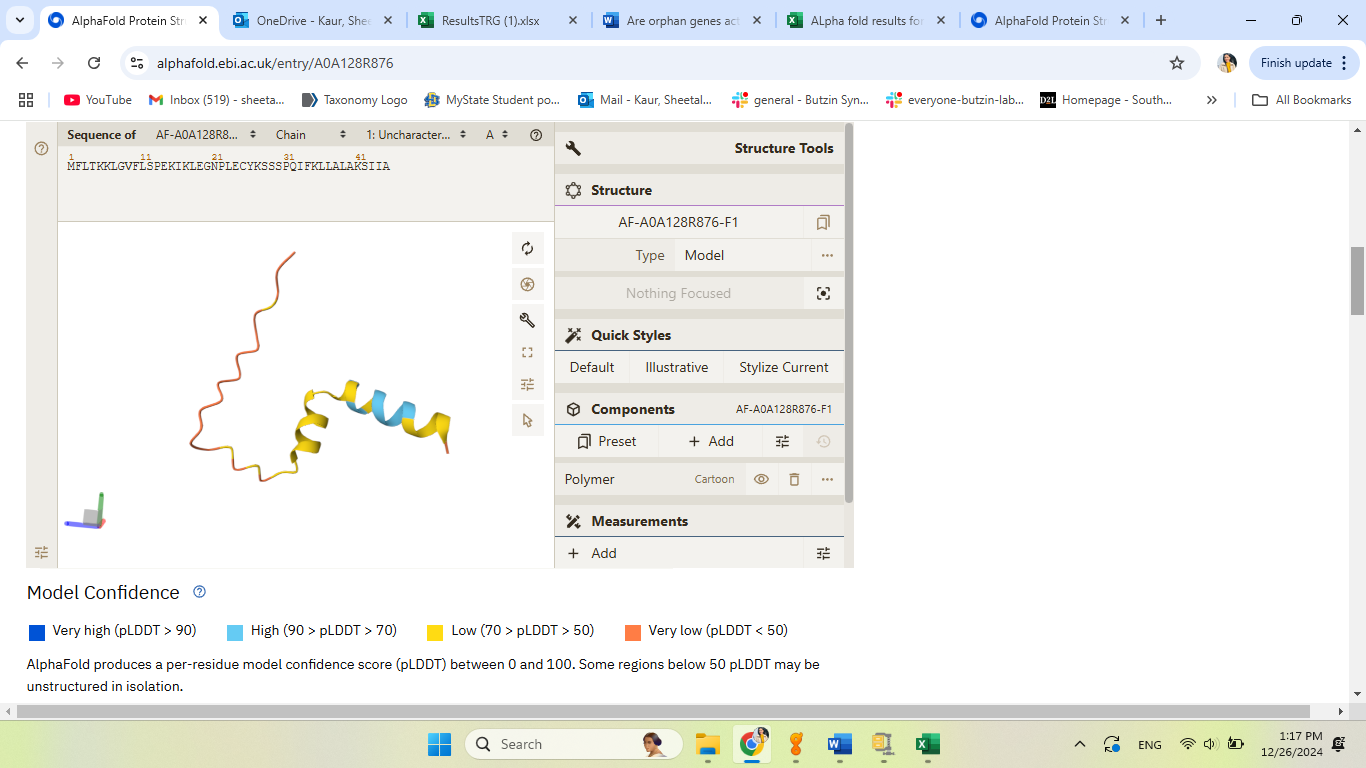 | *L. pneumophila* | MFLTKKLGVFLSPEKIKLEGNPLECYKSSSPQIFKLLALAKSIIA |
| 12 | 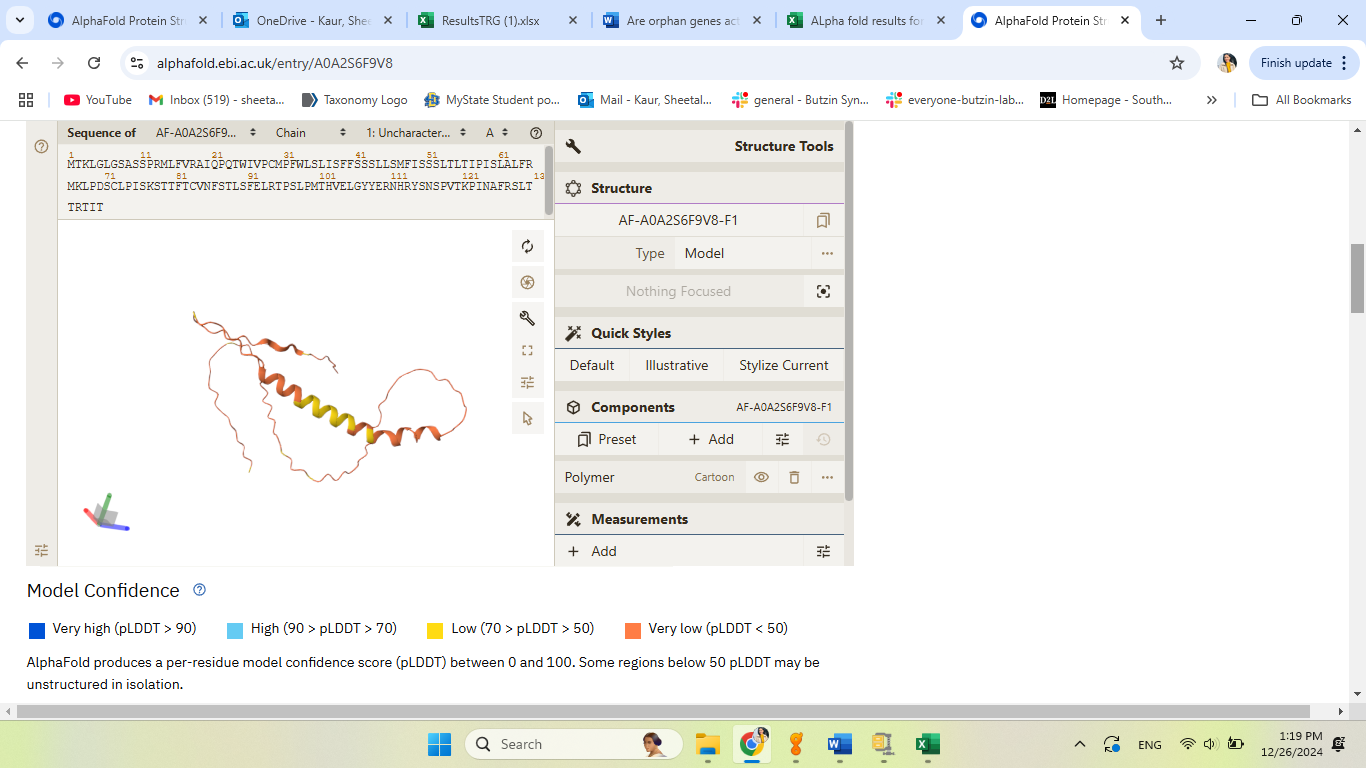 | *L. pneumophila* | MTKLGLGSASSPRMLFVRAIQPQTWIVPCMPFWLSLISFFSSSLLSMFISSSLTLTIPISLALFRMKLPDSCLPISKSTTFTCVNFSTLSFELRTPSLPMTHVELGYYERNHRYSNSPVTKPINAFRSLTTRTIT |
| 13 | 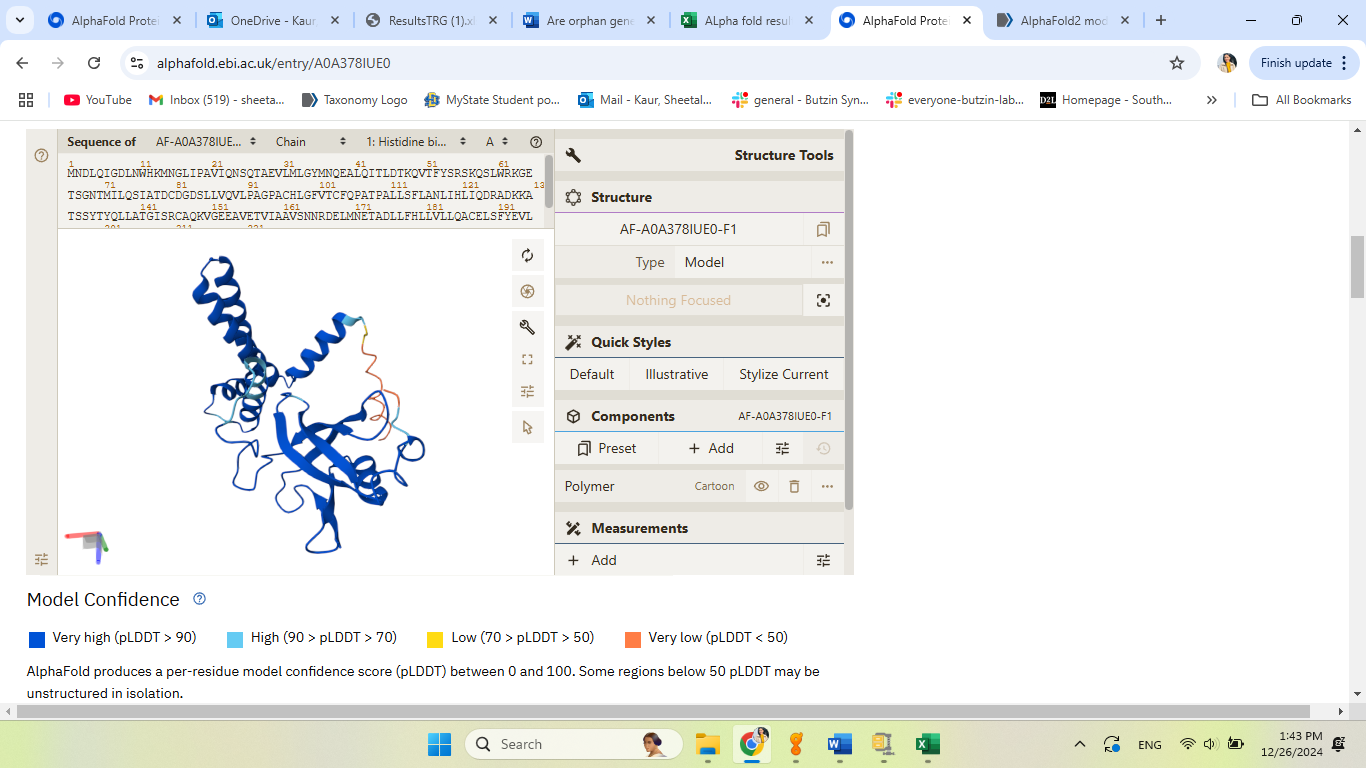 | *L. pneumophila* | MANTRTSRFFYKLDFFEGLHAPFRNRTGLTKNSKVEAKNVFNEGA |
| 14 | 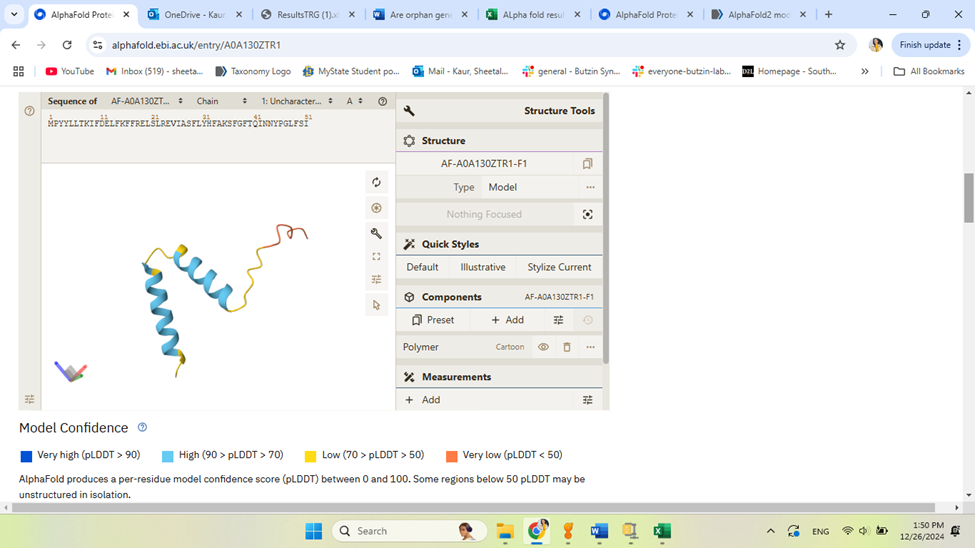 | *L. pneumophila* | MPYYLLTKIFDELFKFFRELSLREVIASFLYHFTKSFGFTQIKNYPGPFSILKRPGSYDR |
| 15 | 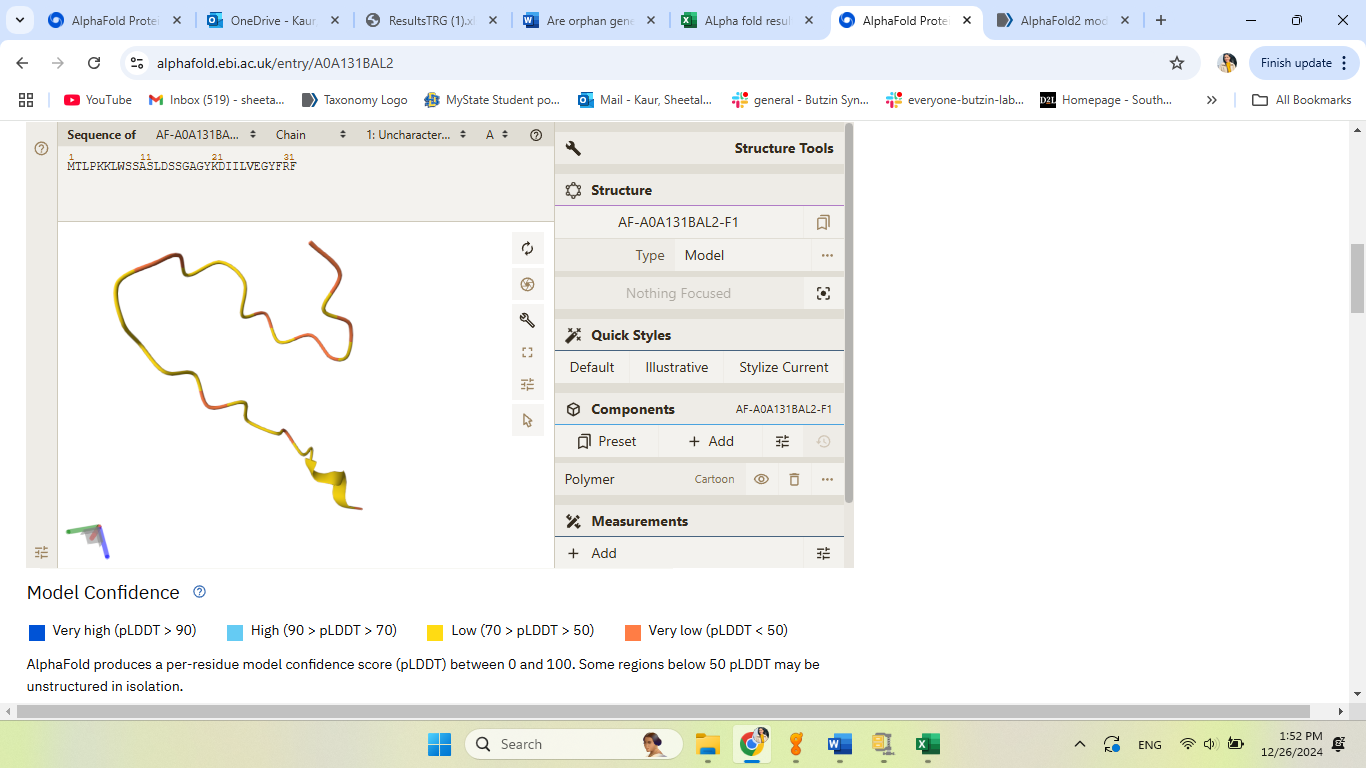 | *L. pneumophila* | MTLPKKLWSSASLDSSGAGYKDIILVEGYFRF |
| 16 | 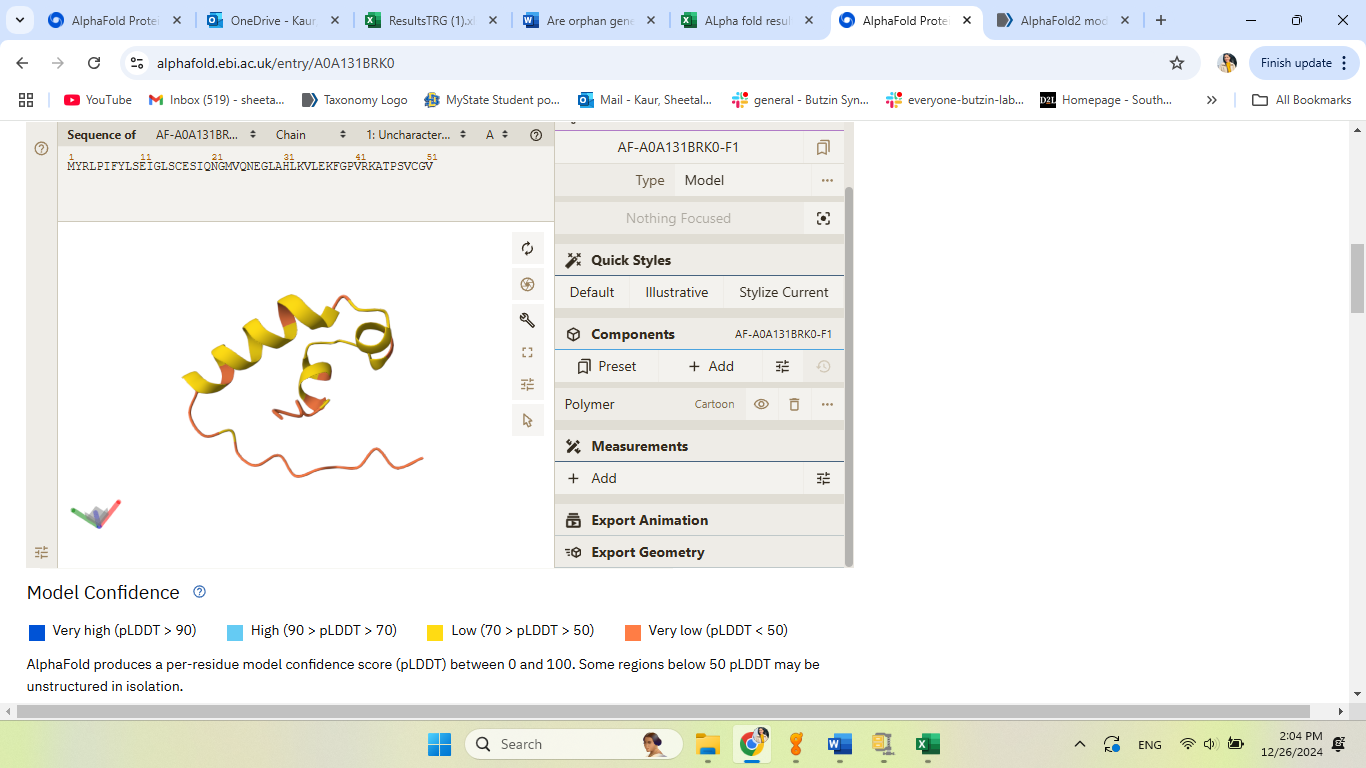 | *L. pneumophila* | MYRLPIFYLSEIGLSCESIQNEMVQNEGLAHLKVSEKFGSVRKATPFVCGE |
| 17 | 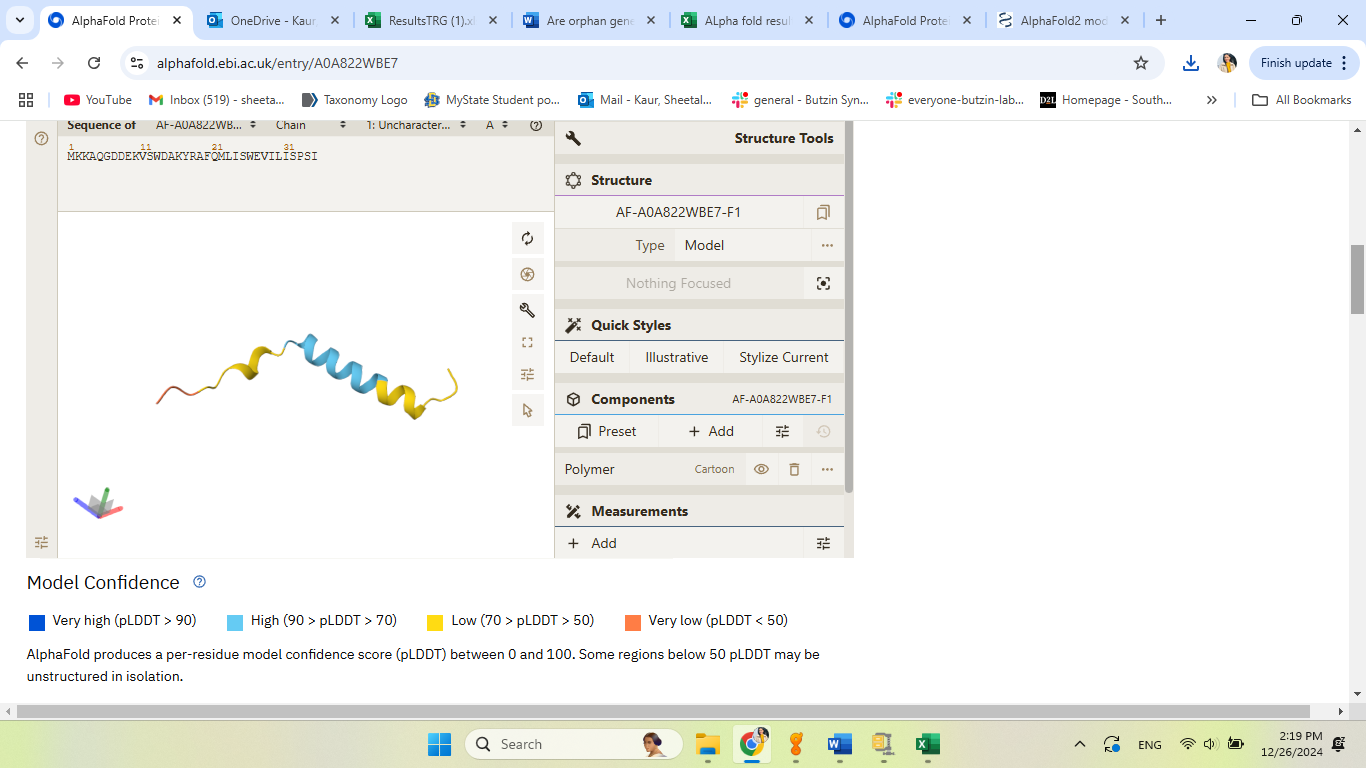 | *L. pneumophila* | MRKAQGDDEKASWDAKYGAFQMLISWEVILISPST |
| 18 | 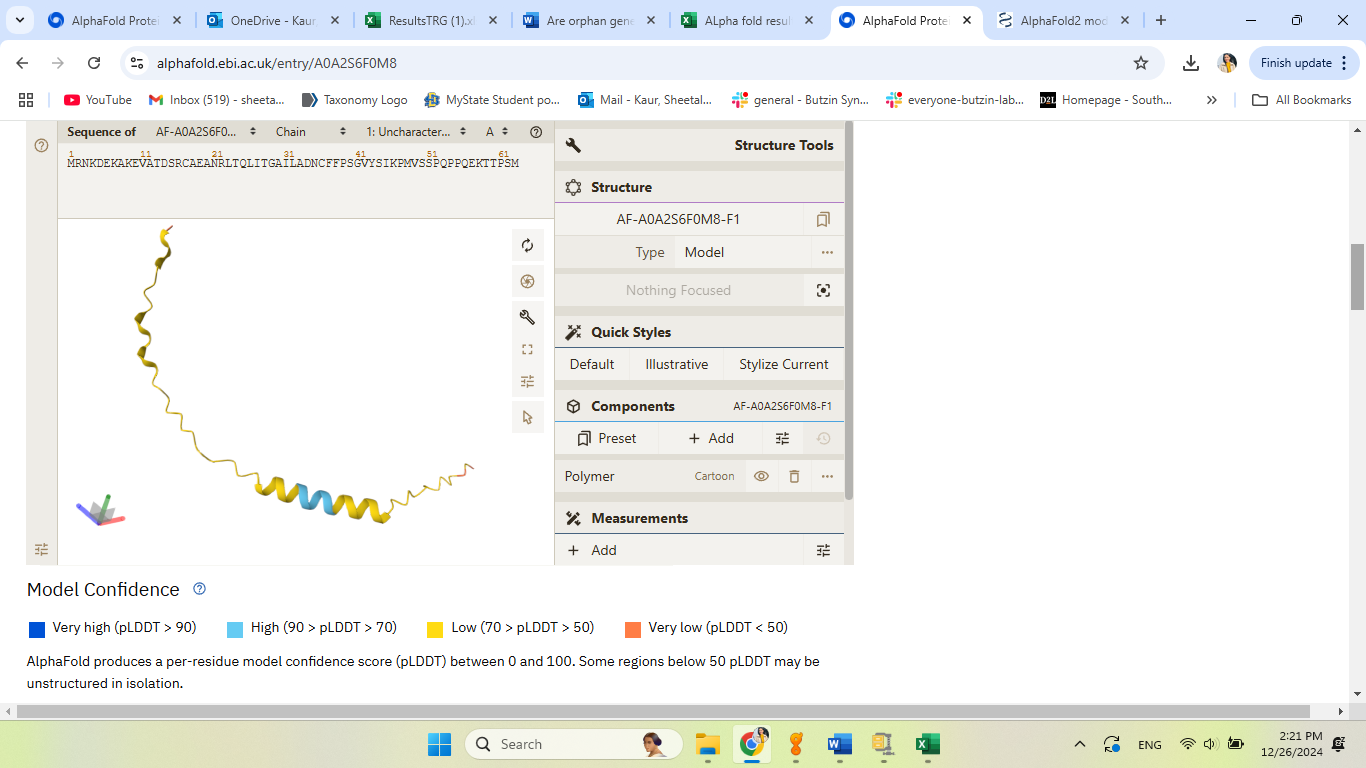 | *L. pneumophila* | MRNKDEKAKEVATDSRCAEANRLTQLITGAILADNCFFPSGVYSIKPMVSSPQPPQEKTTPSM |
| 19 | 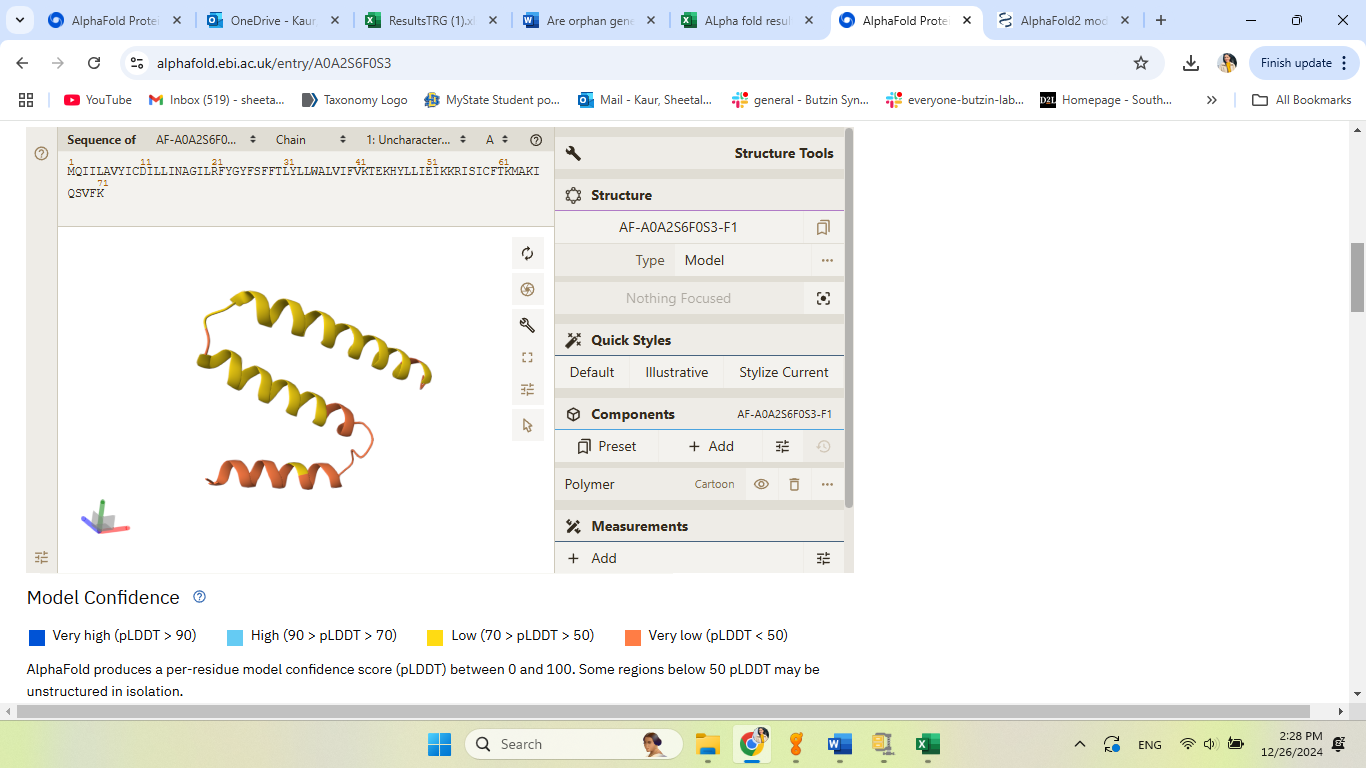 | *L. pneumophila* | MQIILAVYICDILLINAGILRFYGYFSFFTLYLLWALVIFVKTEKHYLLIEIKKRISICFTKMAKIQSVFK |
| 20 | 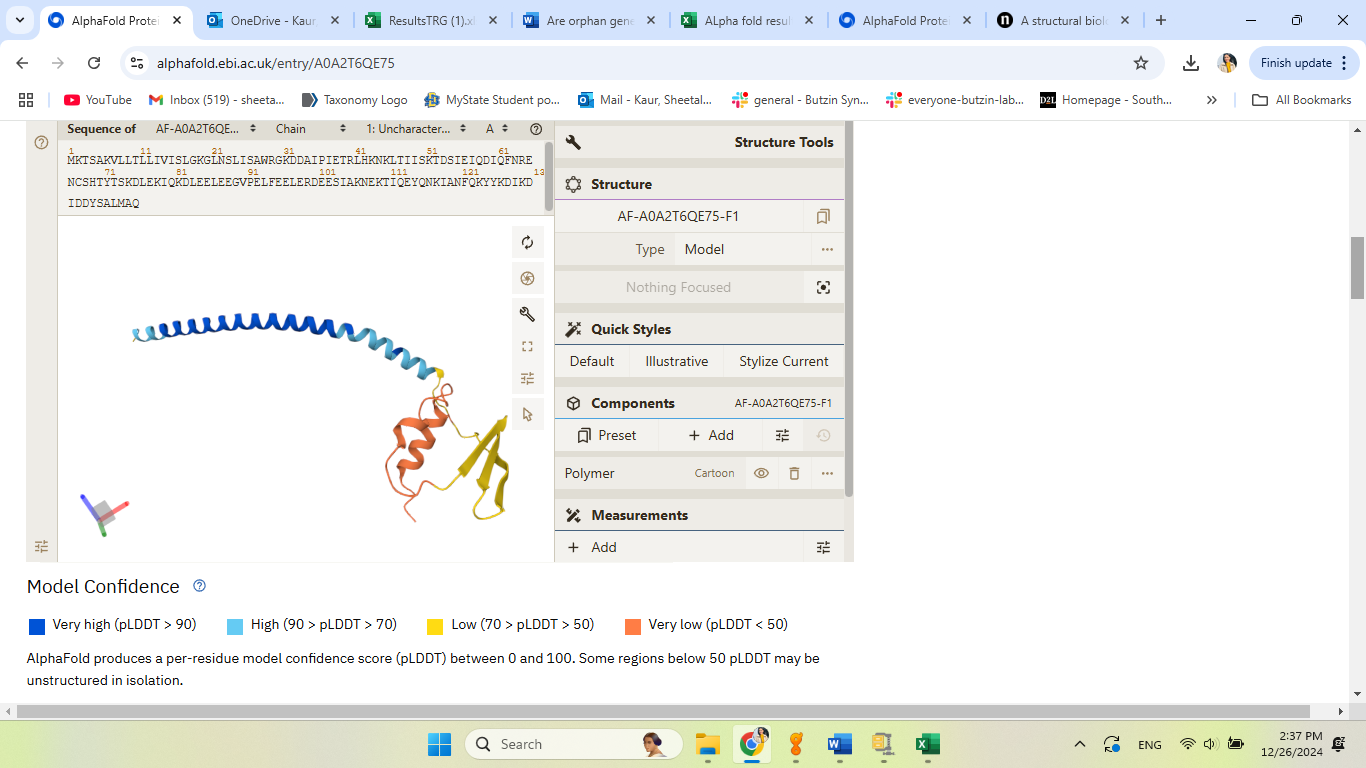 | *H. pylori* | MSLGKGLISLIKDDTIPIETRLHENKLTIISKTDNIEIQNIEFNRGNCSDTAYNKGSERIEKESEEELAREYFYYELESDRNFIAKNEKTI |
| 21 | 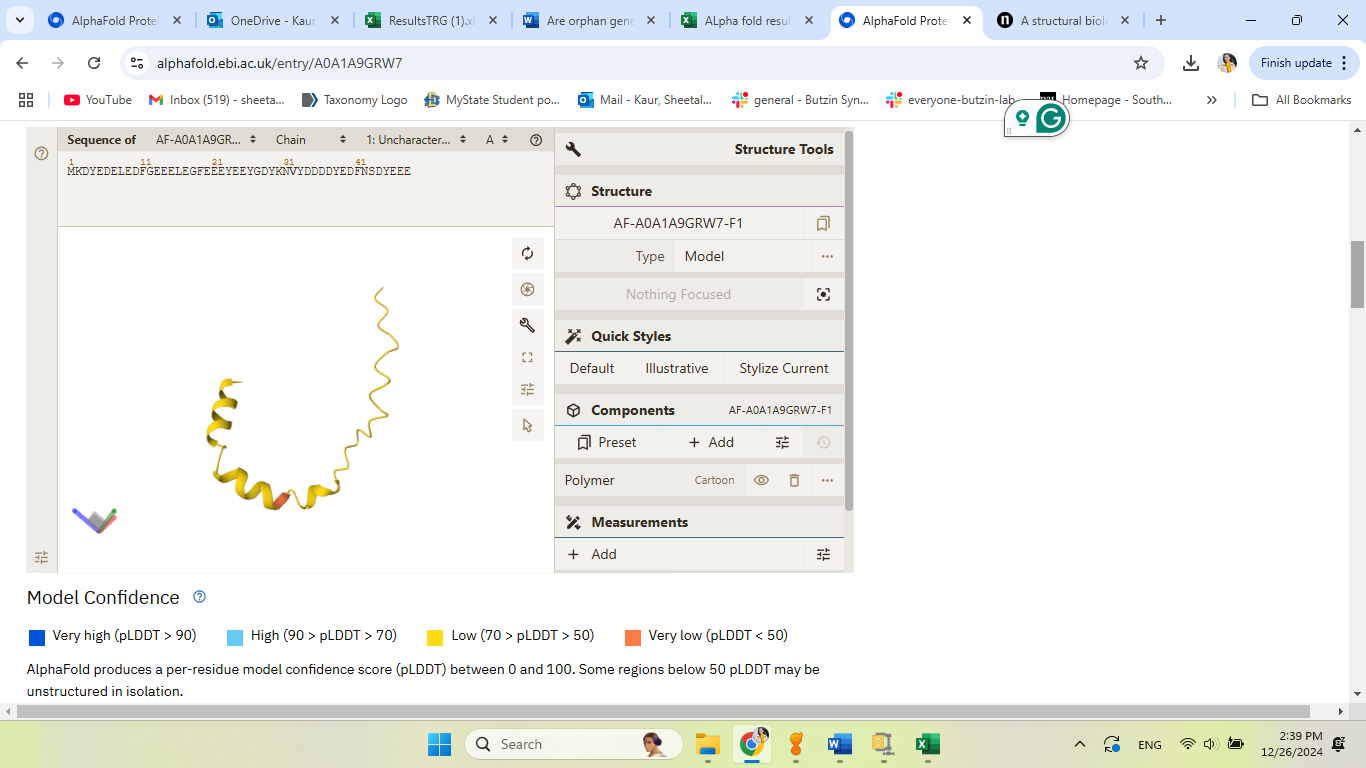 | *H. pylori* | MKDYEDELEDFGEEELEGFEEEYEEYGDYKNVYDDDDYEDFNSDYEEE |
| 22 | 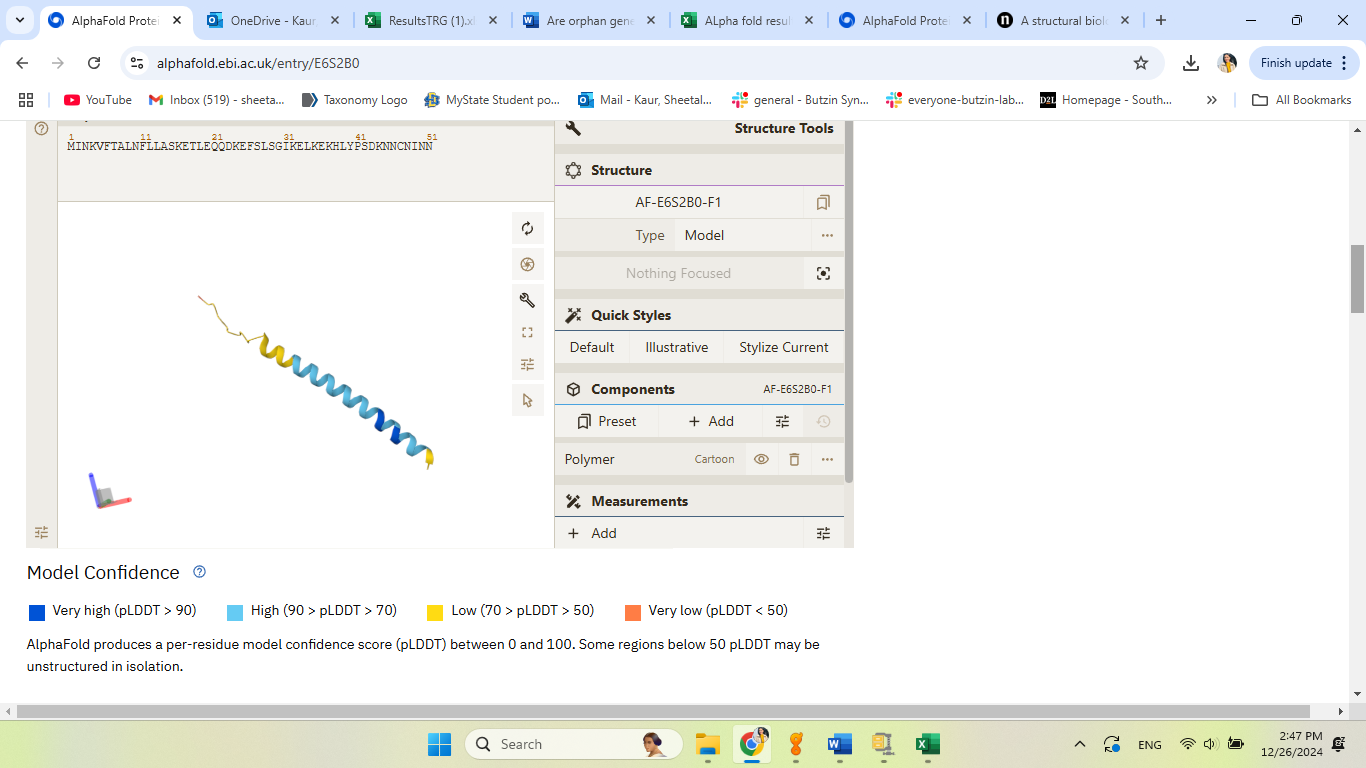 | *H. pylori* | MIDKVFTALNFLLASKETLEQQDKELKEKHLYPSDKNNCNTNNQTTAIPTKICLFTAKILKNQ |
| 23 | 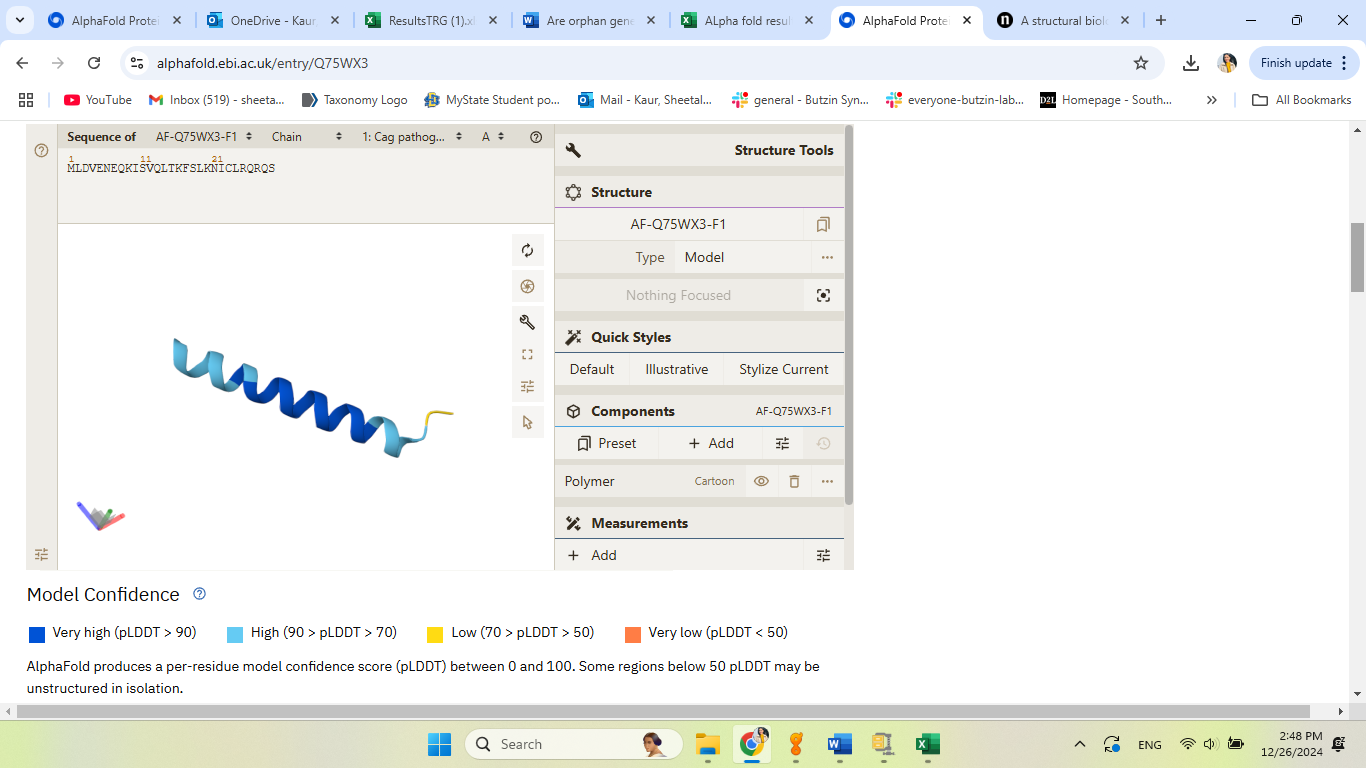 | *H. pylori* | MLDVENEQKISVQLTKFSLKNICLGQRQD |
| 24 | 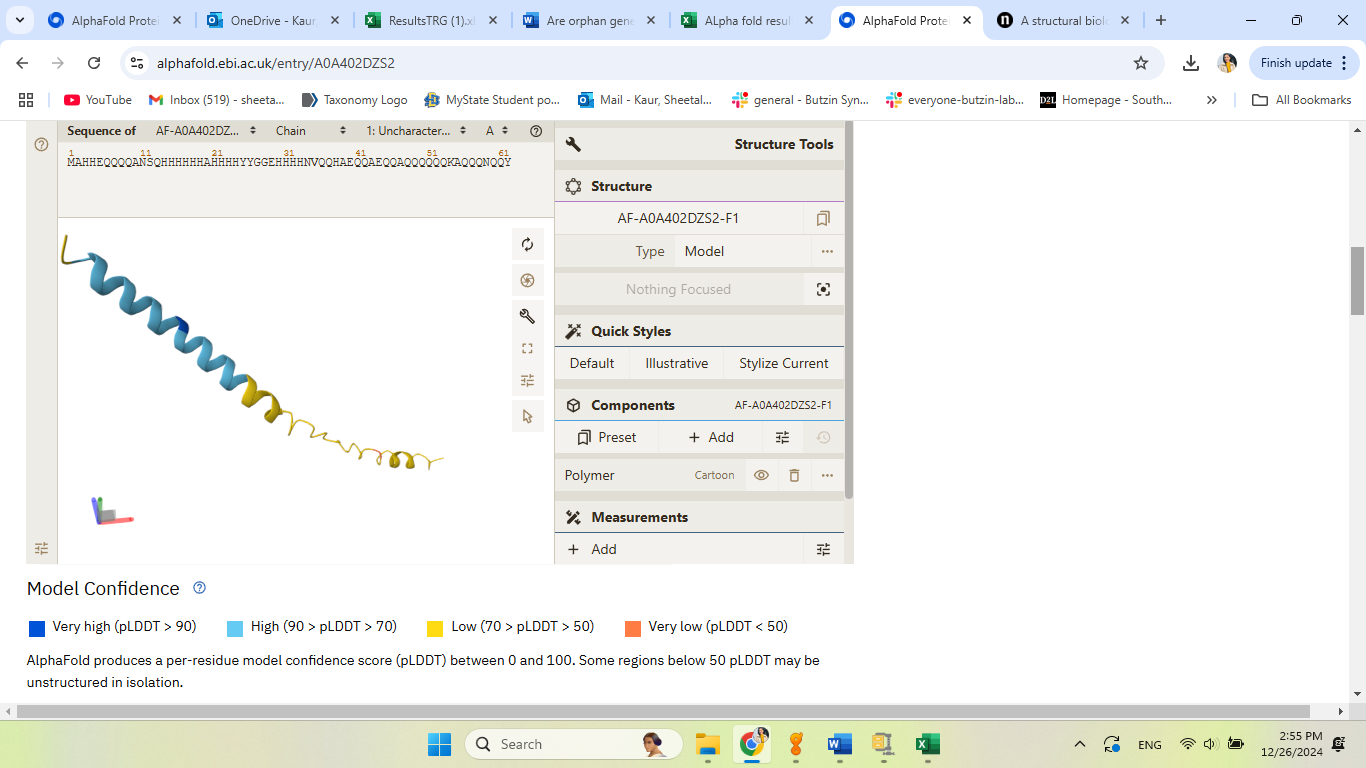 | *H. pylori* | MAHHEQQQQANSQHHHHHHAHHHHYYGGEHHHHNVQQHAEQQAEQQAQQQQQQKAQQQNQQY |
| 25 | 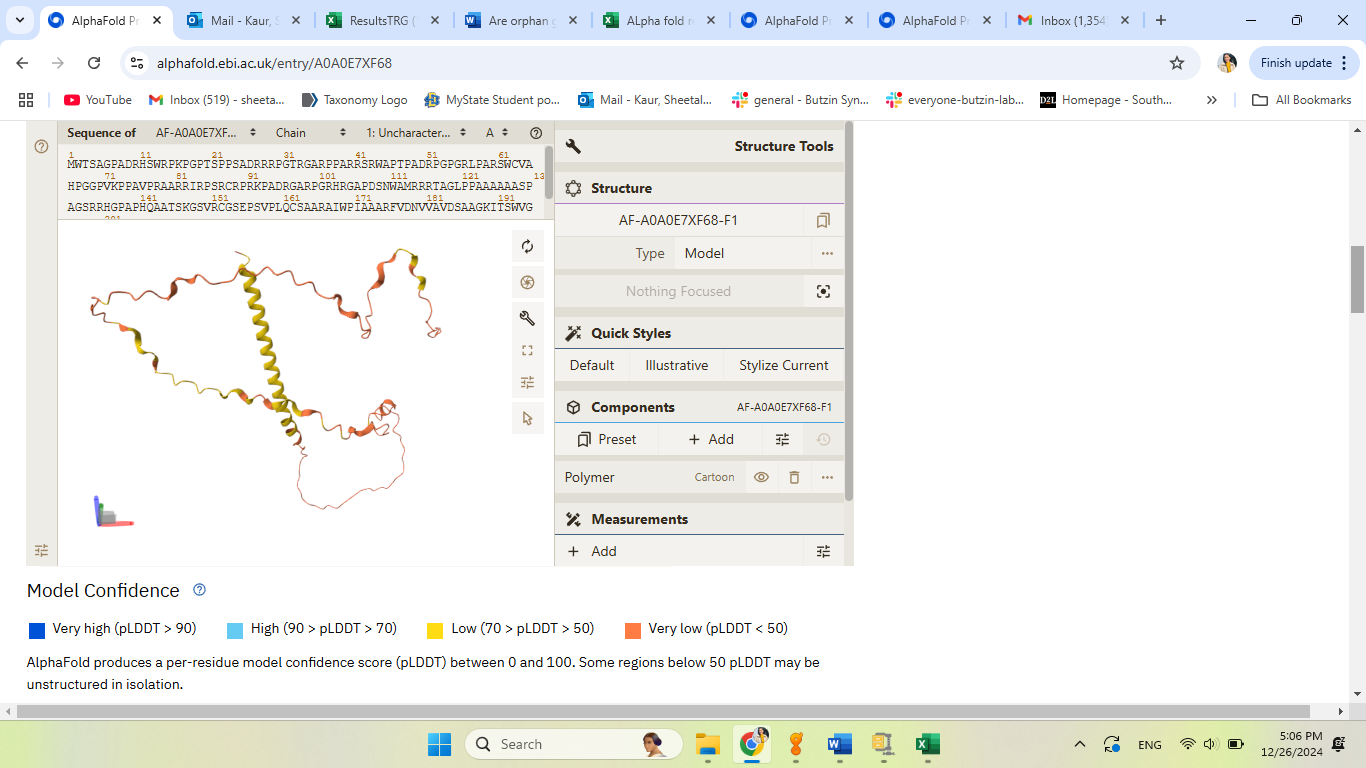 | *M. tuberculosis* | MWTSAGPADRHSWRPKPGPTSPPSADRRRPGTRGARPPARRSRWAPTPADRPGPGRLPARSWCVAHPGGPVKPPAVPRAARRIRPSRCRPRKPADRGARPGRHRGAPDSNWAMRRRTAGLPPAAAAAASPAGSRRHGPAPHQAATSKGSVRCGSEPSVPLQCSAARAIWPIAAARFVDNVVAVDSAAGKITSWVGVNYSAQLASAG |
| 26 | 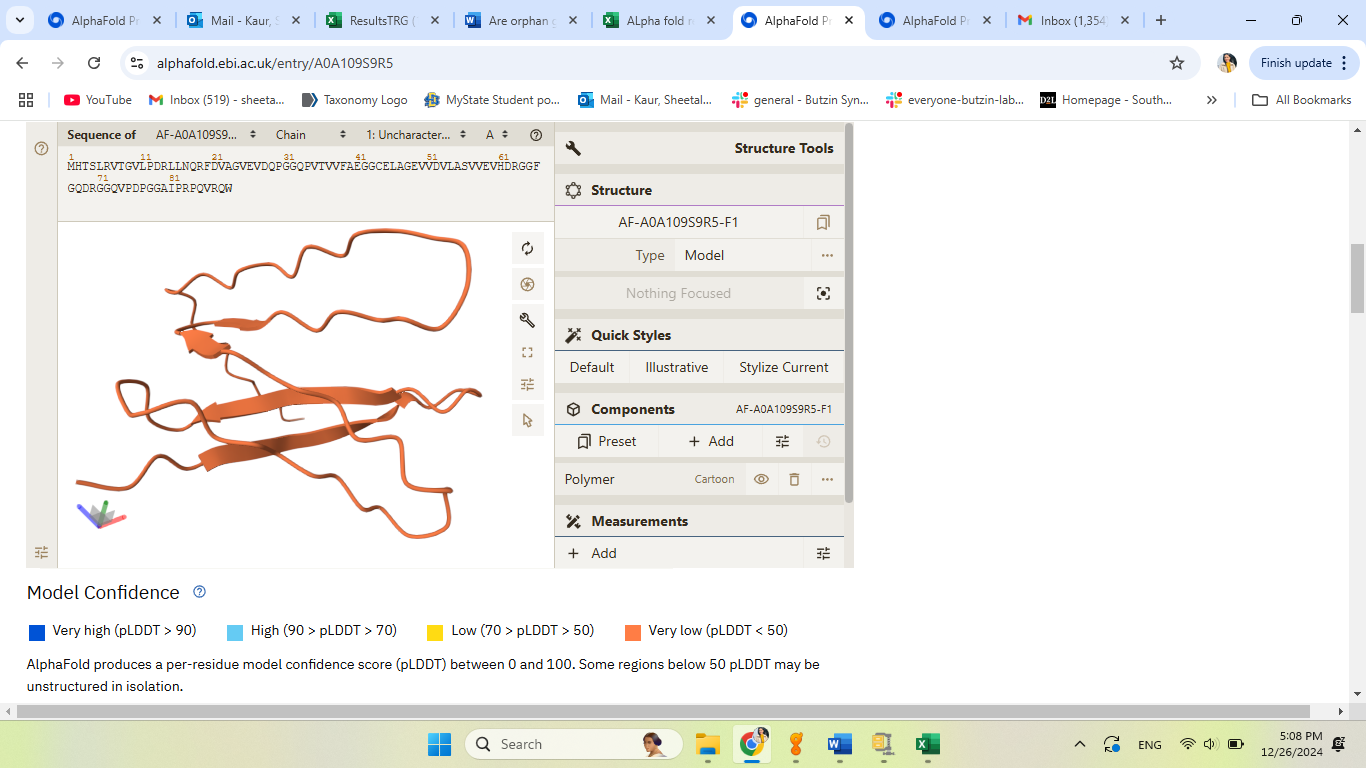 | *M. tuberculosis* | MHTSLRVTGVLPDRLLNQRFDVAGVEVDQPGGQPVTVVFAEGGCELAGEVVDVLASVVEVHDRGGFGQDRGGQVPDPGGAIPRPQVRQW |
| 27 | 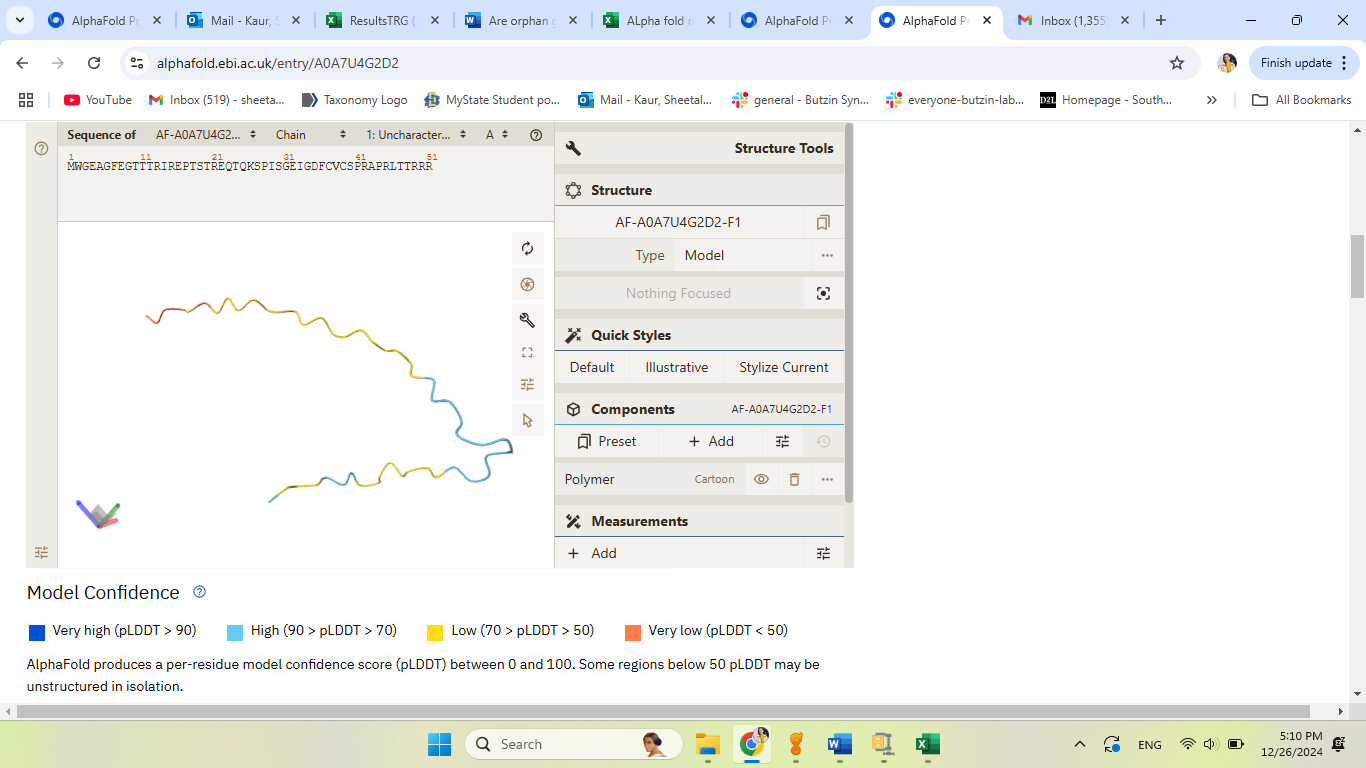 | *M. tuberculosis* | MWGEAGFEGTTTRIREPTSTREQTQKSPISGEIGDFCVCSPRAPRLTTRRR |
| 28 | 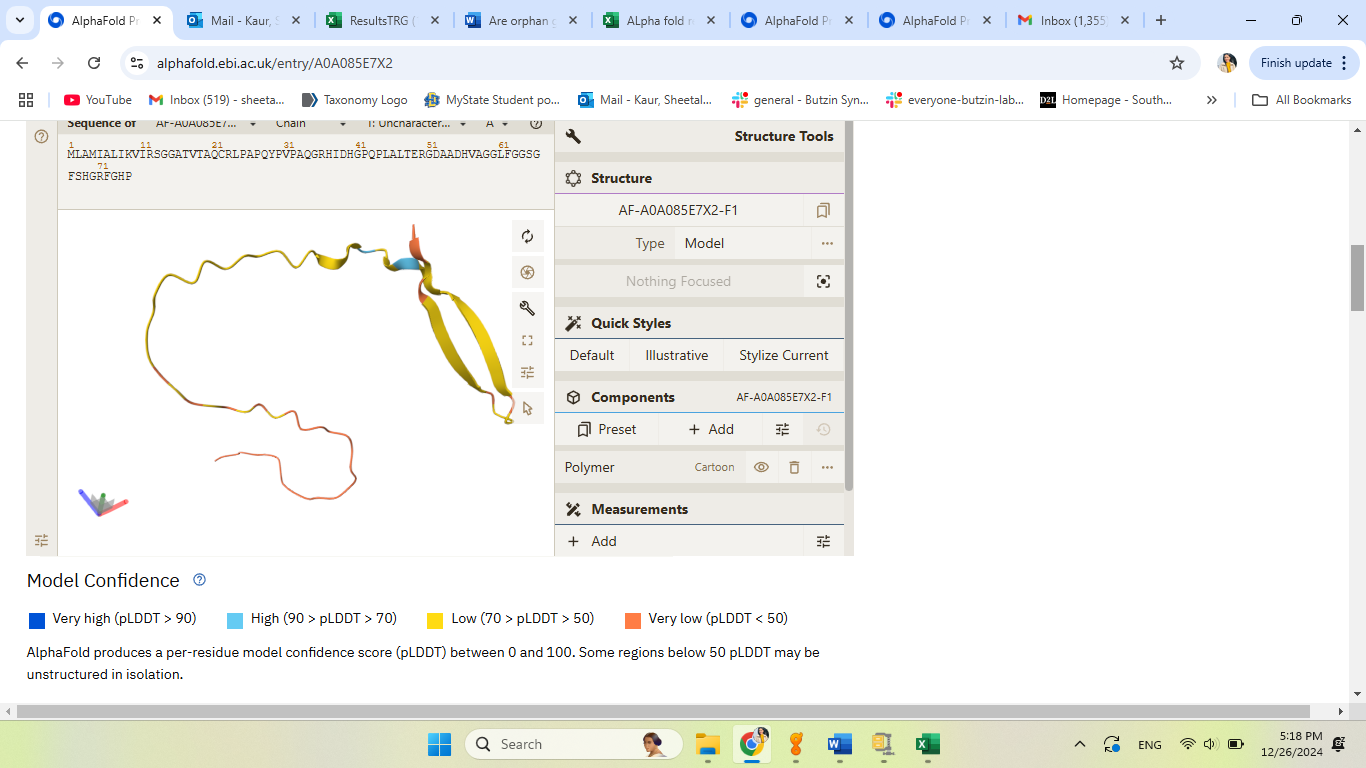 | *M. tuberculosis* | MLAMIALIKVIRSGGATVTAQCRLPAPQYPVPAQGRHIDHGPQPLALTERGDAADHVAGGLFGGSGFSHGRFGHP |
| 29 | 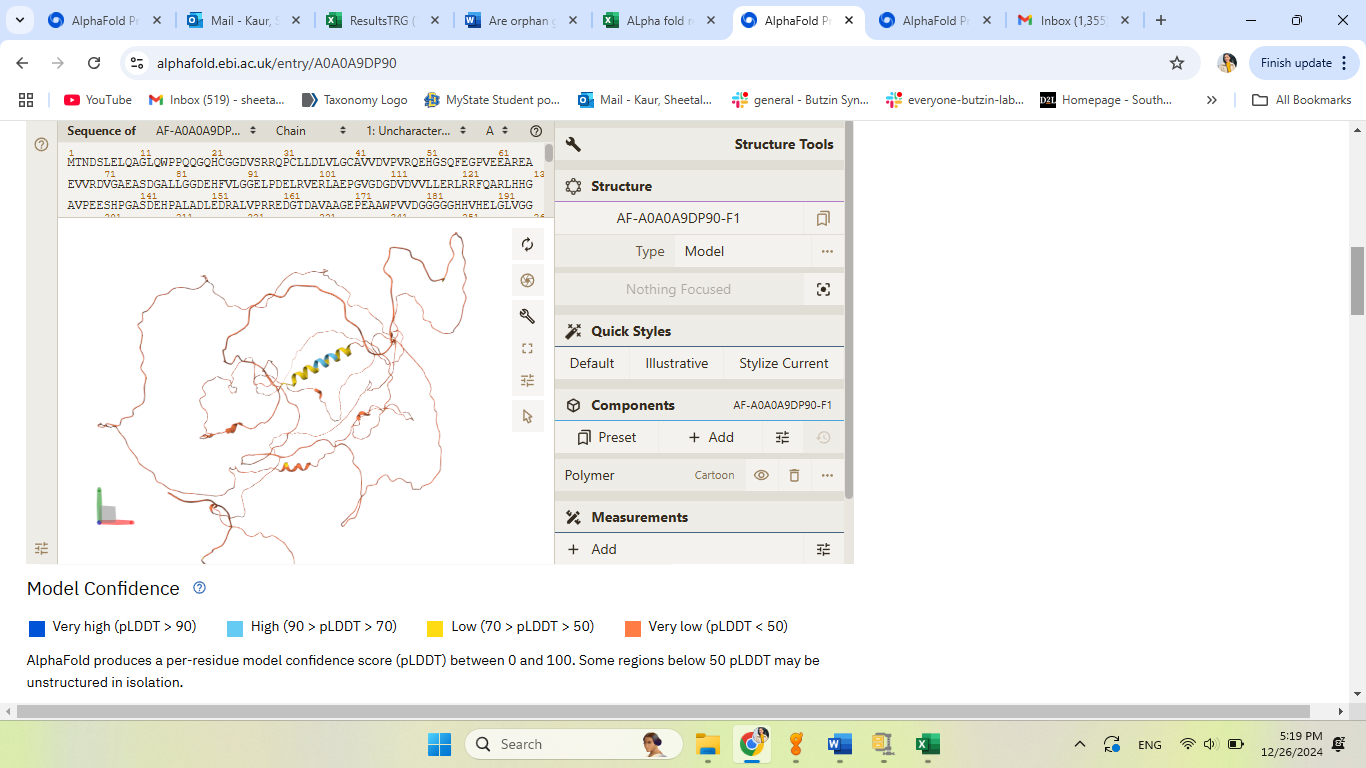 | *M. tuberculosis* | MQCDGQLYHAKSRAEMATGSRHCFYEPPTHLHGQLWQLAFGQCLHIARSADRGN |
| 30 | 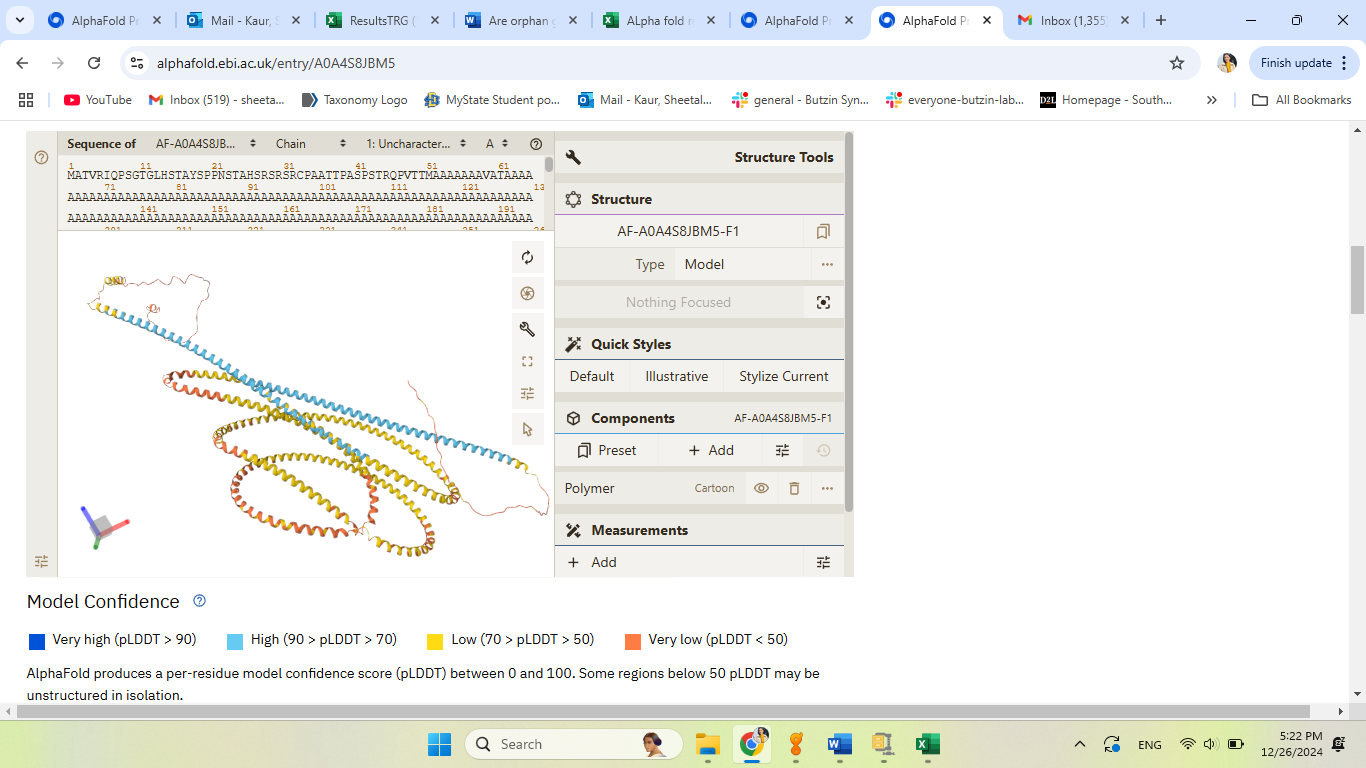 | *M. tuberculosis* | MAPRLGDTAGSPFVVKSGWTTRRRTPGAPSRPLGPSRLDAAAAALAARSAVAAGAAGATGWCLTTVSGATAAAGVSLAAIPAGAAVPTTTAGAAIAAGAARAGATAVAAGATLAAGGAGAASTTGSRGHSGLGVAAIAAGAADTAGPTGAAVAIASRAGVAAVAAIAGRTAAAAGAAVAAGSADPGVAEAAGAAGAAGAAGAPVPAAGAAGAAIAAIAGRTAGAAGAADLAALAAGTAVAAVAAIAASAAAKAADARAAGAAGAAVAAGAPVAAGVVSEWAVAADGPVAAIAAGAALPAGAVGASGQATAAGPGPAAEATVAAVATVTARLRSHVCGVICSVAAVTAVTAGATAAAGAASTGEAAVAAGAARARRAAVPAGTAVAAAGAAVAAHAGVAAVAAAAAVAAVAAARPGTAVAAHAGDAAVAAVAAVAGAVAVAAVAGVAGVAASAAVAAGAAAAAPSAAGATVSTTTTVAASAAGAIAGITVAAVTAIATCAGVAAVAAAAAVGSTLPAGPTTASVTALSTGAGGDRATADGAVAAGAAGTAVAAGATLTPGPATGAALAAGATLSGGAAVTGGPRGARGAAGDIAAVAAGPAGTAGTTLTTGTAAGAALATLSAVAGRPAGAAGASGAAGAAAAAVASGAAGAAAAAVGS |
| 31 | 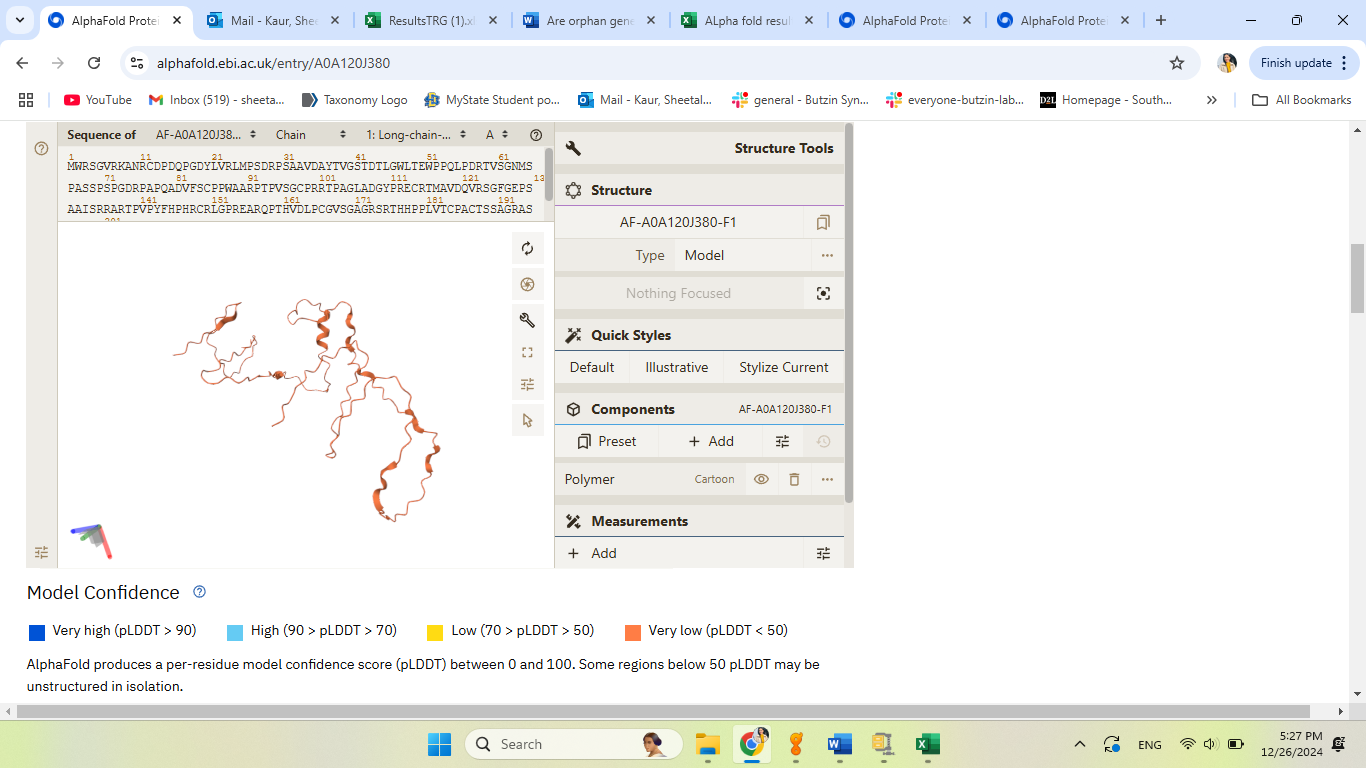 | *M. tuberculosis* | MWRSGVRKANRCDPDQPGDYLVRLMPSDRPSAAVDAYTVGSTDTLGWLTEWPPQLPDRTVSGNMSPASSPSPGDRPAPQADVFSCPPWAARPTPVSGCPRRTPAGLADGYPRECRTMAVDQVRSGFGEPSAAISRRARTPVPYFHPHRCRLGPREARQPTHVDLPCGVSGAGRSRTHHPPLVTCPACTSSAGRASVCSLAQR |
| 32 | 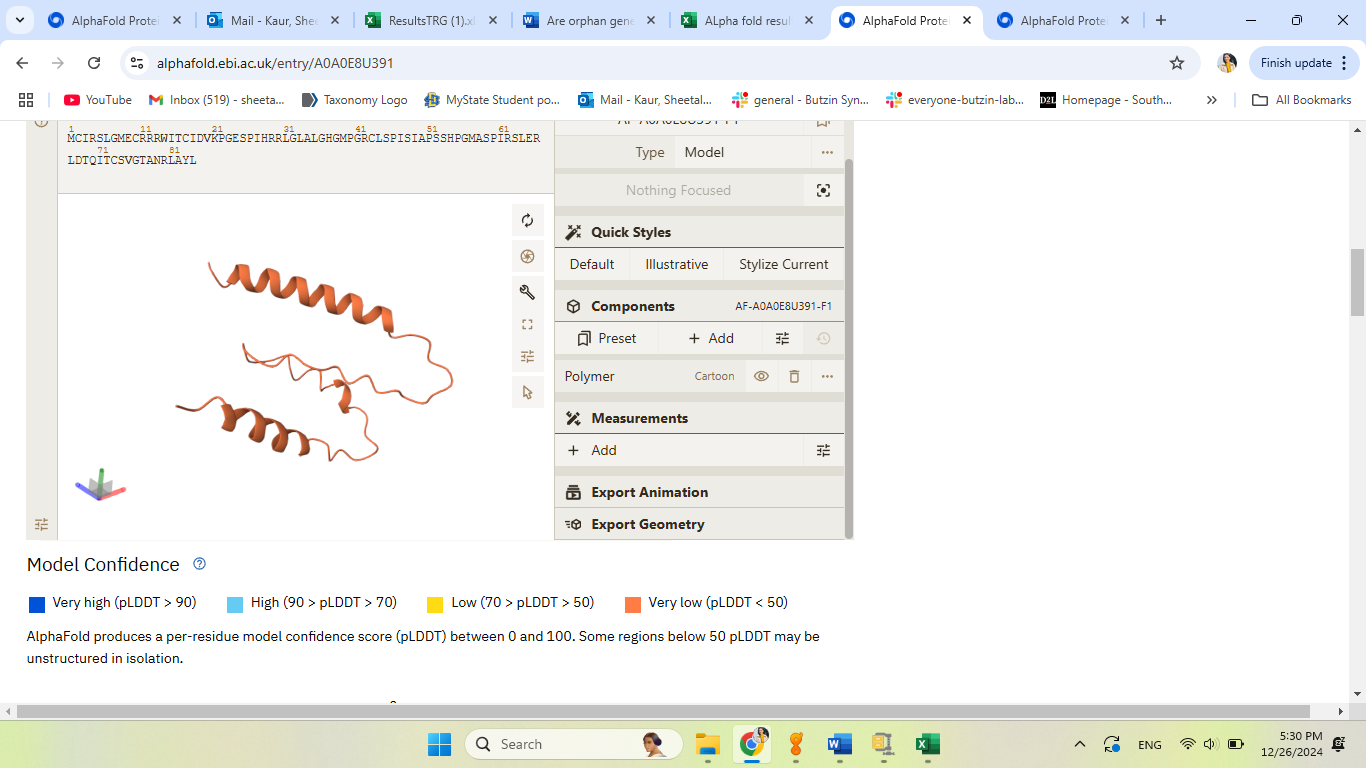 | *M. tuberculosis* | MCIRSLGMECRRRWITCIDVKPGESPIHRRLGLALGHGMPGRCLSPISIAPSSHPGMASPIRSLERLDTQITCSVGTANRLAYL |
| 33 | 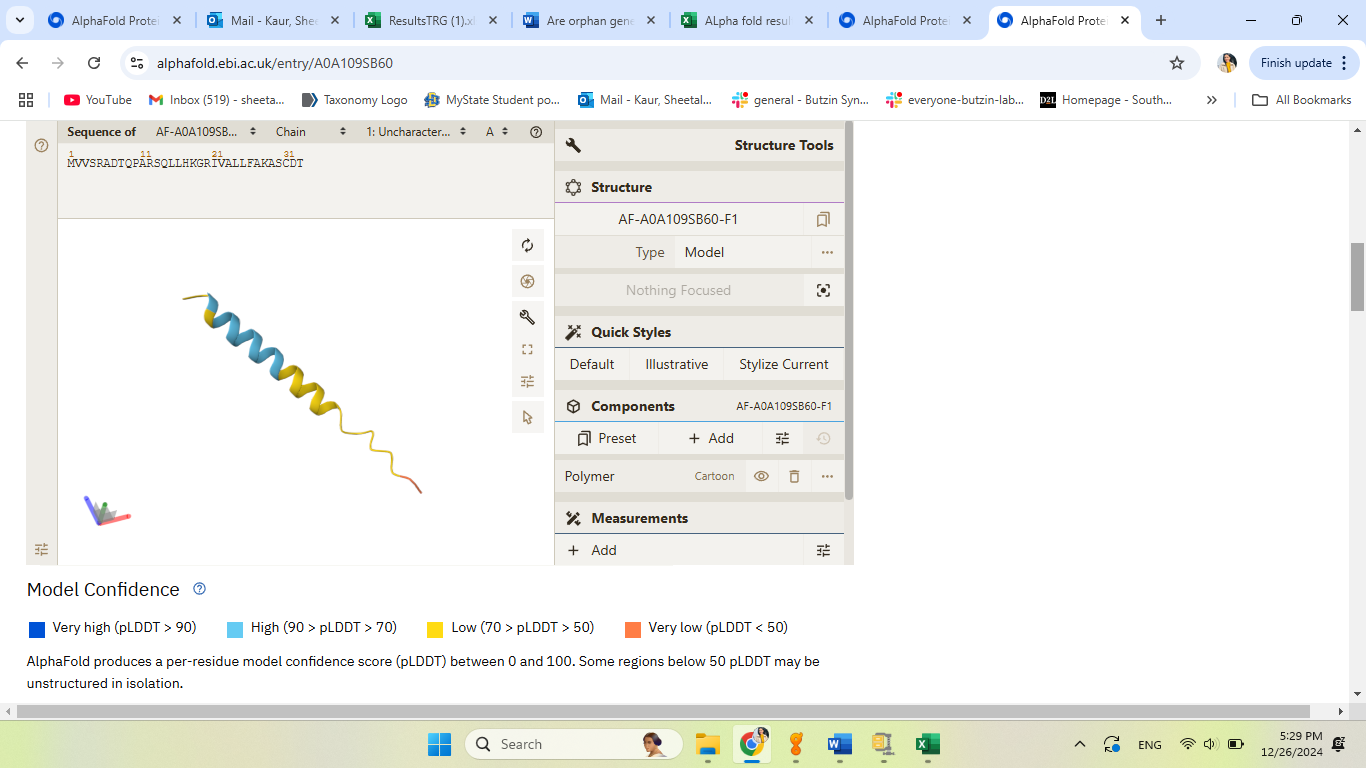 | *M. tuberculosis* | MVVSRADTQPARSQLLHKGRIVALLFAKASCDT |
| 34 | 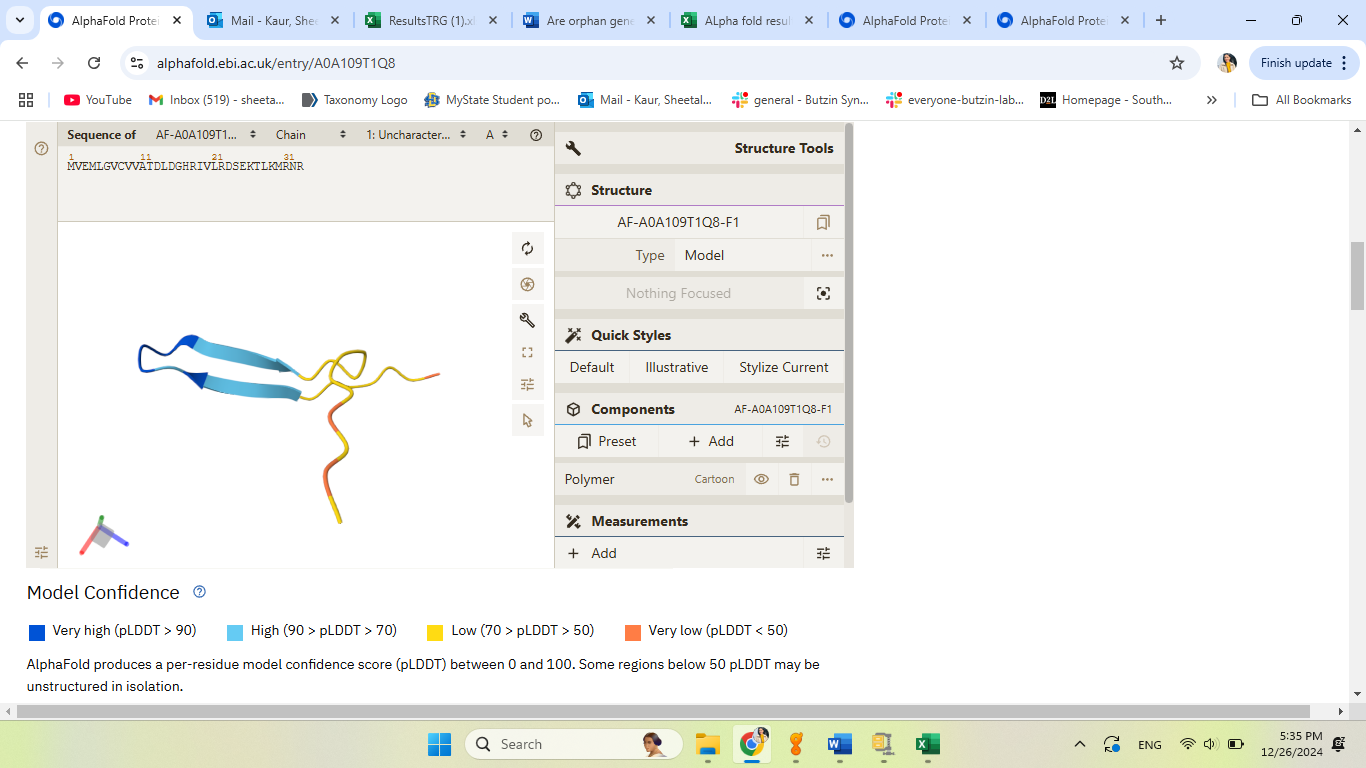 | *M. tuberculosis* | MVEMLGVCVVATDLDGHRIVLRDSEKTLKMRNR |
| 35 | 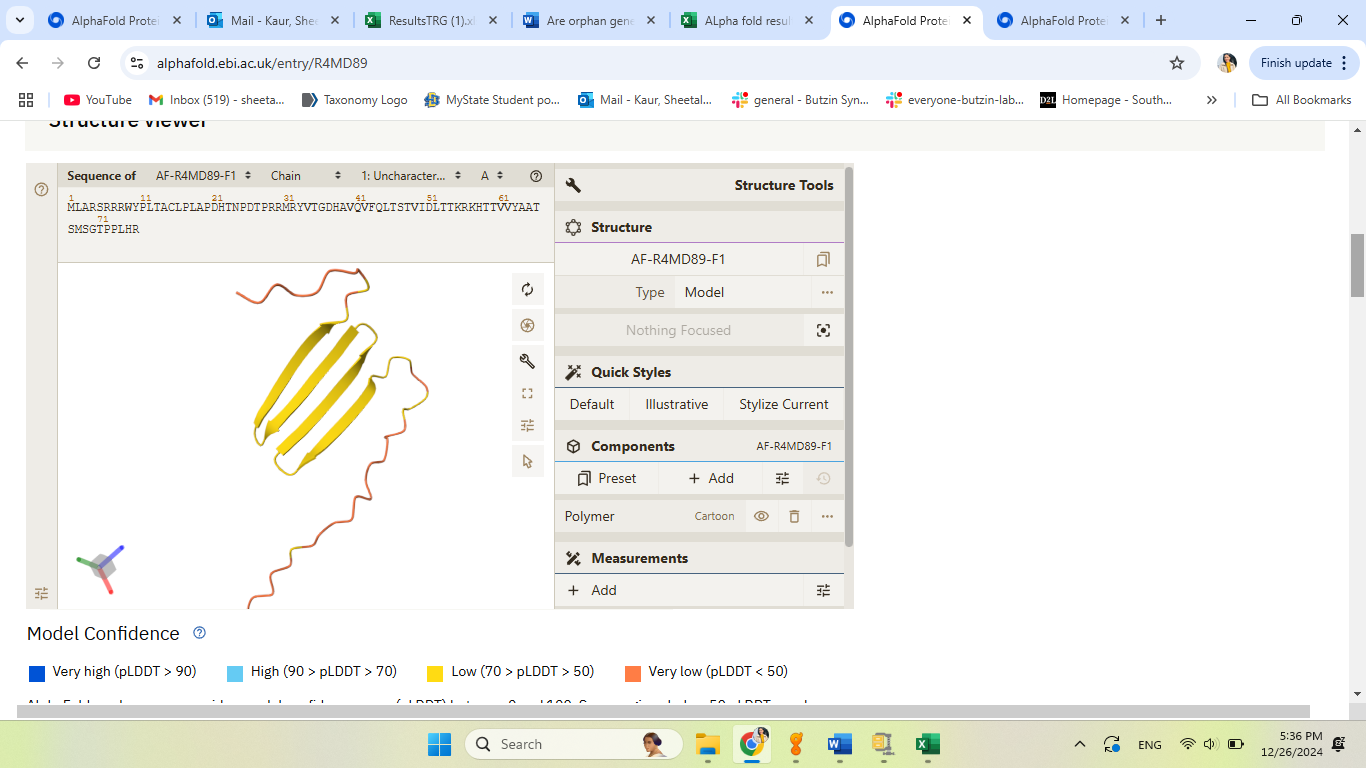 | *M. tuberculosis* | MRYVTGDHAVQVFQLTSTVIDLTTKRKHTTVVYAATSMSGTPPLHR |
| 36 | 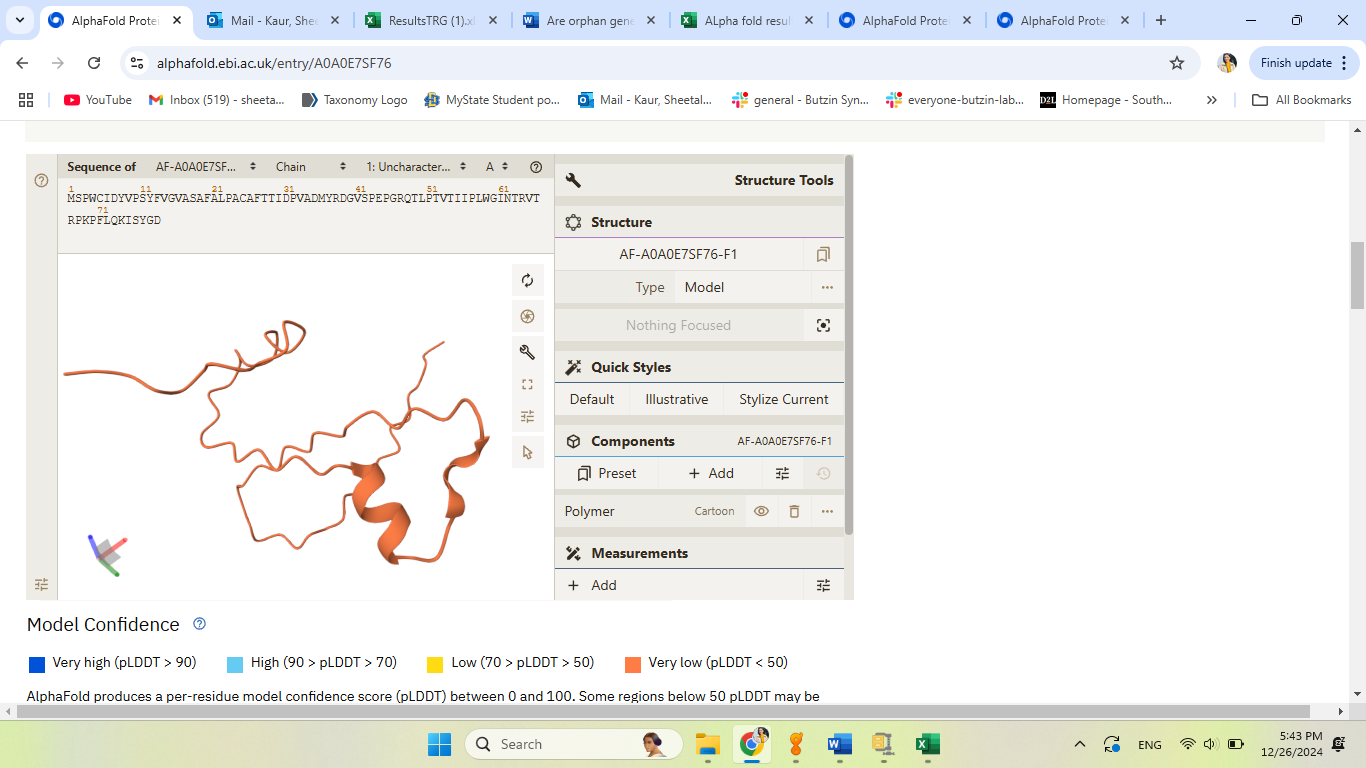 | *M. tuberculosis* | MSPWCIDYVPSYFVGVASAFALPACAFTTIDPVADMYRDGVSPEPGRQTLPTVTIIPLWGINTRVTRPKPFSQKISYGD |
| 37 | 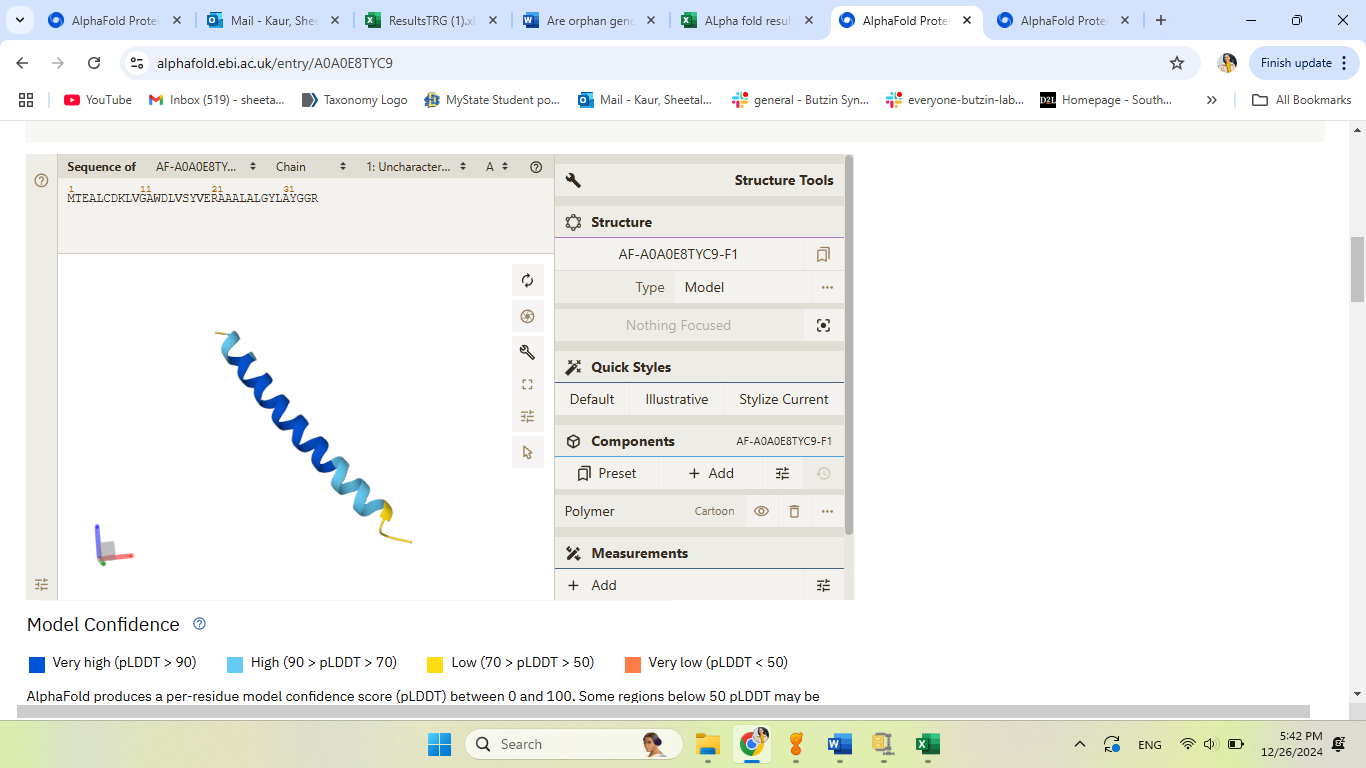 | *M. tuberculosis* | MTEALCDKLVGAWDLVSYVERAAALALGYLAYGGR |
| 38 | 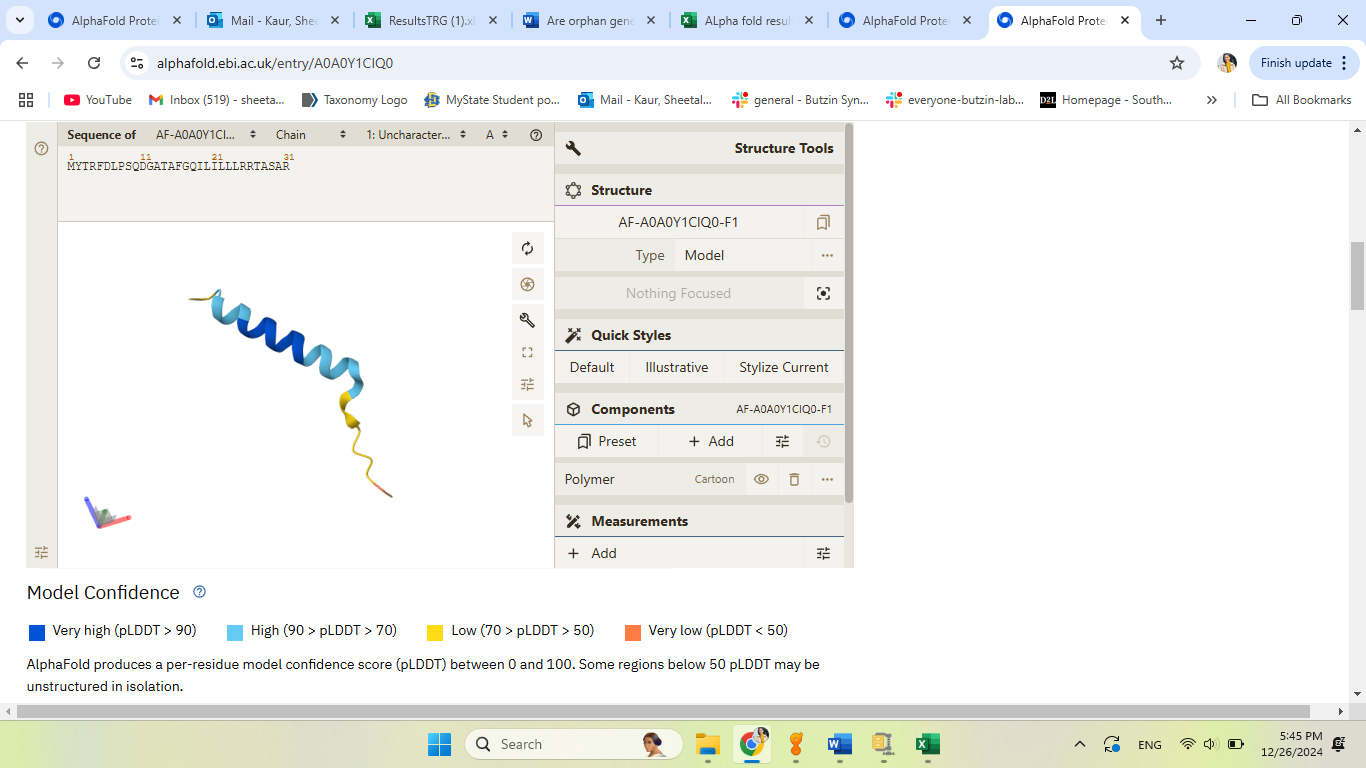 | *M. tuberculosis* | MYTRFDLPSQDGATAFGQILILLLRRTASAR |
| 39 | 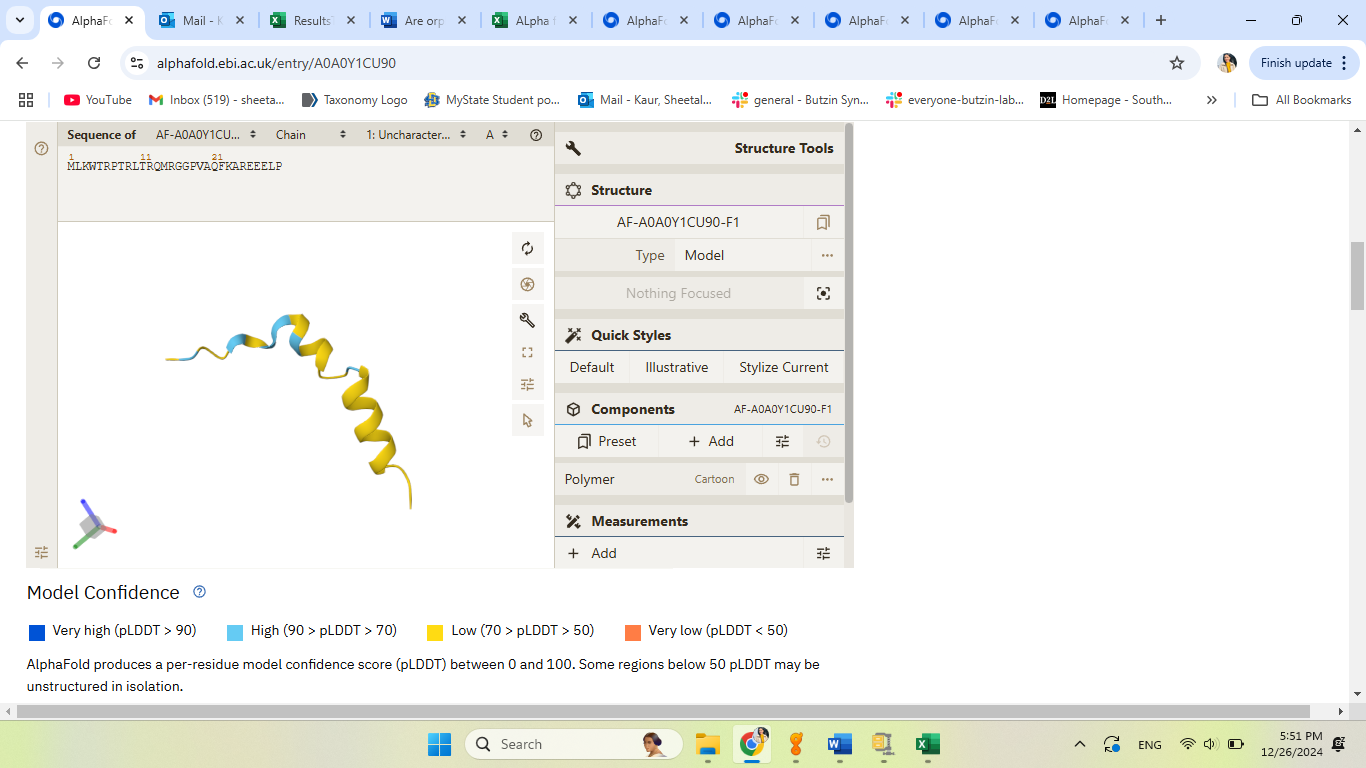 | *M. tuberculosis* | MLKWTRPTRLTRQMRGGPVAQFKAREEELP |
| 40 | 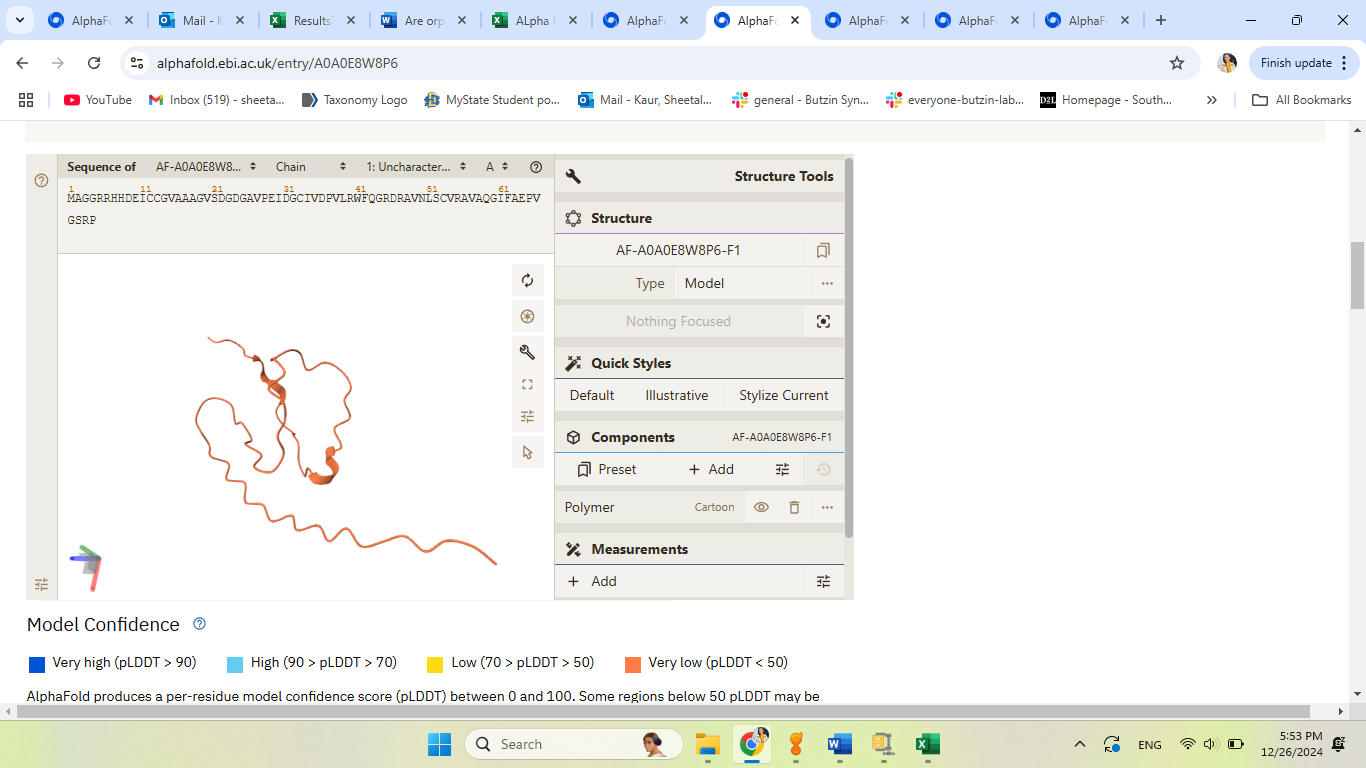 | *M. tuberculosis* | MAGGRRHHDEICCGVAAAGVSDGDGAVPEIDGCIVDPVLRWFQGRDRAVNLSCVRAVAQGIFAEPVGSRP |
| 41 | 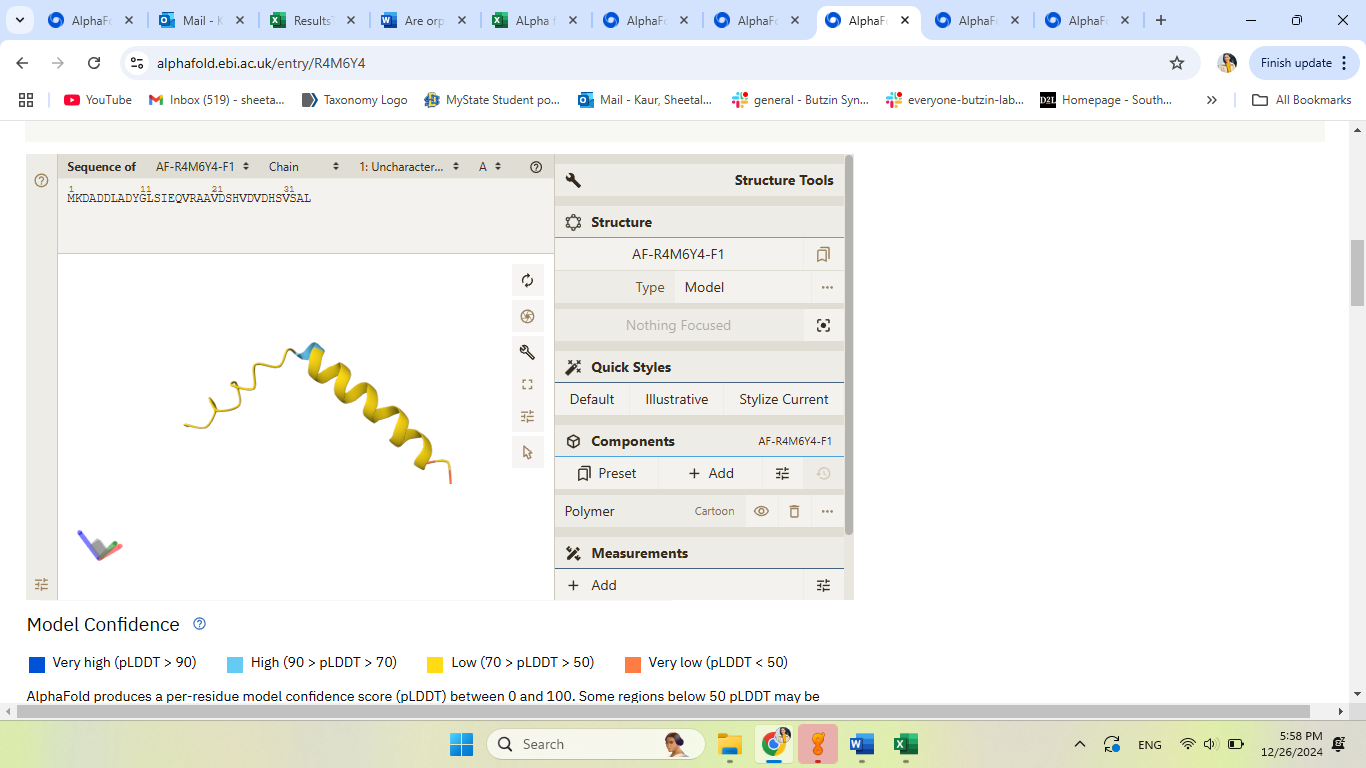 | *M. tuberculosis* | MKDADDLADYGLSIEQVRAAVDSHVDVDHSVSAL |
| 42 | 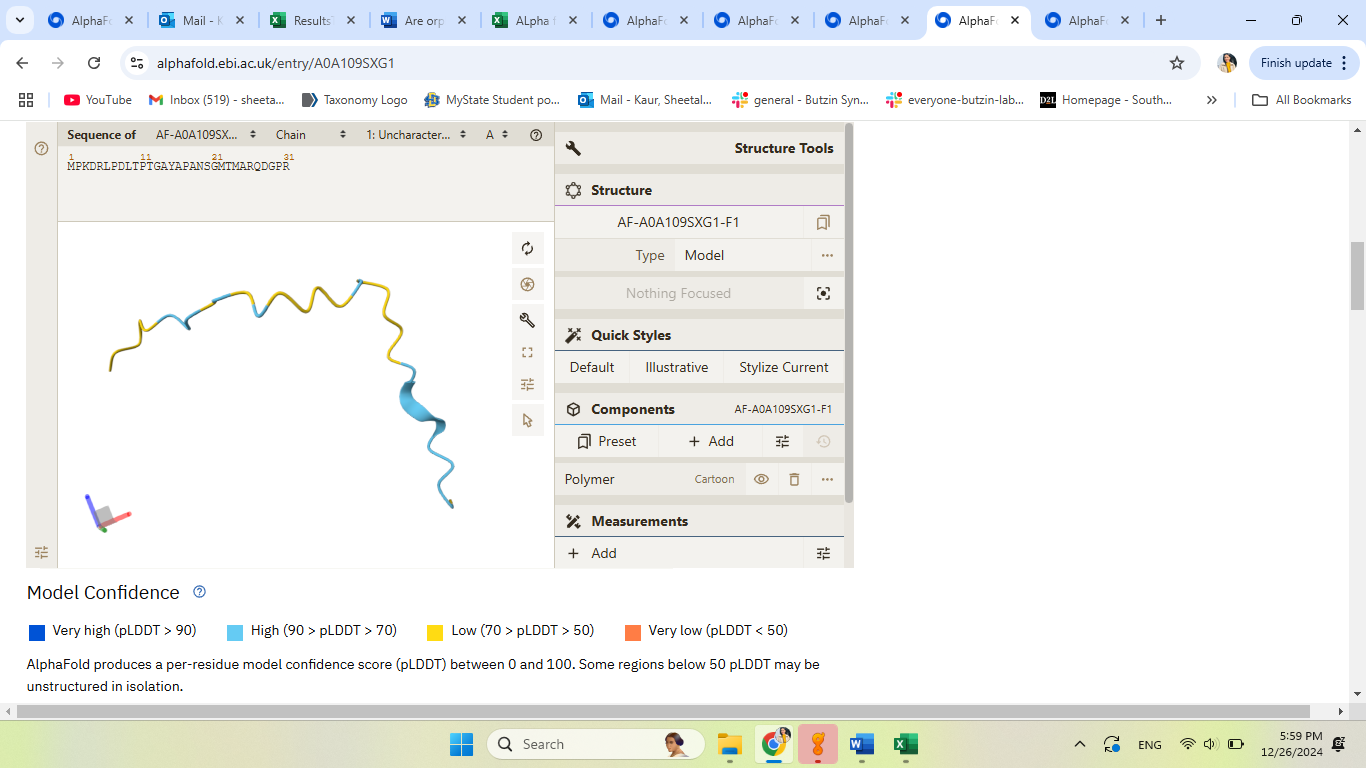 | *M. tuberculosis* | MPKDRLPDLTPTGAYAPANSGMTMARQDGPR |
| 43 | 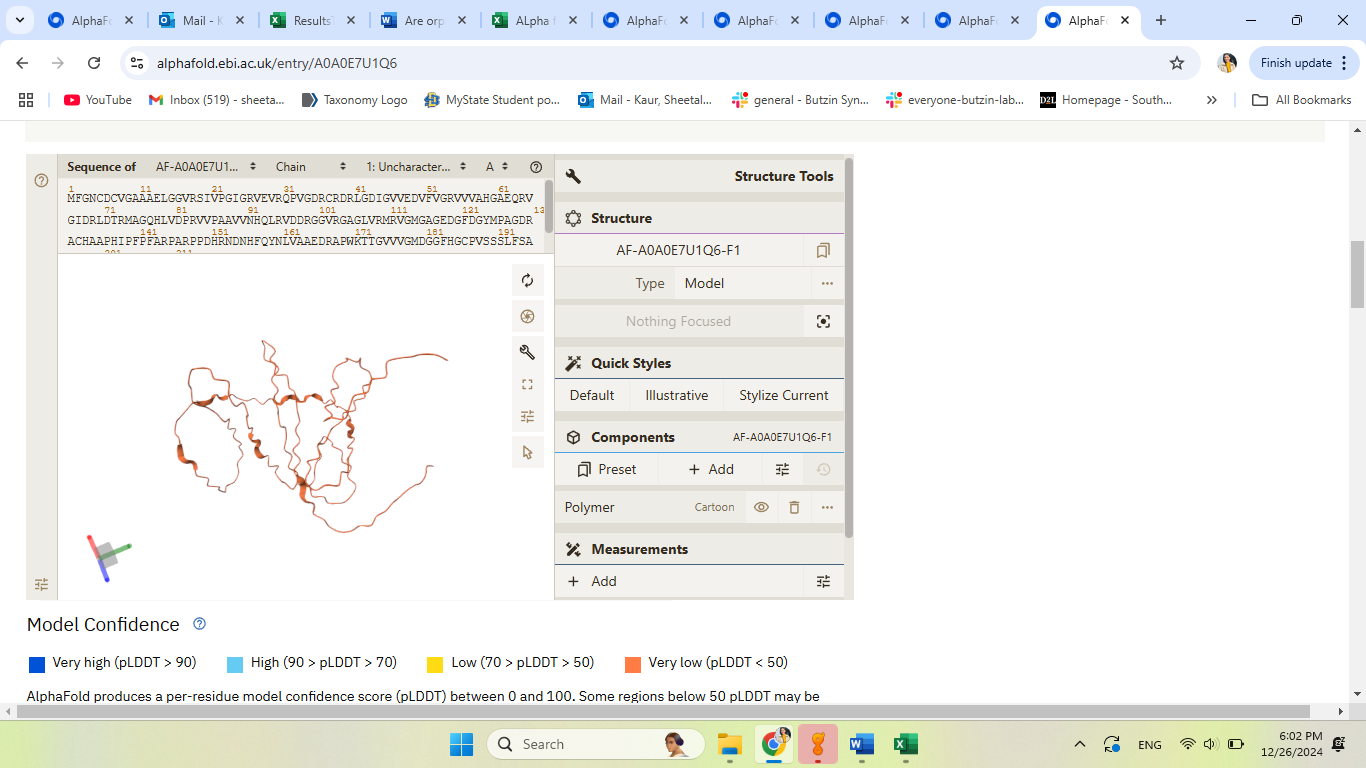 | *M. tuberculosis* | MFGNCDCVGAAAELGGVRSIVPGIGRVEVRQPVGDRCRDRLGDIGVVEDVFVGRVVVAHGAEQRVGIDRLDTRMAGQHLVDPRVVPAAVVNHQLRVDDRGGVRGAGLVRMRVGMGAGEDGFDGYMPAGDRACHAAPHIPFPFARPARPPDHRNDNHFQYNLVAAEDRAPWKTTGVVVGMDGGFHGCPVSSSLFSAVTPWAGIGSPHGSWCH |
| 44 | 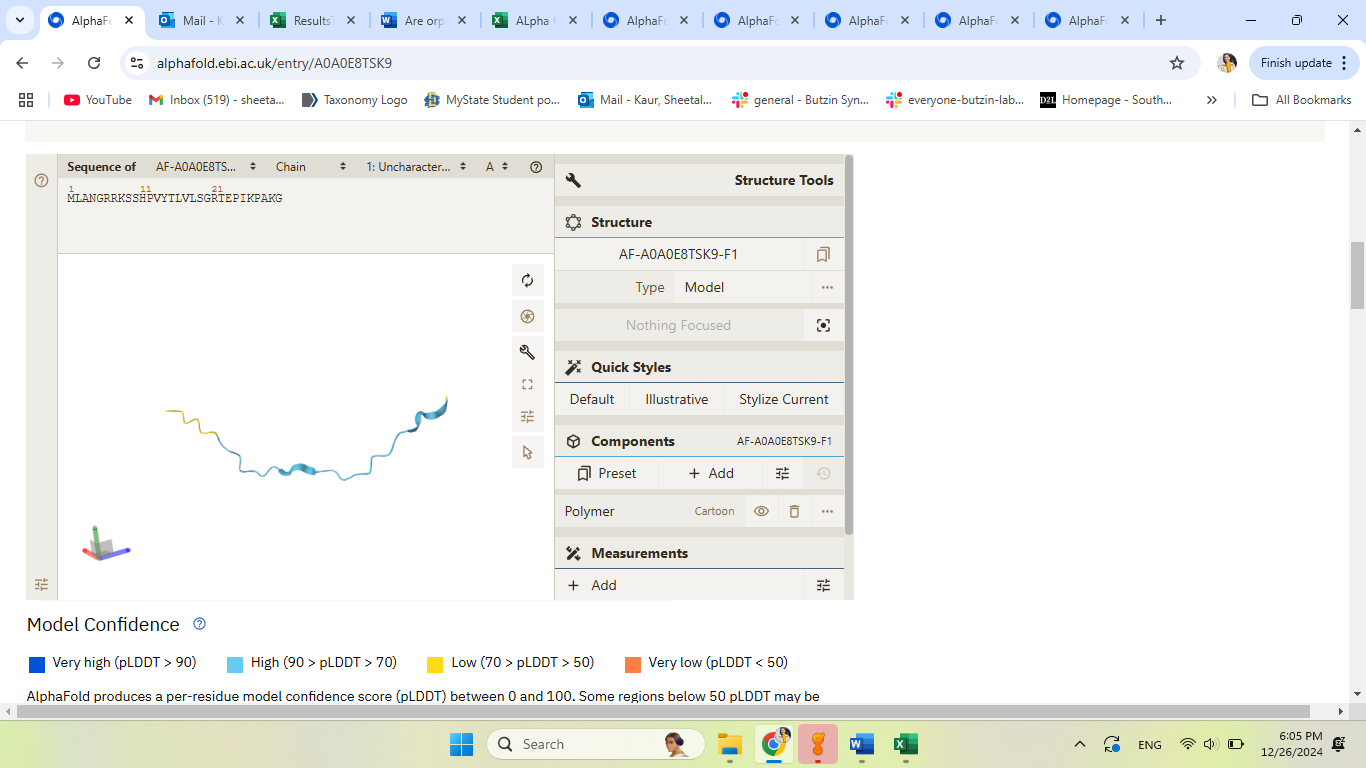 | *M. tuberculosis* | MLANGRRKSSHPVYTLVLSGRTEPIKPAKG |
| 45 | 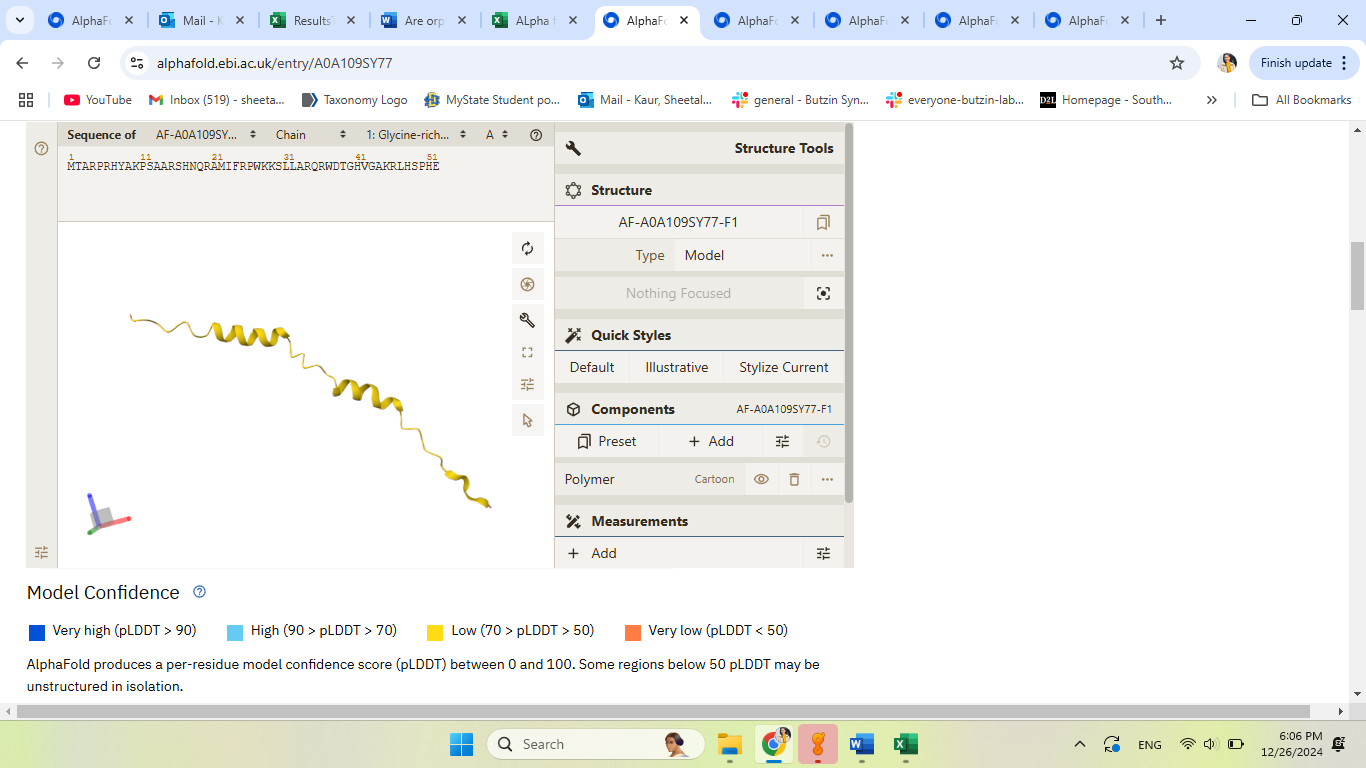 | *M. tuberculosis* | MTARPRHYAKPSAARSHNQRAMIFRPWKKSLLARQRWDTGHVGAKRLHSPHE |
| 46 | 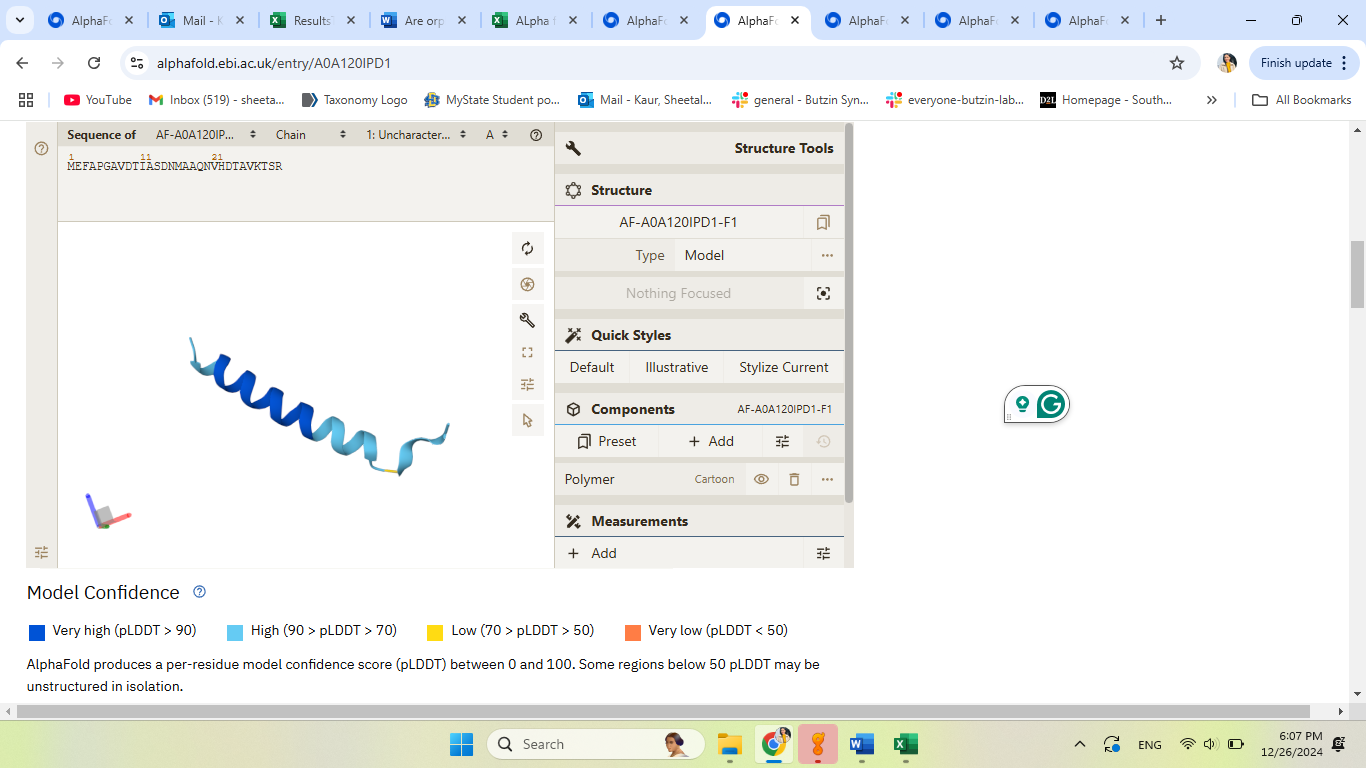 | *M. tuberculosis* | MEFAPGAVDTIASDNMAAQNVHDTAVKTSR |
| 47 | 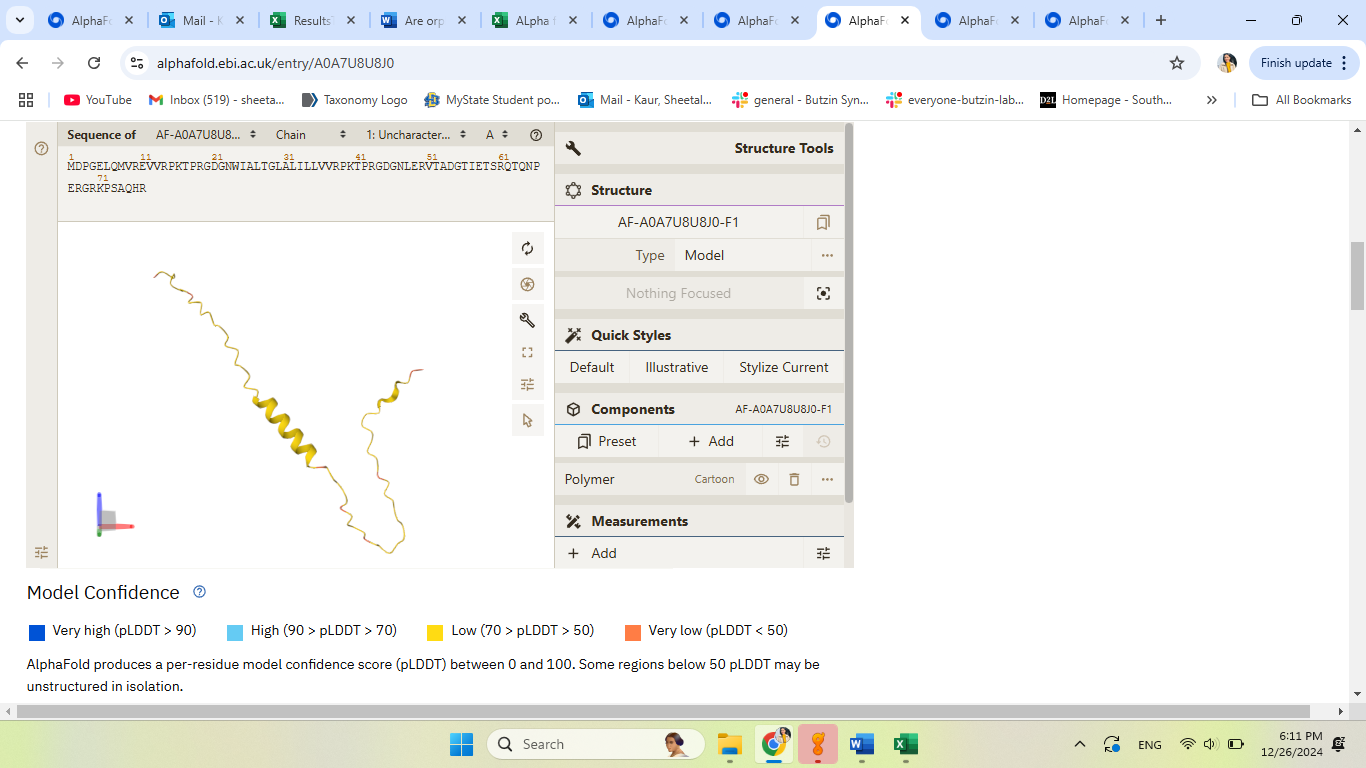 | *M. tuberculosis* | MDPGELQMVREVVRPKTPRGDGNWIALTGLALILVVVRPKTPRGDGNLERVTADGTIETSRQTQNPERGRKPSAQHR |
| 48 | 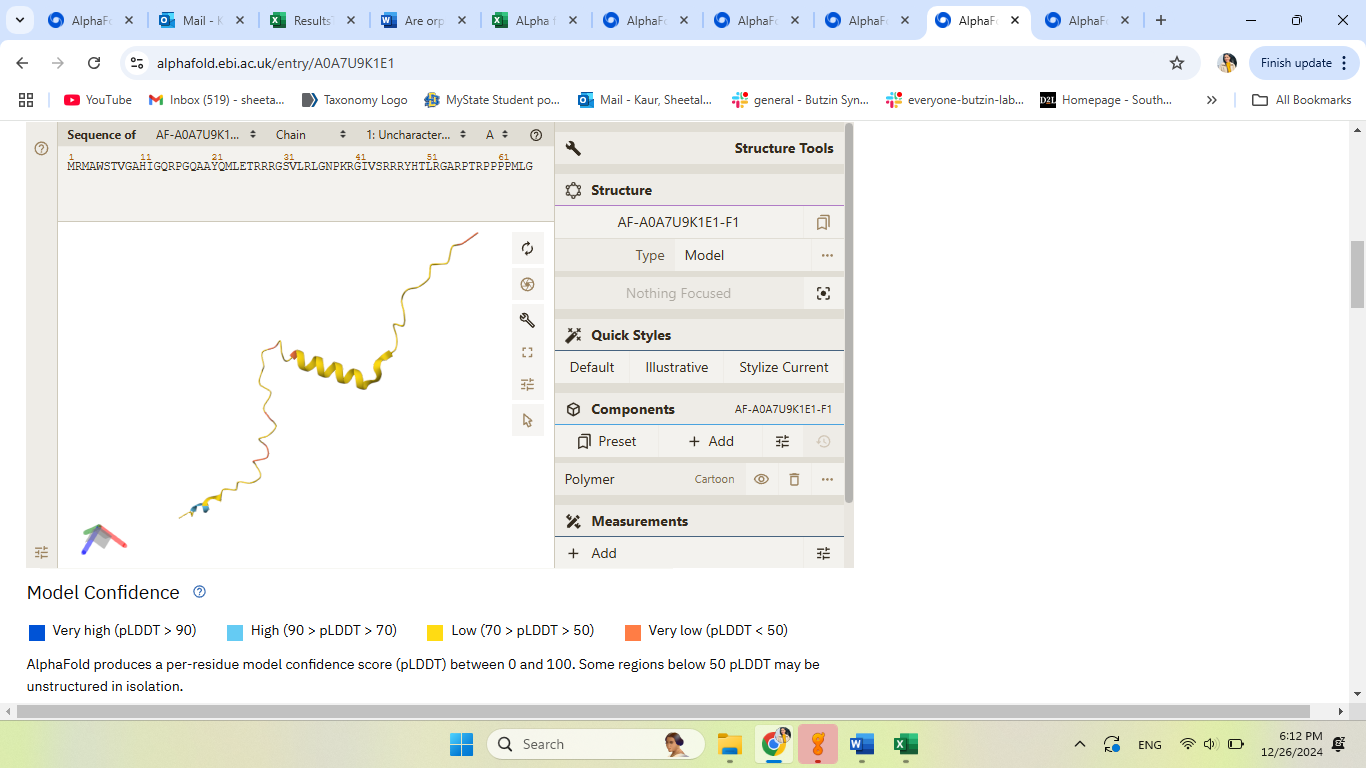 | *M. tuberculosis* | MRMAWSTVGAHIGQRPGQAAYQMLETRRRGSVLRLGNPKRGIVSRRRYHTLRGARPTRPPPPMLG |
| 49 | 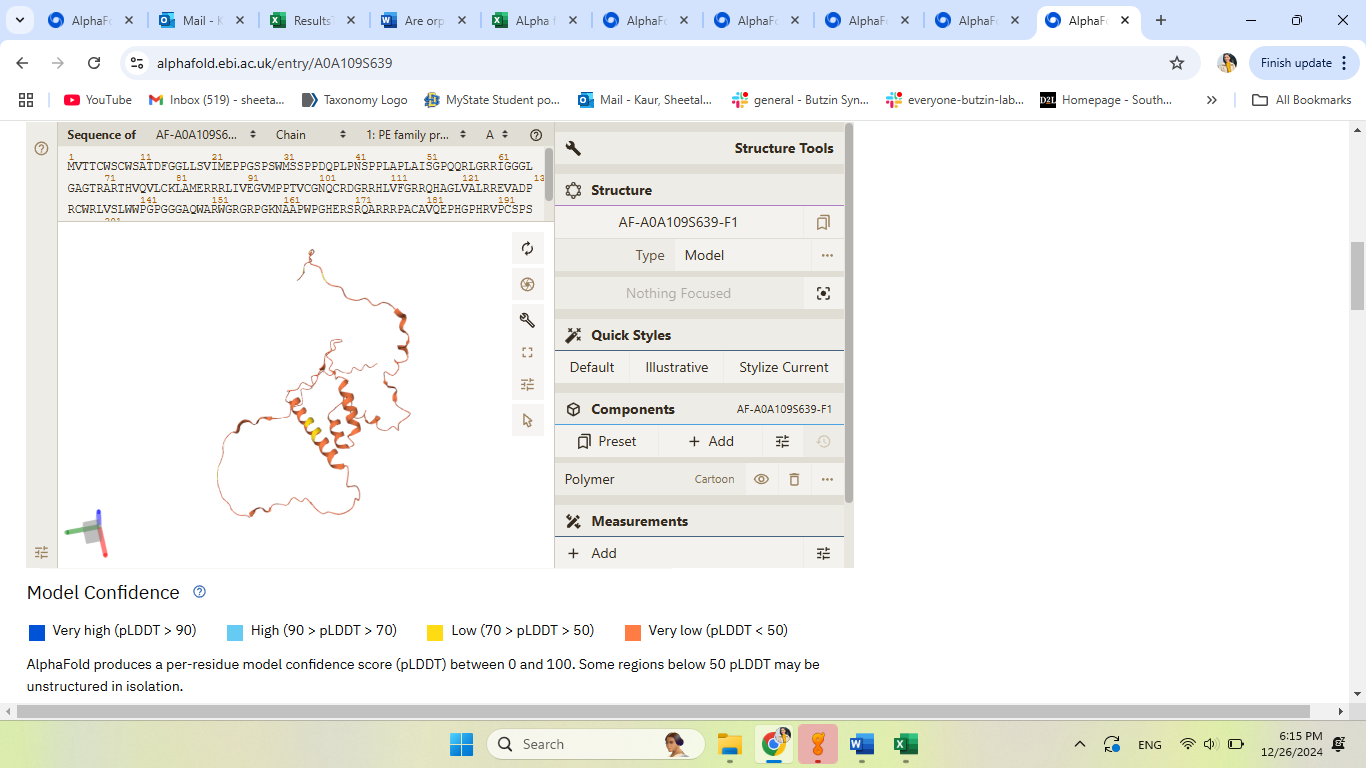 | *M. tuberculosis* | MVTTCWSCWSATDFGGLLSVIMEPPGSPSWMSSPPDQPLPNSPPLAPLAISGPQQRLGRRIGGGLGAGTRARTHVQVLCKLAMERRRLIVEGVMPPTVCGNQCRDGRRHLVFGRRQHAGLVALRREVADPRCWRLVSLWWPGPGGGAQWARWGRGRPGKNAAPWPGHERSRQARRRPACAVQEPHGPHRVPCSPSAATEPVSP |
| 50 | 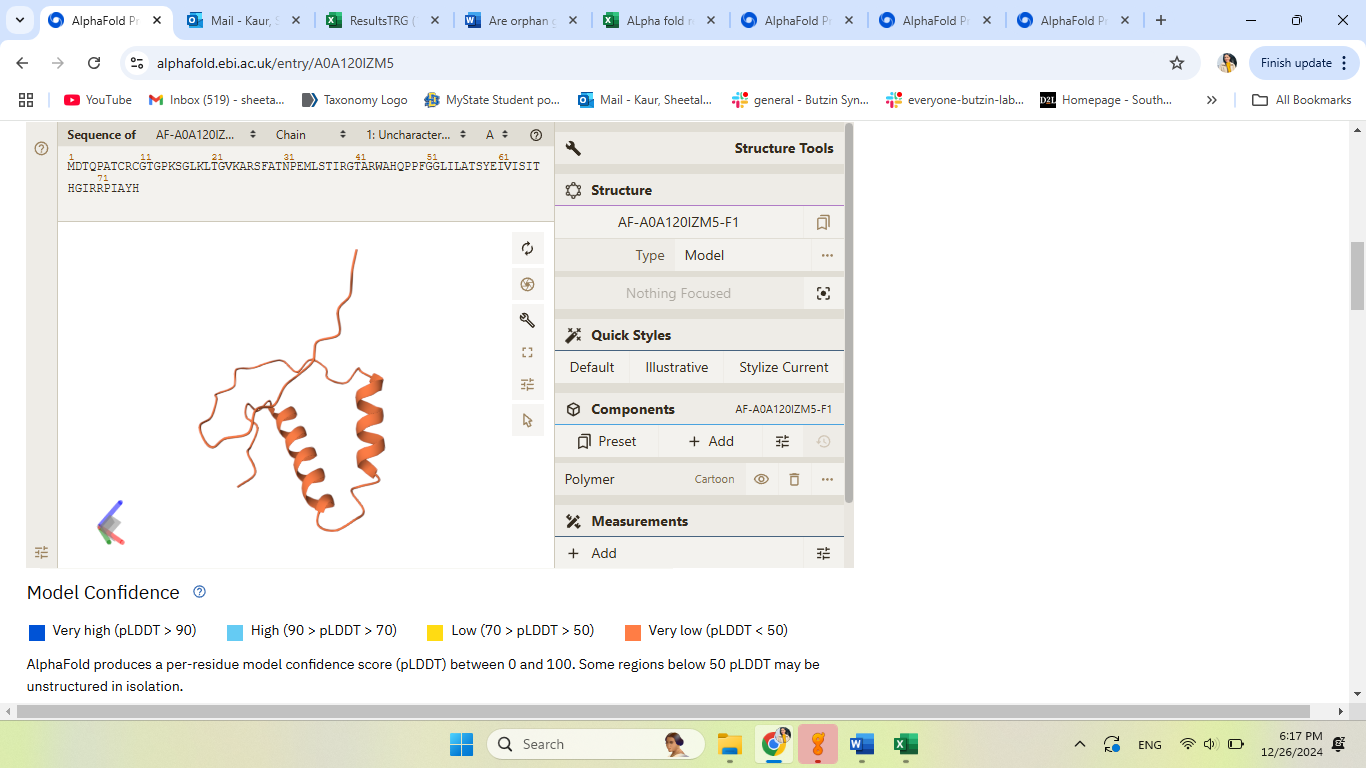 | *M. tuberculosis* | MDTQPATCRCGTGPKSGLKLTGVKARSFATNPEMLSTIRGTARWAHQPPFGGLILATSYEIVISITHGIRRPIAYH |
| 51 | 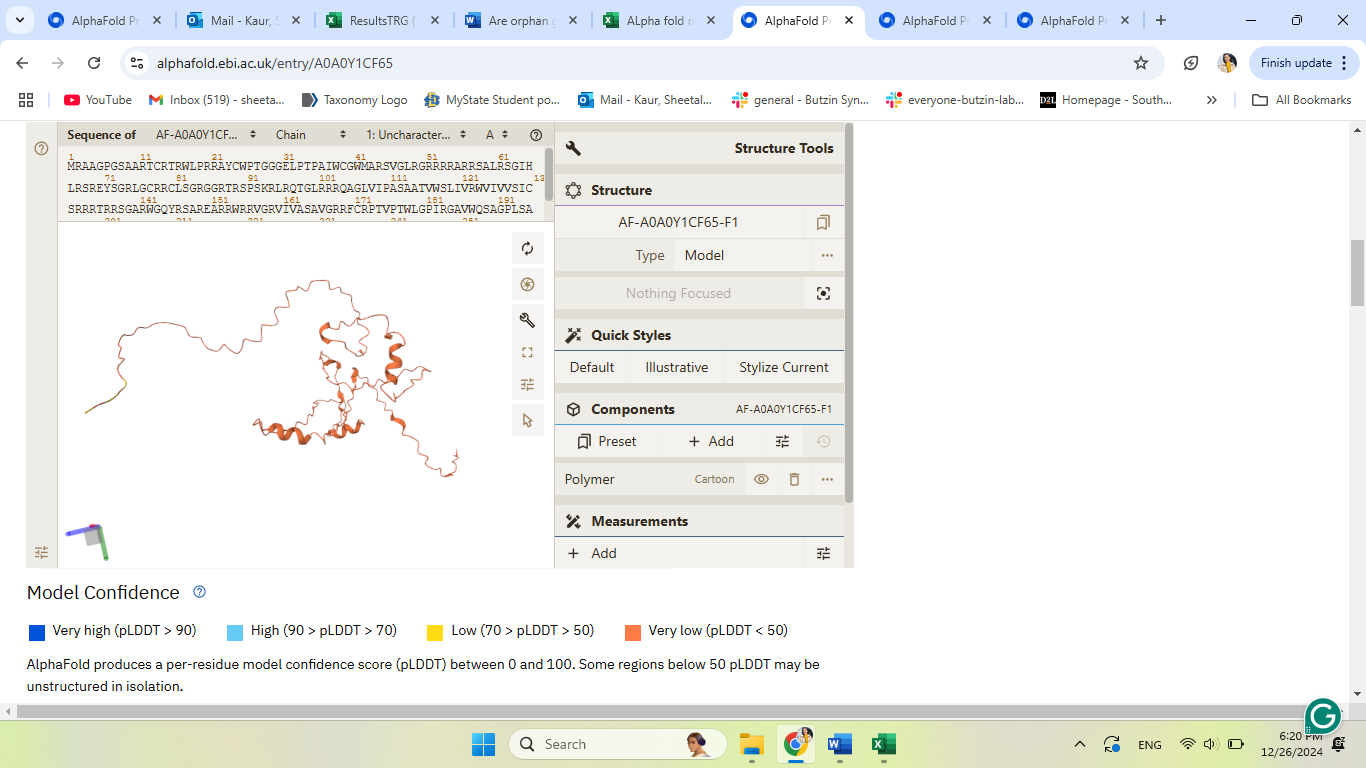 | *M. tuberculosis* | MRAAGPGSAARTCRTRWLPRRAYCWPTGGGELPTPAIWCGWMARSVGLRGRRRRARRSALRSGIHLRSREYSGRLGCRRCLSGRGGRTRSPSKRLRQTGLRRRQAGLVIPASAATVWSLIVRWVIVVSICSRRRTRRSGARWGQYRSAREARRWRRVGRVIVASAVGRRFCRPTVPTWLGPIRGAVWQSAGPLSASCSSSSISSRLNPWDYARLTDETTDTASARYRDSPENDRAGAASRSSARNSAASGR |
| 52 | 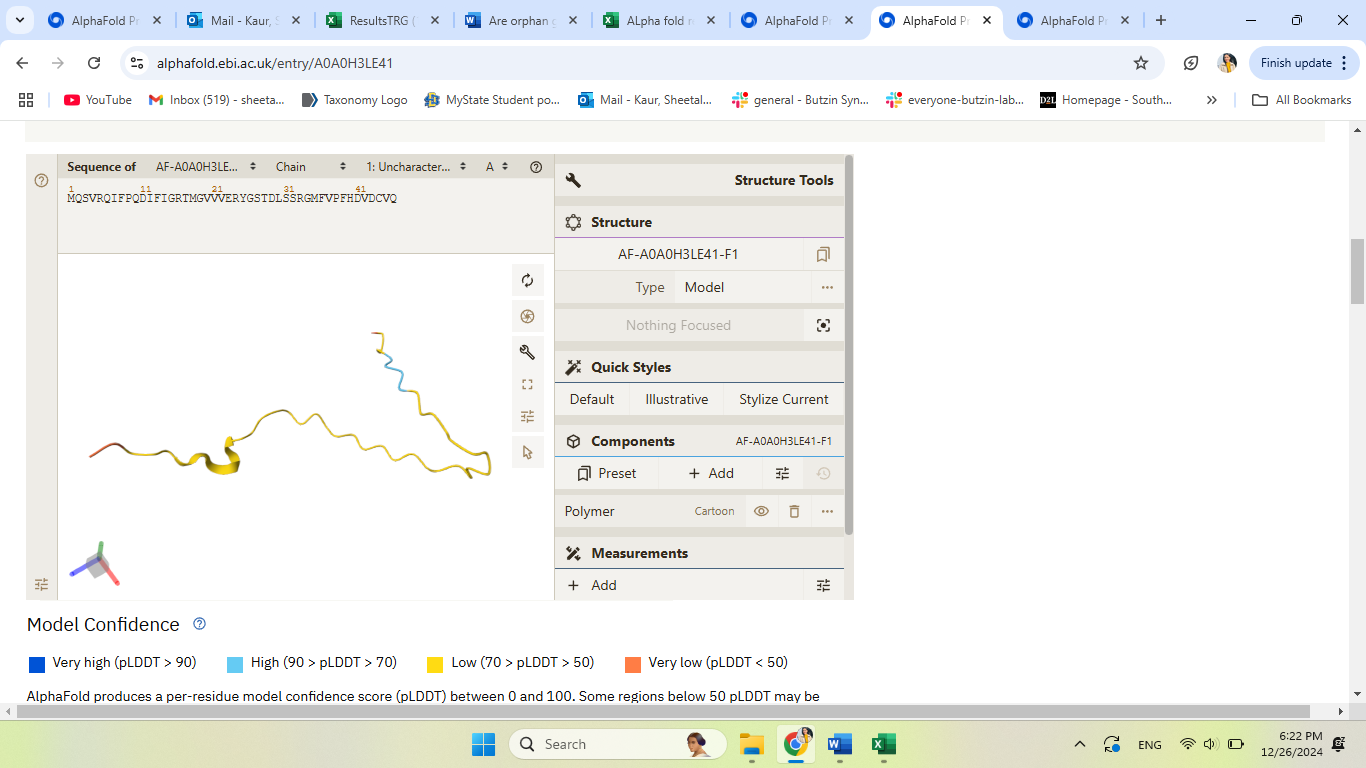 | *M. tuberculosis* | MQSVRQIFPQDIFIGRTMGVVVERYGSTDLSSRGMFVPFHDVDCVQ |
| 53 | 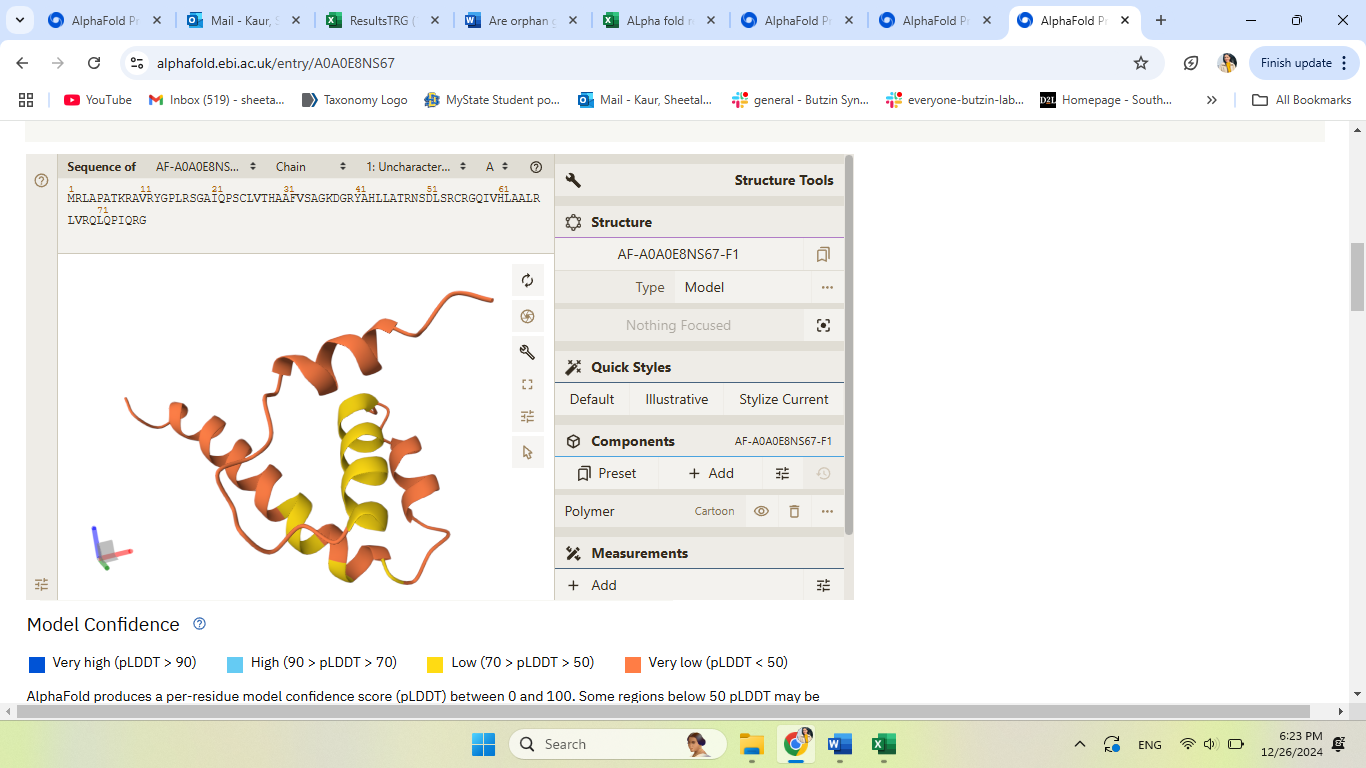 | *M. tuberculosis* | MRLAPATKRAVRYGPLRSGAIQPSCLVTHAAFVSAGKDGRYAHLLATRNSDLSRCRGQIVHLAALRLVRQLQPIQRG |
